# A Riemannian Geometry Theory of Synergy Selection for Visually-Guided Movement

**DOI:** 10.3390/vision5020026

**Published:** 2021-05-25

**Authors:** Peter D. Neilson, Megan D. Neilson, Robin T. Bye

**Affiliations:** 1 School of Electrical Engineering and Telecommunications, University of New South Wales, Sydney, NSW 2052, Australia; 2 Independent Researcher, late School of Electrical Engineering and Telecommunications, University of New South Wales, Sydney, NSW 2052, Australia; megan.neilson@gmail.com; 3 Cyber-Physical Systems Laboratory, Department of ICT and Natural Sciences, NTNU—Norwegian University of Science and Technology, Postboks 1517, NO-6009 Ålesund, Norway; robin.t.bye@ntnu.no

**Keywords:** Riemannian geometry, computational model, nonlinear dynamics, visual space, stereopsis, visually-guided movement, posture-and-place-encoded memory, movement synergies, behavioral goals, reinforcement learning

## Abstract

Bringing together a Riemannian geometry account of visual space with a complementary account of human movement synergies we present a neurally-feasible computational formulation of visuomotor task performance. This cohesive geometric theory addresses inherent nonlinear complications underlying the match between a visual goal and an optimal action to achieve that goal: (i) the warped geometry of visual space causes the position, size, outline, curvature, velocity and acceleration of images to change with changes in the place and orientation of the head, (ii) the relationship between head place and body posture is ill-defined, and (iii) mass-inertia loads on muscles vary with body configuration and affect the planning of minimum-effort movement. We describe a partitioned visuospatial memory consisting of the warped posture-and-place-encoded images of the environment, including images of visible body parts. We depict synergies as low-dimensional submanifolds embedded in the warped posture-and-place manifold of the body. A task-appropriate synergy corresponds to a submanifold containing those postures and places that match the posture-and-place-encoded visual images that encompass the required visual goal. We set out a reinforcement learning process that tunes an error-reducing association memory network to minimize any mismatch, thereby coupling visual goals with compatible movement synergies. A simulation of a two-degrees-of-freedom arm illustrates that, despite warping of both visual space and posture space, there exists a smooth one-to-one and onto invertible mapping between vision and proprioception.

## 1. Introduction

While there is much evidence that natural behaviour is organized into a chain of multisensory goals and that a series of small discrete movements are planned and strung together into a continuous sequence to achieve those goals, we do not yet have a formal mathematical theory of the underlying neural computational processing involved. Our aim in this paper is to develop such a mathematical theory based on the example of skilled visuomotor task performance.

Sprague and colleagues proposed in 2007 that complex behaviour can be broken down into modules, or subtasks, and that specific visual information is required to plan and perform the action needed for each subtask [1]. We agree, but the complexity of the sensory and motor processes involved in planning and sequencing such actions is daunting. Many of the issues have been known and argued about for decades but an overarching computational theory is still lacking. In 1993 in his editorial introduction to a collected work on multisensory control of movement Berthoz articulated a number of shared views about what is necessary for its understanding [2]. These can be summarized as follows: that each percept important in movement is based on a configuration of (multimodal) sensory cues; that perception and movement have to be studied within the 3D space of the environment of living organisms; that reference frames, coordinate transformations and relations between spatial and temporal coding must be addressed; and that the problem of reduction of degrees of freedom and redundant mapping between coordinate systems must be incorporated. He went on to remark on the move from the older view of error-detection between motor command and sensory feedback to one in which the brain actively preselects expected sensory states and detects errors between ‘internal models’ of both the mechanical properties of the body and of physical space. He observed that this feedforward conceptualization must agree with the fact that movement is not continuously controlled but that discrete intermittent processes are involved. All these points remain salient for any theory attempting to describe the processes involved in the planning and execution of actions to achieve behavioural goals.

More recently, in her major review of how vision and action play out during natural behaviour, Hayhoe likewise raised topics that have to be addressed in a mathematical theory of vision and action [3]. These include: the brain’s internal reward circuitry; the mathematics of reinforcement learning; optimal feedback control; the role of uncertainty; the role of memory; visual search; Bayesian weighting of memory; self motion and the parsing of optical flow; and the need for prediction. The mathematical theory presented here concerning the selection and sequencing of minimum-effort, multi-joint, coordinated movements compatible with visual goals has been developed with awareness of the many issues outlined above. Likewise it has been developed cognizant of other theoretical models that seek to understand how the many biomechanical and muscular degrees of freedom (DOFs) of the human body are coordinated to achieve a specific goal. These include the uncontrolled manifold hypothesis, Donders’ law, the minimum-jerk model, the minimum-work model, the minimum torque-change model and stochastic optimal control. A review of these models can be found in [4].

In particular our proposal deals with two complications little mentioned in previous work. First, these movements have to be planned and executed so as to achieve visual goals that are perceived in nonlinear ‘warped’ visual space. The theory must therefore incorporate the geometry of this space as an integral part of the generation of a visually-guided action. Second comes the complication that the human body moving in a gravitational field in sensory and mechanical interaction with its environment is an example of a changing, uncertain, multi-degrees-of-freedom, redundant, nonlinear, dynamical system with limited central processing resources. Nonlinear differential equations describing such stochastic systems are poorly understood and their properties are still an area of active research in the field of mathematics. Our solution to both problems involves a geometric approach that has been rarely applied as yet in perception-action science (see [5] Section 2.2 for a review of theoretical and experimental applications of the geometric approach). We hold that the fields of *differential geometry* and *Riemannian geometry* in particular provide the most suitable mathematical framework for describing the nonlinear computational processes underlying the perception-action decisions required to achieve behavioural goals.

In this paper we combine our previous separate applications of Riemannian geometry to action [5] and to vision [6] to develop a Riemannian geometry theory of computational processes required in the planning and execution of minimum-effort visually-guided movement synergies to achieve specified visual goals. In so doing we construct a somatosensory-hippocampal-visual map of the body and describe its instantiation in visuospatial memory. In other words, we are proposing a means by which the visual system links perception to action. To our knowledge this is the first attempt to establish a workable theoretical account of the visuomotor integration of posture, place and vision that we know to exist both neurophysiologically and behaviourally.

Meanwhile, we also know that those working in these fields, even if mathematically and computationally knowledgeable, will not necessarily be familiar with Riemannian geometry. Therefore, in Section 2 we provide an overview that seeks to explain, using minimal mathematics, why this geometry is so pertinent to vision science and to visuomotor science in particular. For those wishing to venture further we include a tutorial appendix on the major concepts of this remarkable geometric tool. We also direct the reader to our two previous papers [5,6] where the separate applications of Riemannian geometry to synergy formation and to perceived visual space are given in full detail.

The following outline provides a road map of the ensuing sections of this paper:

Section 2: Why Riemannian geometry? A descriptive overview provides intuitive illustrations of the theory and of the relevance of Riemannian geometry.

Section 3: Background. We summarize our previous two papers [5,6] concerning application of Riemannian geometry to analysis of action and vision. This includes a more detailed approach to the Riemannian geometry used in the theory.

Section 4: Here we take the previous *place-encoded* theory of visuospatial memory [6] in which visual images of the environment as seen from different places in the environment are stored in corresponding partitions of visuospatial memory and extend it to a *posture-and-place-encoded* theory in which visual images of one’s own body seen in different postures are added to the visuospatial partitioning. The result is a geometric (fibre-bundle) structure of partitioned visuospatial memory that stores these place-and-posture-encoded visual images to provide a 3D representation of the environment and of the body in that environment as seen from any place and in any posture.

Section 5: We describe the Riemannian geometry of minimum-effort movement synergies (i.e., minimum-effort multi-joint coordinations) for visual tasks with N≤10 control degrees of freedom (CDOFs). This geometric account of the process of *spatial response planning* (i.e., selecting an appropriate movement synergy compatible with a perceptual goal) is accompanied by a brief description of temporal response planning (i.e., planning sequences of goal-directed movement trajectories within the selected movement synergy).

Section 6: Here we present the Riemannian geometry of proprioception-to-vision and vision-to-proprioception maps taking into account redundancy between the many elemental movements of the body sensed proprioceptively and the three dimensions of visual space. We include a Matlab/Simulink simulation of a two-DOF arm moving in the horizontal plane to illustrate that, despite nonlinearities and redundancies and the nonlinear warping of both posture space and visual space, minimum-effort movements of the two-DOF arm can be mapped in a one-to-one, onto and invertible fashion into 3D visual space.

Section 7: We address the Riemannian geometry involved in the selection of task-related movement synergies and describe a model-based reinforcement learning mechanism that uses an *error-reducing association memory network* to associate specified visual goals with compatible low-dimensional minimum-effort movement synergies.

Section 8: We recap the main points of the integrated Riemannian geometry theory and discuss each in relation to other work extant in the literature. In particular we relate Riemannian geometry to work on motor synergies, optical flow, and dissociation of perception and action in illusions.

## 2. Why Riemannian Geometry?

The planning and execution of minimum-effort coordinated multi-joint movements to achieve specified visual outcomes involve nonlinear dynamical computational processes that are complicated to say the least. In this section, using minimal mathematics, we provide an intuitive overview of our application of Riemannian theory to the selection of movement synergies (i.e., multi-joint coordinated movements) compatible with specified visual goals.

### 2.1. The Relevance of Riemannian Geometry in Visual Science

For centuries artists, philosophers and scientists have speculated about the geometry of 3D visual space. There has long been a wealth of formal experimental evidence demonstrating that what we perceive is a warped version of the geometry of the actual physical world [7,8,9,10,11,12,13,14,15,16,17,18,19,20]. However the results of these experiments are inconsistent, leading to the conclusion that the geometry of perceived space is task dependent, varying according to many contextual factors that affect spatial judgement [21,22,23,24,25,26,27,28,29,30,31,32]. This inconsistency has led some to question or even abandon the concept of visual space [33,34]. Others have argued that there really is only one sensory visual space but that it has a cognitive overlay in which observers supplement perception with their knowledge of how distance affects size [18,35,36,37,38,39,40]. We agree. We hold that the variation in geometries of visual space measured experimentally can be attributed to top-down cognitive mechanisms of depth perception perturbing an underlying Riemannian space, a visual space given by the invariant geometry derivable mathematically from the relationship between the size of an image on the retina and the Euclidean distance between the nodal point of the eye and the object in the environment. Therein lies a basis for distinguishing the sensory and cognitive components in geometries of visual space measured experimentally [41].

It is well established that the size of overlapping retinal hyperfields on the retina increase from small in the fovea to large in the periphery [42] while the hypercolumns in the primary visual cortex (V1) to which the retinal hyperfields connect in a retinotopic fashion do not overlap and are all the same size. Consequently, a much larger area of V1 is involved in processing foveal images than in processing peripheral images. The resulting warping of areas of cortical representation defined by topological maps between the retina and the visual cortex is well known. Less well recognized is the warping of visual images defined by topological maps between objects in the 3D environment and their representation in the visual cortex created by the size-distance relationship of images projected onto the retinas. This gives rise to a warped geometry of 3D visual space that is attributable solely to the anatomy and physiology of the eye. It is thus invariant. In 2018 we derived this geometry mathematically from the size-distance relationship and labeled it “Riemannian”. As outlined in Appendix A a Riemannian *manifold* is a topological *space* endowed with a specified set of geometric properties including size, shape and curvature. It is not unreasonable therefore to use the terms “manifold” and “space” interchangeably as we have done in this paper but strictly speaking warped (curved) spaces with measures of size are *Riemannian manifolds*. Just as Riemannian geometry describes for physicists the intrinsic warping of space-time [43] it similarly describes for visual scientists the intrinsic warping of 3D visual space.

The following illustration may be helpful to someone new to this geometry. Consider a marble rolling on a flat surface. The marble rolls in a straight line. Now consider a surface curved like a bowl. The marble now follows a curved pathway driven by the curvature of the surface. Analogously, think of an object moving at constant speed along a straight line in flat Euclidean 3D space. In warped (i.e., curved) 3D visual space the object appears to follow an accelerating curved pathway because of the intrinsic curvature of 3D visual space. An object approaching at constant speed not only appears to loom in size but it also appears to accelerate as it approaches. Conversely, suppose an object moving in flat 3D Euclidean space appears to be moving in a straight line at constant speed. For this to happen the object has to actually follow an accelerating curved pathway in flat 3D Euclidean space in order to compensate for the intrinsically warped geometry of visual space introduced by the anatomy and physiology of the eye. Clearly this warping of visual space has to be taken into account when planning visuomotor tasks, for example, catching a ball.

Another analogy may be useful. Imagine the inside surface of a bowl covered with stick-on stamps of equal size. Now imagine viewing the bowl from above but with the curvature of the bowl ignored so that it appears as a flat disc. The stamp at the middle of the disc will appear the largest but moving out towards the periphery the stamps will appear to shrink in size with distance from the centre because of the curvature of the bowl. A similar shrinking in size occurs for the images of objects in 3D visual space as their distance from the egocentre increases because of the curvature of visual space introduced by the eye. In Section 3.1 we provide more detailed description of the warping of 3D visual space caused by the size-distance relationship of images projected onto the retinas. Meanwhile suffice it to say that Riemannian geometry provides the theoretical tools needed to compute the apparent size and the apparent position, velocity, acceleration and curvature at every point along every pathway, curved or not, in flat 3D Euclidean space.

### 2.2. The Relevance of Riemannian Geometry in Action Science

The human body has about 110 elemental movements that can be varied voluntarily independently of each other one at a time. Each elemental movement can be sensed proprioceptively. We define *posture space* (or proprioceptive space) to be the 110-dimensional space spanned by the 110 elemental movements. We define the *configuration space* of the human body to be a 116-dimensional space equal to the Cartesian product of the 110-dimensional posture space, the 3-dimensional *place space* giving the position (or place) of the head in the 3D environment measured with respect to an external (allocentric) reference frame $\left(X,Y,Z\right)$, and the 3-dimensional *orientation space* giving the three rotation angles of the head relative to the external reference frame $\left(X,Y,Z\right)$. A movement to achieve a perceptual goal can be thought of as a trajectory in configuration space. This can involve not only a change in posture of the body but also a change in the place and orientation of the head in the environment.

Neglecting relatively small frictional forces there are two main changing loads on functional muscles that determine the pattern of muscle activation required to produce a specified movement trajectory in configuration space: (i) the gravitational loads on functional muscles vary as a function of the configuration of the body taking the changing distribution of support forces acting on the body into account and (ii) the mass-inertia loads about each elemental movement vary as a function of the configuration of the body taking mechanical interactions with support surfaces and objects in the environment into account. It is well known (thanks to orbiting space station experiments) that the nervous system can adapt to changes in gravity. Basically this involves the nervous system learning the patterns of muscle activations needed to hold the body in every possible configuration. But how does the nervous system handle the changing mass-inertia loads about each elemental movement? Obviously it must learn the mass-inertia load about each elemental movement in every possible configuration of the body. But this is not sufficient!

Consider the simple case of a two-DOF arm moving in the horizontal plane (we will set out the equations for such an arm in Section 6). The mass-inertia load about the shoulder-angle varies as a nonlinear function of the elbow-angle. The distance between the shoulder and the centre of mass of the forearm changes with changes in elbow-angle. Consequently the moment of inertia of the arm about the shoulder-angle (i.e., the distribution of mass about the axis of rotation at the shoulder) varies as a function of elbow-angle. But if the arm is rotated at the shoulder it generates centrifugal forces that cause the forearm to fling outwards thereby changing the mass-inertia load at the shoulder. Thus movement of a two-DOF arm involves a complicated, nonlinear, dynamical, mass-inertia interaction between the two joint-angles.

This becomes considerably more complicated, one might even say impossibly complicated, when all the 116-dimensions of the configuration space of the body (including mechanical interactions between the body and the environment) are taken into account. But this of course is exactly what the nervous system does. Even a simple one-DOF movement such as abducting the arm at the shoulder requires generation of a synergy of muscle activations distributed throughout the entire body to facilitate the abduction movement and to compensate for the unwanted mass-inertia interactions with all the other elemental movements of the body. These interactions change as the distribution of support forces on the body change and they have to be predicted particularly when planning fast ballistic movements.

To make an energy efficient multi-joint coordinated movement to achieve a specified visual goal the nervous system has to plan and execute a minimum-effort multi-joint coordinated movement trajectory in the configuration space of the body to move between a specified initial configuration and a specified final configuration in a specified time compatible with the specified visual goal. How can such a trajectory be planned so easily within a single reaction time interval given the complexity of the mass-inertia interactions between the elemental movements of the body and the environment? The answer lies in Riemannian geometry!

As explained in Appendix A Section A.11, the mass-inertia matrix of the body corresponds to the kinetic-energy Riemannian metric on the posture space of the body. According to the theorems of Riemannian geometry and classical mechanics this changing kinetic-energy metric can be represented by curvature of posture space (see A.16). The Riemannian geometry theory of geodesics and parallel translation (A.17) applied to curved posture space generates a natural, free motion, minimal muscular-effort trajectory in posture space known as a geodesic trajectory that takes all the mass-inertial interactions between elemental movements into account. What is more, a Riemannian geometry formulation allows the computations to be broken down into a set of relatively simple distributed point-calculations that can be performed simultaneously in parallel. Thus an understanding of Riemannian geometry leads to a straightforward solution of a key problem of nonlinear dynamics in action science.

### 2.3. The Geometry of an Integrated Somatosensory-Hippocampal-Visual Memory

To account for central processing underlying the planning and execution of visually-guided movement it is necessary to integrate somatosensory, hippocampal and visual information (i.e., posture, place and vision). In this paper we describe a plausible structure for an integrated somatosensory-hippocampal-visual memory able to combine visual information about the environment and the body in that environment with proprioceptive information about the posture of the body and hippocampal information about the place and orientation of the head. We take it as given that posture of the body is encoded by temporospatial patterns of neural activity in the somatosensory cortex (i.e., in proprioceptive space) and that cells in the hippocampal formation provide an exquisitely detailed representation of the person’s current place and heading in the local environment [44].

The present proposal is conceptually an extension of our previously proposed place-encoded visuospatial memory structure [6]. There, as here, we use the constructs of differential geometry to delineate how the partitions of such a memory are instated and accessed. A key concept in our scheme of early visual processing is that of an *image-point vector* and its association with an *image point*. We propose that during a fixed-gaze interval the image falling on each retinal hyperfield is encoded by a vector of numbers corresponding to the levels of activity induced in a cluster of ocular dominance columns within a cortical hypercolumn [6]. We call this vector of numbers an *image-point vector*. During the same fixed-gaze interval depth-perception mechanisms based on stereopsis, retinal-image disparity and focus control determine the cyclopean coordinates $\left(r,\theta ,\varphi \right)$ for the point in the environment that projects its surrounding image onto a retinal hyperfield. These are the only depth-perception mechanisms that give an absolute estimate of depth based solely on afferent information without intervention by top-down cognitive mechanisms of depth perception. Euclidean distance from the egocentre is denoted by r while the angles $\left(\theta ,\varphi \right)$ give the direction in the 3D environment relative to an external (i.e., allocentric) reference frame $\left(X,Y,Z\right)$. Notice that each point $\left(r,\theta ,\varphi \right)$ is encoded as a mixture of egocentric and allocentric coordinates. We call the cyclopean coordinates $\left(r,\theta ,\varphi \right)$ for each retinal-hyperfield image during each fixed-gaze interval its *image point*.

For the visual system to construct a representation of the entire 3D environment from a sequence of fixed-gaze points (i.e., visual scanning) the encoded image-point vectors and their associated image points acquired during each fixed-gaze interval have to be captured into visuospatial memory before the activity encoded in the cortical hypercolumns is lost and replaced with an encoding of the retinal-hyperfield images for the next gaze point. Via visual scanning the memory accumulates the information from a sequence of gaze points, ultimately providing a representation of the entire 3D environment as seen from every place and posture.

### 2.4. The Street View Analogy

The Street-View feature of Google maps [45] provides a useful analogy for obtaining an intuitive understanding of our proposed structure of the integrated somatosensory-hippocampal-visual memory. To acquire its massive database Google’s Street View deploys a roving car with rotating roof camera to capture images associated with a known place (just like visual scanning enables the capture of image-point vectors with associated image points at each posture and place in our proposal). In the street-view application specification of a street name and house number (or the equivalent geo-coordinates) retrieves from a cloud submemory all the images associated with that place on the map. Each of these is associated with an angle of the camera rotating in a horizontal plane at that place on the map. Thus specifying the angle retrieves a particular view of the surroundings. An effect of depth is obtained by zooming in or out of the retrieved image. Each street-view image is stored efficiently in the submemory using a highly compressed format that removes redundancy from the image, thus minimizing the total amount of memory required.

In our proposal retinal-hyperfield images for multiple gaze points are superimposed and accumulated in each submemory partition thereby constructing an image of the environment and of the body in that environment as seen from that posture-and-place. Neural activity in the somatosensory cortex and hippocampal region of the brain encode the posture of the body and the place and orientation of the head in the environment, respectively. This posture-and-place-encoding is analogous to the geo-coordinates in Street View. It retrieves from a submemory (i.e., a partition of visuospatial memory) all the encoded retinal-hyperfield images associated with that posture and place (i.e., as seen from that posture and that place through visual scanning analogous to the rotating camera in Street View). In other words it retrieves from a submemory an image of the environment and of the body in that environment as seen from that posture and place.

By analogy with the horizontal angle of the camera that took the street-view image, each of the posture-and-place-encoded retinal-hyperfield images (i.e., image-point vectors) in the visuospatial submemory is associated with a cyclopean vector $\left(r,\theta ,\varphi \right)$ (i.e., image point) corresponding to the point in the 3D environment that projects to that retinal hyperfield during that fixed-gaze interval with the head at that place and the body in that posture, orientation of the head having been absorbed into the cyclopean coordinates $\left(r,\theta ,\varphi \right)$.

Analogous to the efficient storage of each street-view image, each posture-and-place-encoded retinal-hyperfield image at each image point $\left(r,\theta ,\varphi \right)$ in the submemory is encoded efficiently as a vector of real numbers (i.e., image-point vector) that removes redundancy from the hyperfield image. For a detailed description of this encoding process based on *singular value decomposition* see ([6] Section 2.7 and Appendix A). This representation of encoded retinal-hyperfield images as image-point vectors associated with image points on the surfaces of visible objects in the environment and on the visible surfaces of the body facilitates the description of the posture-and-place-encoded visual memory as a Riemannian structure. Our full Riemannian geometric account of this memory is given in Section 4.3.

### 2.5. Constructing a 3D Representation via Riemannian Mapping

The place-and-posture-encoded images within each partition of visuospatial memory are endowed with an estimate of depth obtained from stereopsis, retinal-image disparity and focus control mechanisms of depth perception. But when 3D objects in the environment (including the body) are viewed from a fixed posture and place with depth they appear as 2D curved surfaces with boundary (or outline). This is not a 3D representation! Some have described it as a $2\frac{1}{2}$ D representation. Nevertheless, each of the many different partitions of visuospatial memory contains a posture-and-place-encoded image of the same 3D objects and the same body but seen from different places and with the body in different postures. If a sufficiently large number of places and postures are encoded then the totality of all the images in all the partitions of visuospatial memory contain all the information needed to construct a 3D representation of the environment and of the body in that environment. Using a special type of map defined in Riemannian geometry (viz., a vector bundle morphism) between image points and image-point vectors in each and every partition of visuospatial memory it is possible to remove occlusions and to construct a 3D representation of the environment and of the body in that environment seen in the correct perspective from any posture and place. We set this out mathematically in Section 4.3. The point to be made here is that a visuospatial memory with Riemannian structure described has the capability to provide a visualization of moving about within a learned internal representation of the 3D environment. One only has to visualize moving, say from one’s front door to one’s kitchen, to know that this capability exists.

### 2.6. Geodesic Trajectories and Reinforcement Learning

We return now to the Street-View analogy. In Street View one can steer the mouse pointer along streets looking at different street-view images along the way. This can be thought of as selecting a trajectory of street names, house numbers and horizontal camera angles to reach a required image of a particular house or street corner or whatever. Planning and executing such a mouse-pointer trajectory between a specified initial street-view image and a specified final street-view image is mathematically a two-point boundary value problem that is difficult to solve. Likewise to move between a specified initial posture, place and visual image and a specified final visual image of the body in the environment (with the hand grasping a glass for example) one would need to plan a minimum-effort trajectory in posture-and-place space to reach the required end-point visual image. Remember, each posture-and-place along the trajectory is associated with a visual image of the environment and of the body in that environment as seen from that posture-and-place. Again, as with the mouse-pointer trajectory, mathematically this is a high-dimensional, nonlinear, two-point boundary value problem that is difficult to solve. Yet people quickly learn to do it by trial and error, imitation, and perhaps some instruction from an expert. Our proposal is that humans circumvent this difficult computational problem, just as they do in steering the mouse in Google Street View and just as animals in general learn to make movements that achieve desired sensory outcomes. *Reinforcement learning* is used to find the optimal trajectory in posture-and-place space compatible with the specified initial and final visual images. Incorporating the nonlinear warping both of visual space and of posture space the Riemannian geometry theory of geodesic trajectories (A17) provides the mathematical tools needed to describe reinforcement learning in this context. We give a full account of this in Section 7.

### 2.7. Two Streams of Visual Processing

The structure of the posture-and-place-encoded memory described above leads elegantly to the concept of two independent streams of visual processing. Encoded image points $\left(r,\theta ,\varphi \right)$ on the surface of the body change with a change in the posture of the body but do not change as the place of the head (i.e., egocentre) in the environment is changed. Conversely, encoded image points $\left(r,\theta ,\varphi \right)$ for points on the surfaces of objects in the environment (other than the body) change when the place of the egocentre is changed but do not change when the posture of the body is changed.

Some confusion might arise here because rotation of the head about its axis and/or atlas joints, sensed proprioceptively, produces a change in the orientation of the head. But as mentioned earlier, the direction of heading is exquisitely encoded within hippocampal regions of the brain so it is fair to say that, as well as place, the orientation of the head is encoded within hippocampal regions. However different aspects of orientation are encoded in hippocampal regions and somatosensory regions of the cortex. It is possible to change the orientation of the head in the environment by, for example, changing from a standing to a lying configuration without changing the axis-atlas joint angles. Moreover, the cyclopean coordinates $\left(r,\theta ,\varphi \right)$ for any point in the environment are determined by both the angles of the eyes in the head and the orientation of the head in the environment. Thus the orientation of the head can be absorbed into the cyclopean coordinates $\left(r,\theta ,\varphi \right)$.

To illustrate, think of a mannequin in a fixed posture being carried about. The cyclopean coordinates referenced from the point midway between the mannequin’s eyes to points on its body do not change as it is moved about regardless of the fixed joint-angles of its head. In contrast, the cyclopean coordinates referenced from the point midway between the mannequin’s eyes to points on objects in the environment do change as the mannequin is carried from place to place. Regardless of the angle at which the mannequin’s head is set relative to its body the place of its head in the environment can be changed independently of its posture (including the set angle of its head).

The fact that the visual consequences of changes in posture differ from those of changes in place gives rise to the prediction that two streams of visual processing exist independently of each other in parallel. One stream associates image points and image-point vectors for points on the surface of the body with proprioceptive patterns of activity in the somatosensory cortex encoding different body postures. The other stream associates image points and image-point vectors for points on the surfaces of objects (other than the body) in the environment with patterns of activity in the hippocampal region of the brain encoding the place of the head in the 3D environment.

### 2.8. A Riemannian Metric Encodes the Intrinsic Geometry of Visual Space

Having set out intuitively in previous sections the concept of image points and image-point vectors, we call on this to revisit the notion of an invariant visual space introduced in Section 2.1. This time we use some basic equations that underlie the mathematical description of that space. These are a fundamental springboard to the derivations later in the paper.

The relationship between the Euclidean distance r and the size of the retinal-hyperfield image stored at the image point $\left(r,\theta ,\varphi \right)$ in the appropriate visuospatial submemory is represented by a symmetrical, positive definite, 3×3 matrix $g\left(r,\theta ,\varphi \right)$ at each site $\left(r,\theta ,\varphi \right)$ in the submemory. The matrix $g\left(r,\theta ,\varphi \right)$ at each site $\left(r,\theta ,\varphi \right)$, known as a *Riemannian metric* (see A.11)*,* varies smoothly from image point to image point in the submemory. Using the mathematics of Riemannian geometry we can determine the curved (warped) geometry of the 3D visual space encoded by the Riemannian metric and the way it changes from image point to image point.

The matrix

(1) $$g\left(r,\theta ,\varphi \right)=\left[\begin{array}{ccc} \frac{1}{r^{2}} & 0 & 0\\ 0 & \frac{1}{r^{2}} & 0\\ 0 & 0 & \frac{1}{r^{2}} \end{array}\right]$$

at each image point $\left(r,\theta ,\varphi \right)$ in each submemory describes the warped geometry associated with the images of objects decreasing in size in inverse proportion to the Euclidean distance r between the object and the egocentre. Without going into detail the Riemannian geometry works like this. Suppose the image point $\left(r,\theta ,\varphi \right)$ happens to be a point on the outline of a 2D curved image of an object embedded in the 3D intrinsically-warped visual space seen from a fixed place. Suppose there exists a 3D direction vector v in visual space tangent to the boundary curve at that point in 3D visual space. The length (norm) $\left|v\right|$ of that vector in the warped visual space equals the square root of the metric inner product of v, given by the equation

(2) v=〈gr,θ,φv,v〉12.

with $g\left(r,\theta ,\varphi \right)$ set equal to the matrix in Equation (1) at each image point $\left(r,\theta ,\varphi \right)$ in the submemory, the length $\left|v\right|$ varies from image point to image point along the boundary curve in inverse proportion to the Euclidean distance r of that point from the egocentre at the origin. The length L between any two points a and b along the boundary curve in the warped 3D visual space (i.e., the arc length L between points a and b in 3D visual space) is given by the integral of the norm $\left|v\right|$ of the direction vector, i.e.,

(3) L=∫ab〈gr,θ,φv,v〉12ds

thus distances and directions along curves and the sizes of objects in the warped 3D visual space vary as an inverse function of the Euclidean distance r in the outside world. This causes a profound warping of 3D visual space affecting the representation of position, size, shape, curvature, outline, velocity and acceleration of objects and of the body in the outside world (see Section 3.1).

A smooth, one-to-one, onto, invertible map (i.e., a diffeomorphism) between the 3D outside world and the 3D warped visual space allows the actual size of an object to be related to its apparent size in warped visual space. Because the size-distance relationship and hence the metric $g\left(r,\theta ,\varphi \right)$ is derived in our proposal directly from vestibular, proprioceptive and visual afferent signals before perception is modified by top-down cognitive mechanisms of depth perception we refer to it as the *intrinsic geometry* of 3D visual space. The set of all cyclopean vectors $\left(r,\theta ,\varphi \right)$ span all the points in the 3D Euclidean environment. They also span the 3D warped visual space but the existence of the matrix $g\left(r,\theta ,\varphi \right)$ at each image point $\left(r,\theta ,\varphi \right)$ implies that the visual system anticipates the change in size of retinal images associated with change in Euclidean distance r in the outside world. If the distance r is incorrectly estimated, or the geometry of visual space is modified by top-down cognitive mechanisms based on learned experience, such as the expectation that faces are convex in shape or that rooms are rectangular and do not change size as we move about within them, then the one-to-one mapping between the outside world and the warped representation of it is lost and unrealistic visual perceptions (i.e., illusions) result.

We trust that the information in this section can serve to introduce Riemannian geometry as a beautiful and elegant branch of mathematics concerned with the calculus of nonlinear dynamical processes taking place in curved (or warped) manifolds. We believe it is the only existing mathematical framework able to handle the computational complexities underlying visually-guided movement. We now proceed to a full account of the theory.

## 3. Background

### 3.1. The Intrinsically-Warped Geometry of 3D Visual Space

Modern schematic models of the eye employ multiple refractory surfaces to emulate the full range of optical characteristics. However, as set out by Katz and Kruger ([46], Chapter 33), object-image relationships can be determined by simple calculations using the optics of the reduced model of the eye due to Listing. The geometry of the eye determines that the size of the retinal image varies in proportion to the angle subtended by the object at the nodal point of the eye. Or stated equivalently, the geometry of the eye determines that the size of the image changes in inverse proportion to the Euclidean distance between the object in the environment and the nodal point of the eye. Since the image on the retina is encoded by photoreceptors and signaled by retinal ganglion cells via the lateral geniculate nucleus to the primary visual cortex it follows that sizes of images on the retina are encoded within the visual afferent signals.

Proprioceptive and vestibular afferent signals combined with visual afferent signals allow binocular stereopsis, retinal-image disparity and focus control to be used to obtain an absolute measure of the Euclidean distance between the egocentre and points in the environment during each interval of fixed gaze. Thus information encoded within visual, proprioceptive and vestibular afferent signals is sufficient for the nervous system to compute the relationship between the size of the image on the retina and the Euclidean distance between the egocentre and points in the environment. This size-distance relationship can be represented by a Riemannian metric $g\left(r,\theta ,\varphi \right)$ at each image point $\left(r,\theta ,\varphi \right)$ in a representation of 3D visual space [6] in each partition of visuospatial memory.

Using Riemannian geometry to compute the effect that the size-distance relationship introduced by the eye has on the geometry of 3D visual space, we found it to have a profound influence [6]. The geometry of the computed 3D visual space corresponds to a Riemannian manifold with the egocentre at the origin and with a Riemannian metric that varies on the manifold in inverse proportion to the square of the Euclidean distance between the egocentre and the observed point in the environment as in Equation (1). Because this Riemannian geometry is computed directly from information encoded in afferent signals, before any possible modification by top-down cognitive estimates of depth, we refer to it as the *intrinsic geometry* of 3D visual space.

We showed that, with the head at a fixed place, the intrinsic Riemannian geometry of 3D visual space creates a conformal mapping between points in the outside world and their positions in the computed 3D warped visual space. Angles between coordinate lines in the outside world are preserved but the lengths and curvatures of lines are transformed. We found that every plane in the outside world passing through the egocentre is warped in 3D visual space in the same way. Concentric circles about the egocentre in the plane, radial lines emanating outward from the egocentre in the plane, and logarithmic spirals deviating inwards or outwards from circles in the plane in the outside world are represented by straight lines (known as geodesics, discussed in Section 3.7) in the intrinsically-warped 3D visual space. Radial lines (i.e., lines of gaze) are the only lines that are straight in both the outside world and in visual space but their lengths are foreshortened in visual space by the logarithm of their lengths in Euclidean space. Every other straight line joining any two points in the outside world is represented by a curved line in intrinsically-warped visual space. The arc lengths of segments on concentric circles about the egocentre in the outside world are represented in visual space by straight lines with lengths proportional to the angles between the radial lines spanning the segment. Thus the sizes of objects in the environment are represented by the angles they subtend at the egocentre or, equivalently, the represented size of an object decreases in inverse proportion to its Euclidean distance from the egocentre. In other words, objects appear to shrink in size without changing their shape as they recede without rotation along radial lines [6].

The intrinsic warping of the geometry of 3D visual space causes the represented position, size, outline, occlusions, curvature, velocity and acceleration of objects in visual space to change as a function of the position of the object in the outside world relative to the egocentre or, equivalently, their positions relative to the egocentre as the person moves about in the local environment. Clearly, this intrinsic warping of the geometry of 3D visual space attributable to the size-distance relationship of retinal images introduced by the anatomy and physiology of the eye has to be taken into account when making sensory-motor decisions about the coordinated movements (i.e., movement synergies) required to achieve visual goals.

### 3.2. The Need for Movement Synergies

We have addressed the need for synergies extensively in previous work [47,48]. To summarize and as introduced in Section 2.2, the human body has some 110 elemental movements (ball-park estimate) that can be controlled voluntarily independently of each other one at a time. In other words, there are no anatomical or physiological constraints that prevent the independent implementation of these 110 elemental movements. However, the nervous system has insufficient central processing resources to plan and execute independently-varying trajectories for all 110 elemental movements simultaneously. We contend that this limitation is overcome by the nervous system, at the same time solving the problem of redundancy in the neuro-musculo-skeletal system, by introducing task-dependent constraining relationships between groups of elemental movements so they move together in nonlinear dynamically-related ways. Each set of coupled elemental movements can then be controlled as a unit [47,48]. More than one set of coupled elemental movements can be controlled independently simultaneously but this number has to be small, say ≤10, because of limited central processing resources. To distinguish between biomechanical DOFs of the human body and the greatly reduced number of DOFs for elemental movement trajectories constrained by the nervous system to move together in a related fashion we use the term *control degrees of freedom* (CDOFs) to describe the latter. The number of CDOFs in the muscle synergy and in the descending alpha and gamma motor-command synergies is the same as the number of CDOFs in the movement synergy. Movement synergies greatly reduce the number of DOFs (i.e., the number of independently varying movements to be planned and executed in parallel in order to achieve a specified visual goal).

The duration for which a selected movement synergy can be maintained and the number of goal-oriented submovements that can be performed in sequence within it depend on the task. Consider the task of steering an automobile with both hands in a fixed grip on the steering wheel. This requires a coordination of the elemental movements of the shoulders, elbows, forearms and wrists of both arms to turn the wheel. Provided the grip on the wheel is not changed, this movement synergy can be maintained for hours despite that fact that many different visually-guided submovements are required within that synergy to steer the car along the road. On the other hand, a visually-guided task such as picking up and drinking from a glass requires the sequential selection of different movement synergies such as reach and grasp, pickup, transport with horizontal stability, place against lips, tilt and swallow, tilt, transport, place on table, and so on. The nervous system not only has to be able to generate differing sets of constraining relationships between elemental movements corresponding to different movement synergies but it also has to be able to switch quickly and smoothly from one synergy to the next in accordance with the actions chosen to achieve sequences of behavioural goals.

By coupling elemental movements together and controlling them as a unit the central workload involved in planning and executing task-dependent multi-joint coordinated movements is greatly reduced. Rather than planning and controlling trajectories for 110 elemental movements in parallel, a central response planning system has only to plan and control a small number *N* of independently varying coupled-movement trajectories. The price to be paid for this large reduction in demand on central processing resources is a requirement for the nervous system to have (i) neural circuitry able to generate task-dependent nonlinear dynamical constraining relationships between elemental movements and (ii) neural circuitry able to select and switch quickly and smoothly from a set of constraining relationships appropriate for one perceptual goal to another set of constraining relationships appropriate for the next goal in a changing sequence of goal-directed actions.

We have dealt with the first requirement previously [47,48] where we formally defined a set of task-dependent nonlinear dynamical constraining relationships between elemental movements to be a movement synergy, and referred to a neural circuit able to generate constraining relationships between descending drives to pools of alpha and gamma motor neurons of functional muscles as a *synergy generator*. We have also shown [47] that this involves both task-dependent synergy generators and wired-in (i.e., task-independent) synergy generators. The second requirement, provision of a neural basis for selecting and switching between synergies geared to behavioural goals, is addressed in the latter sections of this paper.

### 3.3. The Configuration Space of the Human Body Moving in 3D Euclidean Space

In Section 2.2 and in [5] we defined the 116-dimensional configuration space of the human body moving in the 3D Euclidean outside world to be the Cartesian product C=Θ×P×O of posture space Θ, place space *P* and orientation space *O*. Impossible postures and no-go places impose a boundary on the configuration space C. For example, there is an anatomical limitation on the range of each elemental movement and, in certain configurations, this range is further limited by parts of the body bumping into each other and/or bumping into objects in the environment. It is not possible to float into the air or to walk through a brick wall; in other words, possible configurations of the body are confined to the *configuration space with boundary* while impossible configurations are outside the configuration space with boundary. Thus whenever we mention configuration space (or configuration manifold) subsequently we mean only possible configurations within the configuration space with boundary.

Unstable postures leading to a fall can also be considered to be no-go postures outside the boundary. We have previously discussed [5] the issue of controlled falling and the inclusion of unstable configurations in functional movements such as walking jumping and running. We suggested that just as a person travelling in a bus or a train is able to shift his/her allocentric reference frame from the stationary outside world to $\left(X,Y,Z\right)$ coordinates attached to the inside of the moving vehicle, a high-diver doing a double somersault with half twist, for example, can shift his/her external reference frame to a point on his/her own body and thus plan somersault and twisting movements in free fall. We also find it reasonable to suppose that flexibility in changing the external reference frame may underlie ability to plan movements that include controlled falling such as walking, running and jumping.

### 3.4. The Mass-Inertia Matrix of the Body Changes with Configuration

The mass-inertia matrix J of the human body is a 110 × 110 symmetrical, positive-definite matrix. It is the kinetic-energy Riemannian metric (as in [49] and A.11) on the 110D posture manifold $\left(\Theta ,J\right)$. Given a learned model of the relationships between changes in the angles and positions of the elemental movements of the body and the associated changes in the lengths of functional muscles (see [47]), the mass-inertia loads on functional muscles can be computed from muscle-length and muscle-tension afferent signals. Because the support forces distributed across the body surface constrain movement (e.g., it is not possible to kick the leg you are standing on), and because the size and distribution of the support forces can change with configuration, it follows that the mass-inertia load about each elemental movement depends not only on the posture of the body but also on the place and orientation of the head. Think, for example, of the differences between standing and lying. In other words, the mass-inertia matrix $J\left(c\right)$ of the body can change as a function of the configuration c∈C=Θ×P×O.

This leads to a novel definition of the Riemannian metric on the configuration manifold C. Rather than the usual block-diagonal Riemannian-metric matrix on a product manifold we have a metric $J\left(c\right)$ on the posture manifold $\left(\Theta ,J\right)$ that is defined at every configuration c∈C in the configuration manifold C. For configurations outside the boundary in configuration space C we set $J\left(c\right)=\infty$ with a smooth transition in the vicinity of the boundary. As we will see this prevents the planning of minimum-effort movement trajectories from entering no-go places and impossible postures and from colliding with objects in the environment. We do not define a metric on the place space *P* or the orientation space *O* because these metrics are not required in our ensuing formulation.

### 3.5. Minimum Effort Movement Trajectories to Achieve Specified Visual Outcomes

Because there is a large number of elemental movements spanning the posture manifold $\left(\Theta ,J\right)$ it follows that many different coordinated movement trajectories in the posture manifold can achieve a specified visual outcome. We have proposed previously [5,47,48] that this problem of redundancy is overcome by selecting the unique coordinated movement trajectory that is able to achieve a specified goal with minimal demand by the muscles for metabolic energy. Since the amount of muscle force required to accelerate the body depends on the mass-inertia loads on the muscles, as described in Section 2.2, the fact that the mass-inertia matrix $J\left(c\right)$ of the body changes with configuration must be taken into account in computing a goal-directed minimum-effort coordinated movement trajectory.

However in computing a minimum-effort movement trajectory to achieve a specified *visual* outcome a further complication arises because, as addressed in Section 3.1, visual representations of objects in the intrinsically-warped 3D visual space change profoundly with changes in the place and orientation of the head in the environment. Thus the determination of trajectories planned to achieve specified visual goals must include precise specification of the position and orientation of the head as well as other required changes in posture. Apart from being transported about by a vehicle of some kind the only way a person can control the place and orientation of the head in the environment is by changing the posture of the body. But the relationship between body posture and the position and orientation of the head is ill-defined. The relationship changes depending on the configuration of the body. For example, changing the joint angles of the arms when doing push-ups changes the place and orientation of the head in the environment but if this is done when standing it does not do so. In general the relationship between changes in body posture and changes in the position and orientation of the head, and hence changes in visual images of objects in the environment, depends on the distribution of support forces on the body. This distribution can change with changes in configuration of the body.

How then is a minimum-energy trajectory to a visual goal achieved, given that the specification of the goal depends on the place and orientation of the head which in turn has an ill-defined dependence on the trajectory in posture-and-place space? This question is addressed in the section below. The answer plays an important role in the Riemannian theory of visually-guided movement synergies and will be referred to again in Section 5.

### 3.6. Movement Trajectories Confined to Local Regions in Configuration Space

**Figure 1:**
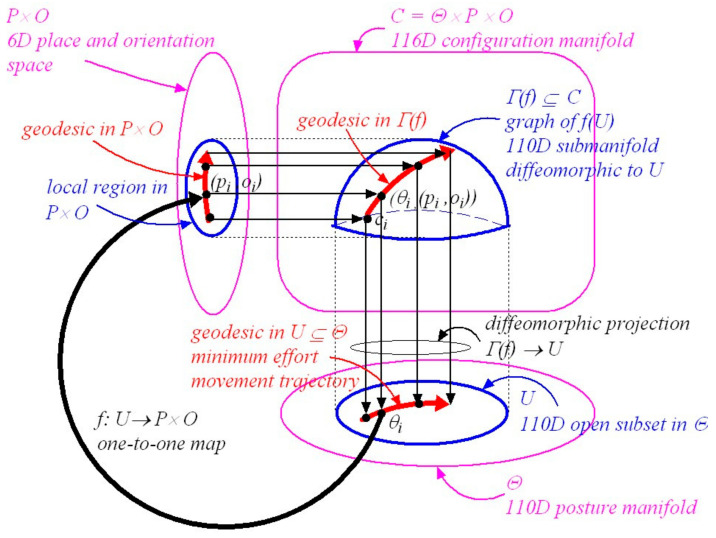
A schematic diagram illustrating the Riemannian theory of *graphs of submanifolds*. Θ designates the smooth 110D posture manifold spanned by the 110 elemental movements of the body. P×O designates the smooth 6D place-and-orientation manifold spanning the place and orientation space of the head in the 3D environment. U designates a neighbourhood in the posture manifold Θ about a given initial posture $\theta_{i}\in \Theta$ where there exists a fixed mapping f:U→P×O between the open subset U⊆Θ in posture space and the position and orientation of the head in a local region of P×O. The graph of the map f:U→P×O is designated by $\Gamma \left(f\right)$. $\Gamma \left(f\right)$ is a 110D submanifold embedded in the configuration manifold C=Θ×P×O that is diffeomorphic to the 110D open subset U⊆Θ in the posture manifold Θ. Different mappings f between posture and the place and orientation of the head are represented by different submanifolds $\Gamma \left(f\right)$.

To achieve a specified visual outcome a planned minimum-effort coordinated movement trajectory has to be confined to a local region in configuration space where there is a fixed smooth mapping between posture and the place and orientation of the head in the environment. Such a trajectory will be geodesic as explained in Section 3.7. Figure 1 illustrates this local control which can be described geometrically using Riemannian *graphs of submanifolds* theory, (A.12 and ([50], p.100)).

As depicted in Figure 1, $\Gamma \left(f\right)\subseteq C=\Theta \times P\times O$ denotes the graph of f:U→P×O. To restate what is shown in Figure 1, we can write:

(4) $$\Gamma \left(f\right)=\left\{\left(\theta ,\left(p,o\right)\right)\in C=\Theta \times P\times O:\theta \in U,\left(p,o\right)=f\left(\theta \right)\right\}$$

where $\Gamma \left(f\right)$ is a 110D submanifold embedded in C=Θ×P×O diffeomorphic to U⊆Θ, θ is a posture in the open subset U⊆Θ, $\left(p,o\right)$ is a place and orientation of the head in a local region of P×O, and f:U→P×O is a local smooth map between posture and the place and orientation of the head. Importantly, the map f can change with changes in the distribution of support forces acting on the body and hence with configuration. Some local changes in body posture confined to U⊆Θ leave the place and orientation of the head unchanged while other changes in U⊆Θ carry the place and orientation of the head along with them in a smooth one-to-one fashion. For example, moving the arms might leave the place and orientation of the head in the environment unchanged while bending at the waist might carry the place and orientation of the head in the environment along with it.

At every point $c=\left(\theta ,\left(p,o\right)\right)$ in the 110D submanifold $\Gamma \left(f\right)\subseteq C=\Theta \times P\times O$ there exists a 110 × 110 mass-inertia matrix $J\left(c\right)$. As explained in Section 3.7 this means that a minimum-effort (geodesic) natural free-motion trajectory determined by the mass-inertia characteristics of the body can be computed from anywhere to anywhere within the local 110D submanifold $\Gamma \left(f\right)$ (see A.12 and A.17). Any such computed minimum-effort geodesic movement trajectory in $\Gamma \left(f\right)$ maps in a smooth, one-to-one, onto, invertible (i.e., diffeomorphic) fashion onto a smooth minimum-effort movement geodesic trajectory in U⊆Θ. In turn this trajectory in U⊆Θ maps smoothly via the map f:U→P×O to a smooth minimum-effort geodesic trajectory in the place-and-orientation space P×O of the head in the environment. Thus minimum-effort movement trajectories that include precise control over the place and orientation of the head can be generated locally in a 110D submanifold $\Gamma \left(f\right)$ centred about a specified initial configuration $c_{i}$ derived from $f\left(\theta_{i}\right)$ in the configuration manifold C (i.e., in a local region of the configuration manifold C where there exists a fixed relationship between the local posture of the body and the local place and orientation of the head in the environment).

In essence, because the mass-inertia matrix $J\left(c\right)$ is the kinetic-energy Riemannian metric on the posture manifold Θ, all geodesics computed using $J\left(c\right)$ will be confined to the 110D posture manifold Θ. However, because the local map f:U→P×O between U⊆Θ and P×O is either a constant map or a smooth one-to-one map it follows that, locally at least, the geodesic in posture space either leaves the place and orientation of the head unchanged or it carries the place and orientation of the head along with it in a one-to-one fashion. Thus a geodesic trajectory in U⊆Θ maps diffeomorphically onto a geodesic in the submanifold $\Gamma \left(f\right)$. Locally at least, where the map f:U→P×O between posture and the place and orientation of the head is fixed, the geodesic in the posture manifold and the geodesic in the submanifold $\Gamma \left(f\right)$ are equivalent (i.e., they map diffeomorphically onto each other).

Similar local computations of minimum-effort movement trajectories apply in different local regions of configuration space C (i.e., in different embedded submanifolds $\Gamma \left(f\right)$) when the smooth fixed map f:U→P×O is different because of changes in the distribution of support forces acting on the body (e.g., standing, sitting, lying). It follows that minimum-effort movement trajectories to achieve specified visual outcomes have to be planned in appropriate local regions of the posture-and-place manifold where the movement trajectories carry the place and orientation of the head along with them in a one-to-one fashion. We will return to this in Section 5.

### 3.7. Geodesics in Configuration Space

Suppose the body is given an initial configuration $c_{i}\in C$ and an initial velocity $\dot{c}\in T_{c_{i}}\Gamma$, where $T_{c_{i}}\Gamma$ is the vector space tangent to the submanifold $\Gamma \left(f\right)$ at $c_{i}\in C$. Remember $T_{c_{i}}\Gamma$ is isomorphic to the tangent space $T_{c_{i}}\Theta$ as described in Section 3.6. Then, because the body has mass and rotational inertia (i.e., mass-inertia) about each elemental movement, in the absence of all external forces (including muscle forces) the body will follow a natural free motion trajectory $c\left(t\right)$ parameterized by time t in the configuration manifold C confined to the 110D submanifold $\Gamma \left(f\right)$. Natural free-motion trajectories are a property of all mechanical systems with mass-inertia as expressed by Newton’s first law, *a body will remain in a state of rest or uniform motion in a straight line unless acted on by an external force*. In other words, in the absence of all external forces (including muscle forces), the body will move along a trajectory $c\left(t\right)$ in the configuration manifold C in such a way as to conserve its kinetic energy (think of a body moving in a gravity-free environment). However, in the curved 110D submanifold $\Gamma \left(f\right)$ described above the mass-inertia matrix $J\left(c\right)$ changes with configuration and consequently the corresponding motion in Euclidean space has to accelerate and/or decelerate in order to preserve the kinetic energy of the body. In 3D Euclidean space the natural free-motion geodesic trajectory of the body is a curved accelerating and/or decelerating one. For a detailed description of a *geodesic trajectory generator* (GTG) able to generate geodesic trajectories from anywhere to anywhere in the configuration manifold C see ([5] Section 5) and ([6] Section 4).

Our proposal that humans use the mass-inertial properties of the body efficiently when planning goal-directed movements is not without experimental support. In ([5] Section 2.2) we reviewed studies showing experimentally that rotations of the eyes, hand and limb movements, swinging movements of the leg during walking, movements of the head-eye system, multi-joint arm movements, and reaching movements involving coordinated rotations of the head, clavicles, shoulders, elbows, wrists and bending of the vertebral column all correspond to geodesic trajectories of a Riemannian manifold defined by a coordinate system based on the DOFs of the movement.

In considering movement of the body there always exist (i) visco-elastic forces attributable to connective tissue and to the tension-length-velocity characteristics of muscles, (ii) posture-dependent gravitational forces and torques acting about each elemental movement and (iii) configuration-dependent support forces distributed over the surface of the body that constrain movement. To hold the body in a fixed equilibrium posture or to follow a geodesic movement trajectory these ever-present but changing external forces have to be balanced by muscle forces. Nevertheless, since mass-inertial loads on muscles and gravitational torques dominate other forces, the unique geodesic pathway connecting a specified initial configuration to a specified final configuration (i.e., c($t_{i}$) to c($t_{f}$)) remains the most energy-efficient pathway despite the existence of other ever-present visco-elastic external forces [5]. Gravitational forces are conservative forces so the same amount of energy is required to overcome gravity in moving between c($t_{i}$) and c($t_{f}$) no matter which pathway between them is chosen.

## 4. Posture-and-Place-Encoded Visual Images

### 4.1. Image Points, Image-Point Vectors and Visual Space

In this section we extend the previous *place-encoded* theory of visuospatial memory [6] to a *posture-and-place-encoded* theory (outlined in Section 2.4). Each posture-and-place partition of visuospatial memory consists of an association memory network that associates image-point vectors with their corresponding image points as seen from each posture and place during each fixed-gaze interval. Over time, through visual scanning, as the person moves about in the environment, each posture-and-place associated partition of visuospatial memory accumulates an encoded visual image of all the objects in the environment and of the body in that environment as seen from that place and with the body in that posture. We refer to these accumulated images of the environment and of the body in that environment as posture-and-place-encoded visual images. Every partition of visuospatial memory is spanned by the cyclopean coordinates $\left(r,\theta ,\varphi \right)$ that parameterize 3D visual space. Orientation of the head is absorbed into the cyclopean coordinates $\left(r,\theta ,\varphi \right)$. Thus each partition of visuospatial memory provides an internal egocentric representation of the 3D environment and of the body in that environment as seen when the head is at that place and the body is in that posture.

### 4.2. Visual Scanning of Objects and of the Body

To relate vision with action the nervous system has not only to encode 3D visual images of objects in the environment along with the way they appear to change from place to place (i.e., perceived optical flow), but it must also encode 3D visual images of the body in that environment along with the way those images change with posture.

When moving in a local 3D Euclidean environment a person is able to visually scan not only objects in the environment and their surrounds but also the visible surfaces of his/her own body. This is how a person becomes familiar with a local environment and with visual and proprioceptive images of the body in that environment. We propose that “snapshots” of the encoded retinal-hyperfield images for each gaze point are processed and accumulated in visuospatial memory just as described in [6] Section 3 except that now we partition that memory according to both posture and place.

Because our focus here is on visually-guided movement we restrict attention to those movements of the body that can be sensed visually. While all 110 elemental movements of the body spanning posture space Θ can be sensed proprioceptively some cannot be sensed visually regardless of which posture the body assumes. For example, it is not possible to see one’s own head or the angles of one’s eyes in the head (reflections in a mirror do not count). Again as a ball-park estimate, 73 can be detected visually, these being the elemental movements (joint-angles) of the shoulders, arms, hands, fingers, trunk, hips, legs, feet and toes. We therefore introduce the notation Ψ to represent the 73D *visible-posture space* spanned by the 73 joint-angles that can be sensed both proprioceptively and visually. Accordingly a point $\psi_{i},$ i=1,2,⋯,∞, represents one of the infinite number of possible postures in the 73D visible-posture space Ψ. Because the orientation of the head in the environment and the angles of the eyes in the head are excluded from Ψ these can be varied while a visually-perceived posture $\psi_{i}$ in Ψ is held constant. It is possible therefore, over time, within the possible configuration space of the body, to accumulate in visuospatial memory encoded visual images of all the points on the surface of the body that can be seen when the body is in all the possible visible postures $\psi_{i},$ i=1,2,⋯,∞, in Ψ⊆Θ.

Analogous to our previous proposal [6] of a visuospatial memory with partitions $\left(G_{p_{i}},g\right)$ associated with a place $p_{i}\in P$ we can now construct a visuospatial memory with partitions of posture $\psi_{i}\in \Psi$ and place $p_{i}\in P$ rather than just place. Each $\left(G_{\left(\psi_{i},p_{i}\right)},g\right)$ in each partition of visuospatial memory is associated with a posture $\psi_{i}\in \Psi$ of the body and a place $p_{i}\in P$ of the head as given by the location of the egocentre in the 3D environment. All encoded retinal images associated with different points of gaze made whenever the body is in posture $\psi_{i}$ and the head is at place $p_{i}$ are accumulated for detail see ([6] Section 3.1) in the memory partition $\left(G_{\left(\psi_{i},p_{i}\right)},g\right)$. Encoded retinal-hyperfield images are associated with the cyclopean coordinates $\left(r,\theta ,\varphi \right)$ for points in the 3D Euclidean environment projecting onto retinal hyperfields during intervals of fixed gaze when the body is in posture $\psi_{i}$ and the head is at place $p_{i}$. These retinal images include objects that are moving independently in the environment but such images are transient and do not accumulate over time in visuospatial memory. The symbol g within each memory partition represents the Riemannian metric on the egocentric 3D visual space $\left(G_{\left(\psi_{i},p_{i}\right)},g\right)$ that quantifies the intrinsically-warped Riemannian geometry of visual space introduced by the size of the image on the retina varying in inverse proportion to the Euclidean distance to the object in the environment [6]. This warping is determined by the anatomy and physiology of the eye and is the same in every posture-and-place partition $\left(G_{\left(\psi_{i},p_{i}\right)},g\right)$ of visuospatial memory.

As previewed in Section 2.7 if the place $p_{i}$ of the head is held fixed while the posture $\psi_{i}$ is changed, only the visual image points and image-point vectors associated with points on the surface of the body change while visual image points and image-point vectors associated with points on the surfaces of objects fixed in the environment remain unchanged. Conversely, if the posture $\psi_{i}$ of the body is held fixed while the place $p_{i}$ of the head in the environment is changed (think of the mannequin analogy), only the visual image points and image-point vectors associated with points on the surfaces of objects in the environment change. Of course, changes in the orientation of the head relative to the external reference frame (*X*,*Y*,*Z*) will change the direction $\left(\theta ,\varphi \right)$ of the cyclopean gaze coordinates $\left(r,\theta ,\varphi \right)$ measured relative to the external reference frame (*X*,*Y*,*Z*) for all image points on the surface of the body. But this is easily taken into account by simply adding the orientation of the head relative to (*X*,*Y*,*Z*) encoded within the hippocampus to the gaze coordinates for image points on the surface of the body measured relative to the egocentre. Remember, the orientation of the head is absorbed into the cyclopean coordinates $\left(r,\theta ,\varphi \right)$.

With posture $\psi_{i}$ held fixed, image points and image-point vectors associated with different points on the surface of the body are located at different depths in the egocentric visual space $\left(G_{\left(\psi_{i},p_{i}\right)},g\right)$. The images change with depth in the same way as do image points and image-point vectors associated with points on the surfaces of objects in the environment. In other words, the body is sensed visually in the same egocentric visual space $\left(G_{\left(\psi_{i},p_{i}\right)},g\right)$ with the same intrinsically-warped geometry as are environmental objects. However, as the posture $\psi_{i}$ of the body changes, the cyclopean coordinates $\left(r,\theta ,\varphi \right)$ in each $\left(G_{\left(\psi_{i},p_{i}\right)},g\right)$ of each image point on the surface of the body change. Consequently, because of the intrinsically-warped geometry of the 3D egocentric visual space $\left(G_{\left(\psi_{i},p_{i}\right)},g\right)$, the visual representation of the outline and of the position, size, curvature and orientation of visual patches on the surface of the body in visual space all change in a systematic way with changes in their cyclopean coordinates $\left(r,\theta ,\varphi \right)$. For example, think of the change in the visual image of the hand as it is moved from being close to the face to a position with the arm outstretched.

We hold that it is biologically feasible to develop a partitioned visuospatial memory based on posture-and-place-encoded visual images of the local environment and of the body in that environment as seen with the body in every possible posture $\psi_{i}$ and from every possible place $p_{i}$ of the egocentre in that environment. Again, as introduced in Section 2.7, we suggest that this partitioning involves two streams of visual processing, one for posture-encoded images of the body associated with activity in the somatosensory cortex encoding each posture proprioceptively, the other for place-encoded images of objects in the environment associated with activity in the hippocampus encoding the place of the head. We propose that both streams come together in control of visually-guided movement and, as will be taken up in Section 8.7, this is independent of conscious perception.

### 4.3. The Geometric Structure of Posture-and-Place Encoding

**Figure 2:**
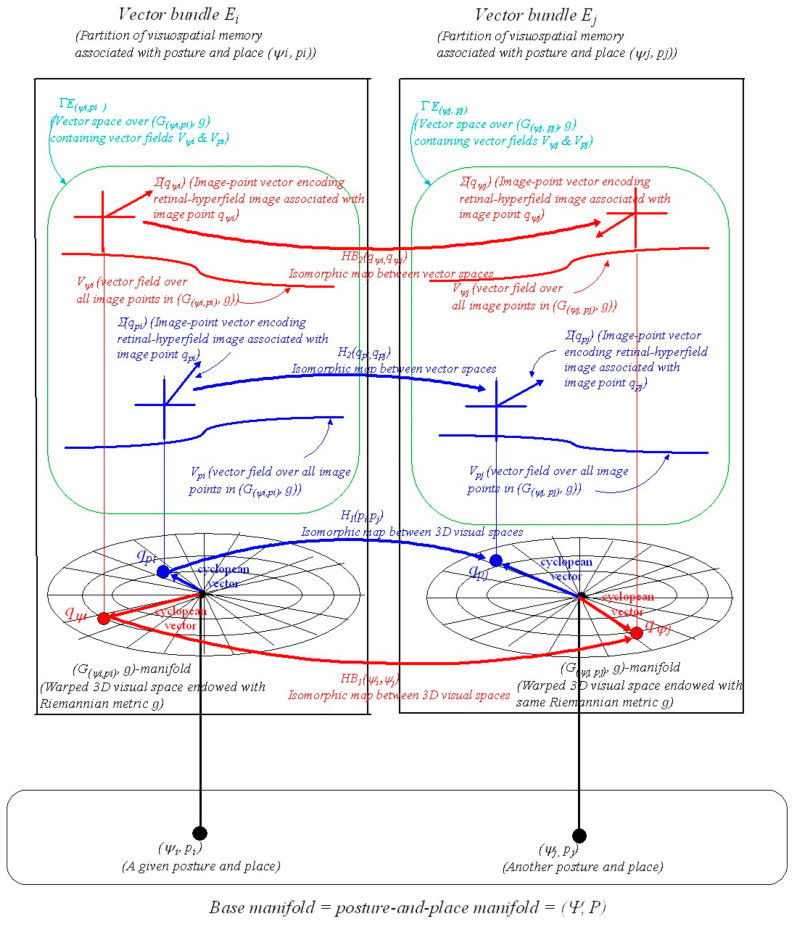
A schematic diagram of visuospatial memory illustrating the geometric fibre-bundle structure of place-and-posture-encoded visual images of objects in the environment and of the body in that environment as seen from each posture and place $\left(\psi_{i},p_{i}\right)$. Posture is coloured red and place is coloured blue. At each point $\left(\psi_{i},p_{i}\right)$ there exists a fibre containing a vector bundle $E_{i}$ corresponding to a partition of visuospatial memory. Only two such vector bundles, $E_{i}$ and $E_{j}$, are illustrated. $\left(G_{\left(\psi_{i},p_{i}\right)},g\right)$ represents the 3D perceived visual space encoded within each vector bundle. $H_{1}\left(p_{i},p_{j}\right)$, $H_{2}\left(q_{p_{i}},q_{p_{j}}\right)$, $H_{B1}\left(\psi_{i},\psi_{j}\right)$, and $H_{B2}\left(q_{\psi_{i}},q_{\psi_{j}}\right)$ represent adaptively-tuned and wired-in maps (vector bundle morphisms) between each and every partition of visuospatial memory. When a change occurs in the place $p_{i}$ of the head and/or the posture $\psi_{i}$ of the body, these vector bundle morphisms map the corresponding changes in the retinal-hyperfield image points q (cyclopean vector) and image-point vectors $\Sigma \left(q\right)$ for fixed points in the environment and/or on the surface of the body. Further description follows in the text.

We now extend our earlier place-encoded structure ([6] Section 7, Figures 10 and 11) to include posture-and-place-encoded 3D visual images not only of objects in the environment seen in the correct perspective from every place in the environment but also of the body in every possible visually-perceivable posture $\psi_{i}$ in that environment. As in the previous paper, we introduce the concept of a *vector bundle*, a common structure in differential geometry (see A.13). In the present context a vector bundle is formed by collecting together all the image-point vectors (i.e., encodings of retinal-hyperfield images) at all the image points (i.e., cyclopean coordinates of the retinal-hyperfield images) in the manifold $\left(G_{\left(\psi_{i},p_{i}\right)},g\right)$ (i.e., representation of 3D curved visual space, seen from a given posture and place $\left(\psi_{i},p_{i}\right),$ spanned by cyclopean coordinates $q=\left(r,\theta ,\varphi \right)$ and endowed with Riemannian metric g). This combined object represents a partition of visuospatial memory. We illustrate this in Figure 2.

In Figure 2 a point $\left(\psi_{i},p_{i}\right)$ in the 76D posture-and-place base-manifold (Ψ,P) represents the posture $\psi_{i}$ of the body in the 73D posture space Ψ and the place $p_{i}$ of the head in the 3D Euclidean environment. At each posture-and-place $\left(\psi_{i},p_{i}\right)$ in (Ψ,P) there exists a fibre (i.e., an association) containing a vector bundle $E_{i}$. This type of geometrical structure is known in differential geometry as a *fibre-bundle*. Each $E_{i}$ corresponds to a partition of visuospatial memory. Each partition of visuospatial memory corresponds to a gaze-based association memory network in which each image-point vector (i.e., each encoded retinal-hyperfield image seen from $\left(\psi_{i},p_{i}\right)$) is associated with its image point (i.e., its cyclopean coordinates $q=\left(r,\theta ,\varphi \right)$ for each retinal-hyperfield image seen from $\left(\psi_{i},p_{i}\right)$). Encoded visual images of the body and of objects in the environment are accumulated in each partition of visuospatial memory over time through visual scanning when the body passes through posture $\psi_{i}$ and the head passes through place $p_{i}$. This gradually acquired encoding is represented geometrically in Figure 2 by the 3D gaze-based base manifold $\left(G_{\left(\psi_{i},p_{i}\right)},g\right)$ spanned by cyclopean coordinates (or cyclopean vectors) $q=\left(r,\theta ,\varphi \right)$ with the egocentre at the origin.

The metric g and hence the intrinsic curved geometry of each gaze-based manifold $\left(G_{\left(\psi_{i},p_{i}\right)},g\right)$ is the same in each memory partition. However, the image points $q=\left(r,\theta ,\varphi \right)$ and the encoded image-point vectors $\Sigma \left(q\right)$ for each point on the visible surface of the body and for each point on the visible surface of a fixed object in the environment change from one vector bundle $E_{i}$ to another $E_{j}$ because of changes in occlusions and changes in perspective associated with changes in posture and place. Since image points $q_{\psi_{i}}=\left(r,\theta ,\varphi \right)$ for points on the surface of the body and image points $q_{p_{i}}=\left(r,\theta ,\varphi \right)$ for points on the surfaces of objects in the environment are always located at different points in each egocentric 3D visual space $\left(G_{\left(\psi_{i},p_{i}\right)},g\right)$ they can be processed and stored in each $\left(G_{\left(\psi_{i},p_{i}\right)},g\right)$ separately.

With a change in posture from $\psi_{i}$ to $\psi_{j}$ the image point $q_{\psi_{i}}$ and its encoded image-point vector $\Sigma \left(q_{\psi_{i}}\right)$ for a single visible body point represented in vector bundle $E_{i}$ change to $q_{\psi_{j}}$ and $\Sigma \left(q_{\psi_{j}}\right)$ for the same visible body point represented in vector bundle $E_{j}$ (i.e., for the same point on the surface of the body but seen with the body in a different posture). Similarly, with a change in place from $p_{i}$ to $p_{j}$ the image point $q_{p_{i}}$ and its encoded image-point vector $\Sigma \left(q_{p_{i}}\right)$ for a single point on a fixed object represented in vector bundle $E_{i}$ change to $q_{\psi_{j}}$ and $\Sigma \left(q_{\psi_{j}}\right)$ for the same point on the same fixed object represented in vector bundle $E_{j}$ but seen from a different place (Figure 2). Some image points that can be seen in vector bundle $E_{i}$ are occluded from view in vector bundle $E_{j}$ and vice versa. Some image points are occluded in both $E_{i}$ and $E_{j}$ but can be seen from other places and/or postures. Some points on the surface of the body such as those on the head and some points on the surfaces of objects such as those on surfaces permanently pushed together cannot be seen from any place and/or posture and so are not encoded visually.

While Figure 2 illustrates a change in the position of the image point and image-point vector for single points it is to be understood that through visual scanning the same encoding occurs for all points seen on the surface of the body and on the surfaces of objects as a person moves about in the local environment. In each $\left(G_{\left(\psi_{i},p_{i}\right)},g\right)$ the collection of image points $q_{\psi_{i}}$ encode the visible surface of the body seen when in posture $\psi_{i}$ and the collection of image points $q_{p_{i}}$ encode the visible surfaces of all objects fixed in the environment seen from place $p_{i}$. The union of all the image-point vectors $\Sigma \left(q_{\psi_{i}}\right)$ in vector bundle $E_{i}$ over all the image points $q_{\psi_{i}}$ forms a vector field $V_{\psi_{i}\;}$ over $\left(G_{\left(\psi_{i},p_{i}\right)},g\right)$ encoding the images of all visible body surfaces that can be seen when in posture $\psi_{i}$. The union of all the image-point vectors $\Sigma \left(q_{p_{i}}\right)$ in vector bundle $E_{i}$ over all the image points $q_{p_{i}}$ forms a vector field $V_{p_{i}}$ over $\left(G_{\left(\psi_{i},p_{i}\right)},g\right)$ encoding images of all the visible objects in the environment seen from place $p_{i}$ (Figure 2). This is simply the mathematical expression of the idea that through visual scanning a person can build up in memory an image of the entire local environment and of the body in that environment as seen from each fixed posture and place.

As shown in Figure 2 the vector bundle $E_{i}$ consisting of base manifold $\left(G_{\left(\psi_{i},p_{i}\right)},g\right)$ together with a vector space $\Gamma E_{\left(\psi_{i},p_{i}\right)}$ containing vector fields $V_{\psi_{i}}$ and $V_{p_{i}}$ over $\left(G_{\left(\psi_{i},p_{i}\right)},g\right)$ can be mapped to another vector bundle $E_{j}$. A map between two vector bundles is known as a *vector bundle morphism* (see A.14). This too is a common mathematical structure in differential geometry. The maps [$H_{B1}\left(\psi_{i},\psi_{j}\right)$, $H_{B2}\left(q_{\psi_{i}},q_{\psi_{j}}\right)$] and [$H_{1}\left(p_{i},p_{j}\right)$, $H_{2}\left(q_{p_{i}},q_{p_{j}}\right)$] in Figure 2 are vector bundle morphisms for posture-encoded images of the body (subscripts B1 and B2 for body) and place-encoded images of objects in the environment, respectively. Vector bundle morphisms between all the image points and image-point vectors in each and every partition of visuospatial memory can be formed adaptively to transform image points and image-point vectors between each and every vector bundle $\left\{E_{i}\right\}$ (i.e., between each and every partition of visuospatial memory).

We have shown previously ([6] Section 7.5) that when an image point $q_{p_{i}}$ on the surface of an object can be seen in vector bundle $E_{i}$ but $q_{p_{j}}$ is occluded from view in vector bundle $E_{j}$ the transformation [$H_{1}\left(p_{i},p_{j}\right)$, $H_{2}\left(q_{p_{i}},q_{p_{j}}\right)$] still applies. Likewise we show below that when an image point $q_{\psi_{i}}$ on the surface of the body can be seen in vector bundle $E_{i}$ but $q_{\psi_{j}}$ is occluded from view in vector bundle $E_{j}$ the transformation [$H_{B1}\left(\psi_{i},\psi_{j}\right)$, $H_{B2}\left(q_{\psi_{i}},q_{\psi_{j}}\right)$] still applies. Thus when vector bundle morphisms are applied for all the image points and image-point vectors between each and every vector bundle (i.e., between each and every memory partition) the resulting transformations form 3D images of the environment and of the body in that environment seen in the correct perspective from every posture and place with occlusions filled in. More on occlusions can be found in our description ([6] Section 8.7) of the layer 1, layer 2 and layer 3 structure of visuospatial memory.

### 4.4. Redundancy in Posture-to-Vision Maps

By definition the vector bundle morphisms [$H_{B1}\left(\psi_{i},\psi_{j}\right)$, $H_{B2}\left(q_{\psi_{i}},q_{\psi_{j}}\right)$] transform the image point and the image-point vector for a single point on the surface of the body between vector bundle $E_{i}$ and vector bundle $E_{j}$ associated with posture $\psi_{i}$ and $\psi_{j}$, respectively. While the vector bundle morphism $H_{B1}\left(\psi_{i},\psi_{j}\right)$ is an isomorphic map between 3D visual spaces (Figure 2), the map $H_{B1}\left(\psi_{i},\psi_{j}\;\right)$ itself depends on postures $\psi_{i}$ and $\psi_{j}$ that are both vectors in a 73D visible-posture space Ψ. Thus the change in position in 3D visual space of a single image point on the surface of the body associated with a change in posture from $\psi_{i}\in \Psi$ to $\psi_{j}\in \Psi$ involves a transformation between a 73D visible-posture space Ψ sensed proprioceptively and a map $H_{B1}\left(\psi_{i},\psi_{j}\right)$ between 3D visual spaces $\left(G_{\left(\psi_{i},p_{i}\right)},g\right)$ and $\left(G_{\left(\psi_{j},p_{j}\right)},g\right)$. In other words, the proprioceptive-to-vision maps $\Psi \to H_{B1}\left(\psi_{i},\psi_{j}\right)$ for single image points on the surface of the body are redundant. Many different visible postures $\psi_{i}\in$ Ψ in proprioceptive space can locate a given point on the surface of the body at the same point $q_{p_{i}}=\left(r,\theta ,\varphi \right)$ in 3D visual space $\left(G_{\left(\psi_{i},p_{i}\right)},g\right)$ (think of all the visible postures the body can assume with the head and the tip of a finger both at fixed positions). Nevertheless, in order to remove occlusions, we require vector bundle morphisms [$H_{B1}\left(\psi_{i},\psi_{j}\right)$, $H_{B2}\left(q_{\psi_{i}},q_{\psi_{j}}\right)$] (as in Figure 2) for individual image points on the surface of the body in all visible postures $\left\{\psi_{i}\right\},\left\{\psi_{j}\right\}$ ∈ Ψ. These maps can serve to anticipate changes in the visual image of the body in the environment associated with changes in posture experienced proprioceptively. They can also play a role in learning to match one’s own body posture with that of another when learning movement skills through imitation.

Our ability to plan and execute goal-directed movements such as reaching to catch a ball without actually having to look at the present location of the hand (i.e., the relevant body part) implies that, despite redundancy in posture-to-vision maps for individual image points, the nervous system *does* possess one-to-one proprioception-to-vision and vision-to-proprioception maps. Indeed, it is easy to convince oneself that one-to-one maps are possible by noting the one-to-one relation between changes in body posture sensed proprioceptively with changes in the visual reflection of the whole body in a mirror. Moreover we have observed informally but repeatedly during the extensive tracking experimentation conducted in our own laboratory that hiding the hand and joystick from view during a visual pursuit task has no effect on performance. In fact subjects choose to look only at the display and do not pay any visual attention to the hand even if it is possible to do so. The nervous system seems to “know” the position of the hand and thus of the joystick in space without having to look directly at them. It would seem that perceiving the posture of the body proprioceptively is sufficient to locate where parts of the body are in 3D visual space. The question is, how does the nervous system achieve proprioception-to-vision and vision-to-proprioception transformations given the high level of redundancy in the warped proprioception-to-vision maps $\Psi \to H_{B1}\left(\psi_{i},\psi_{j}\right)$?

### 4.5. Overcoming Redundancy in Posture-to-Vision Maps

The task is to determine the cyclopean coordinates $\left(r,\theta ,\varphi \right)$ of a sufficient number of visible image points on body segments to specify the position and orientation of each segment and so give a unique posture. We showed previously ([6] Sections 2.9 and 2.10) how binocular triangulation and retinal image disparity provide sufficient information for the cyclopean coordinates $\left(r,\theta ,\varphi \right)$ to be computed for both foveal and peripheral hyperfield images across the retinas of both left and right eyes during a fixed gaze interval. As set out in ([6] Section 6.3), computing the partial derivatives (or covariant derivatives) of depth r as a function of θ and φ at each point on a visible surface seen from a fixed posture and place is part of the computation required to compute the shape (curvature) of the surface (i.e., to “see” the shape of the surface). The visible surface of the body in each posture $\psi_{i}\in$ Ψ is a connected space containing an infinite number of image points. There is no shortage of visible image points on each body segment that can be tracked across changes in posture. According to our Riemannian formulation, all the image points visible on the surface of the body in each posture $\psi_{i}$ are stored into an appropriate partition $\left(G_{\left(\psi_{i},p_{i}\right)},g\right)$ of visuospatial memory. For a particular posture $\psi_{i}\in$ Ψ there exists a unique visual image in the 3D visual manifold $\left(G_{\left(\psi_{i},p_{i}\right)},g\right)$ of all the parts of the body that can be seen with the body in that posture. That image is represented by the vector field $V_{\psi_{i}}$ in Figure 2.

As posture changes, the cyclopean coordinates r, θ and φ of individual image points on the body surface change. But they do not change independently of each other! For example, all the image points on any one body segment are constrained to move so that the actual Euclidean distance between them remains fixed. Similarly, changes in a proximal elemental movement cause related movement changes of image points on all the rigid-body segments distal to that elemental movement. Certainly, as the posture changes from $\psi_{i}$ to $\psi_{j}$ in Ψ sensed proprioceptively, image points on the body surface change their cyclopean coordinates $\left(r,\theta ,\varphi \right)$ between the 3D egocentric visual spaces $\left(G_{\left(\psi_{i},p_{i}\right)},g\right)$ and $\left(G_{\left(\psi_{j},p_{j}\right)},g\right)$ depicted in Figure 2. Some image points visible in $\left(G_{\left(\psi_{i},p_{i}\right)},g\right)$ become occluded in $\left(G_{\left(\psi_{j},p_{j}\right)},g\right)$ but there are always multiple image points visible on each body segment in each posture. The fact that the cyclopean coordinates r, θ and φ for image points on the surface of the body do not change independently of each other with changes in posture can be used to overcome redundancy associated with modeling $H_{B1}\left(\psi_{i},\psi_{j}\right)$ for individual image points. In other words the posture-and-place-encoded images of all the visible parts of the body accumulated in visuospatial memory contain sufficient information to enable that redundancy to be removed.

Let us illustrate with the simplified example of a 7-DOF arm (3 rotations at the shoulder, 1 rotation at the elbow, 1 rotation of the forearm, and 2 rotations at the wrist) moving in 3D Euclidean space. How can the 7D proprioceptive space of the arm be mapped into a 3D visual space in a one-to-one, invertible fashion? This cannot be done if we consider the position of only a single point on the arm. It can be done, however, if we consider the positions of a grid of image points on the surface of the arm with multiple points on each segment. Take the following illustration. Three numbers are required to specify the position of a single point in 3D space. Six numbers are required to specify the positions of two points in 3D space. But alternatively we can think of two points moving independently in 3D space as equivalent to a single point moving in 6D space. Thus for the 7-DOF arm, if we orthogonalize the r, θ and φ cyclopean coordinates in 3D Euclidean space for multiple surface points on the entire arm as the arm moves from posture to posture in its 7D proprioceptive space, we will obtain seven independently changing orthogonal coordinates. Conceptually these seven orthogonal coordinates describing the position of the arm in 3D Euclidean space are equivalent to a single point moving in a 7D visual space. Thus a non-redundant, one-to-one, invertible map can be constructed between the 7D proprioceptive space and the equivalent 7D visual space. We can think of this as a map between the posture of the arm in joint-angle space and a visual image of the entire arm (multiple image points) in 3D visual space. Redundancy has been removed!

We return now to the case of the entire body moving in a 73D visible-posture space Ψ. To remove redundancy from the relationships between the r, θ and φ cyclopean coordinates of multiple visible image points on the surface of the body we use a novel procedure based on the nonlinear Gram-Schmidt orthogonalization process given in [47]. We have detailed the verification of this procedure for a variety of nonlinear dynamical relationships using data with various non-Gaussian amplitude probability distributions and non-white power-spectral distributions ([47] Sections 4.2 and 7.3). The method is implemented via a network of nonlinear adaptive filters and it is the adaptive parameters in these filters that tune the Gram-Schmidt algorithm. We have long held that such networks are ubiquitous throughout sensory and motor systems of the nervous system [47,51,52,53,54]. It can also be noted that whatever the posture the body $\psi_{i}\in$ Ψ, the relationships between the r, θ and φ coordinates for image points on its surface are nonlinear but *algebraic* rather than dynamic. These are therefore relatively easy to model adaptively using the nonlinear Gram-Schmidt algorithm.

In every posture $\psi_{i}$ some of the image points will be occluded from view but there will always exist many other image points on each body segment that are visible and can be tracked across a subset of changing postures. Within the nonlinear Gram-Schmidt orthogonalizing algorithm the relationships between the coordinates r, θ and φ of pairs of image points are estimated only for those postures in which both image points are visible. The parameters describing the relationship are held unchanged whenever one or other of the image points is occluded from view. The orthogonalizing algorithm is not disrupted by such sections of missing data (discontinuities are smoothed out by the modeling algorithm). There is always a sufficiently large number of visible image points on each body segment to remove redundancy and to estimate the position and orientation of each segment. With a sufficiently long sequence of changing postures $\psi_{i}$ ∈ Ψ included in the adaptive modeling process, relationships between most of the image points on the surface of the body are included (i.e., most but not all pairs of image points can be seen together in one or other subset of postures).

The Gram-Schmidt algorithm generates a set of 73 orthogonalized signals $\left(Q_{1},\cdots ,Q_{73}\right)$ that uniquely encodes the positions in 3D visual space of all the image points on the surface of the body with redundancy removed. We can think of this equivalently as a point moving in a 73D visual space. Then there exists a one-to-one, onto, invertible, smooth map between the orthogonalized representation $\left(Q_{1},\cdots ,Q_{73}\right)$ of the non-redundant position of the body in the equivalent 73D visual space and the posture of the body in the 73D visible-posture space Ψ sensed proprioceptively. This provides an invertible vision-to-proprioceptive and proprioceptive-to-vision map (independent of place) for every joint-angle of the body spanning the visible-posture space Ψ. Once the parameters of the adaptive filters in the nonlinear Gram-Schmidt algorithm have tuned, the time required to transform vision into proprioception or vice versa is negligible, no more than the time taken for neural signals to flow through the orthogonalizing network.

**Figure 3:**
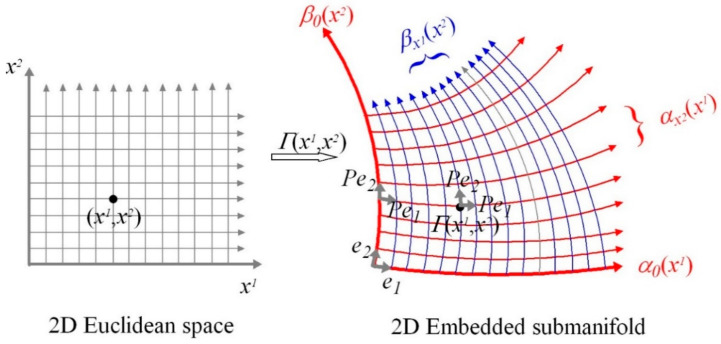
A schematic diagram illustrating the generation of a 2D geodesic submanifold $\Gamma \left(x^{1},x^{2}\right)$ corresponding to a selected two-CDOF minimum-effort movement synergy embedded in the 116D configuration manifold $\left(C,J\right)$ of the body moving in a local 3D environment. The coordinate axes $\alpha_{0}\left(x^{1}\right)$ and $\beta_{0}\left(x^{2}\right)$ and all the horizontal coordinate grid lines $\alpha_{x^{2}}\left(x^{1}\right)$ are geodesics (coloured red) in the posture-and-place manifold (Ψ,P) while all the vertical coordinate grid lines $\beta_{x^{1}}\left(x^{2}\right)$ are not geodesics (coloured blue). Detailed description in text.

An important fact about the adaptive nonlinear Gram-Schmidt orthogonalization algorithm is that it works equally well in the reverse direction without needing to re-compute nonlinear relationships. This is done simply by changing certain minus signs in the orthogonalizing network to plus signs as shown in ([47] Section 4.2 and Figure 3). The orthogonalized visual signals $\left(Q_{1},\cdots ,Q_{73}\right)$ encoding the position of the body in 3D visual space with redundancy removed are easily transformed back through the tuned deorthogonalizing network of nonlinear adaptive filters into the set of interrelated cyclopean coordinates $\left(r,\theta ,\varphi \right)$ for the positions of individual image points on the surface of the body associated with any posture $\psi_{i}\in$ Ψ sensed proprioceptively. This allows the transformation $H_{B1}\left(\psi_{i},\psi_{j}\;\right)$ of individual image points $q_{\psi_{i}}$ in vector bundle $E_{i}$ in Figure 2 to be transformed into the image points $q_{\psi_{j}}$ in $E_{j}$ even when $q_{\psi_{j}}$ is occluded from view in vector bundle $E_{j}$.

## 5. The Geometry of Synergistic Movement to a Visual Goal

### 5.1. The Visual Task Space and Minimum-Effort Synergies

We use the term *visual task space* to mean all the visually-perceived images of (i) objects in the environment and (ii) parts of the body in that environment that are relevant to the performance of the task. All such visually-perceived images depend on the posture of the body and/or the place of the head in the environment. Consequently, we propose that the visual goal for a movement synergy is specified by a collage of posture-and-place-encoded visual images of the body (or parts of the body) in the environment sufficient to span the task space for that synergy. In the case of reaching and grasping a glass for example, a posture-and-place-encoded image of the body in its initial configuration $c_{i}\in C$ and a posture-and-place-encoded image of the hand grasping the glass in its final configuration are sufficient to span the visual task space. If the reaching movement has to avoid other objects in the environment (such as a tabletop when the hand is initially located beneath it) then additional posture-and-place-encoded images are required to specify the *via points*. This will increase the number of CDOFs in the movement synergy compatible with the collage of visual images but that number will remain small, say ≤ 10, because of limitations in central processing resources. In the case of a task such as writing with a pencil on a sheet of paper, a collage of posture-and-place-encoded visual images of the hand holding the pencil in the required extreme *x*- and *y*-positions on the paper (and perhaps in the *z*-direction if the pencil lifts off the paper) is sufficient to specify the task space.

If the task involves objects moving independently in the environment, such as walking without bumping into an oncoming pedestrian, the specification of the task space will require not only visual images of the body in the environment from visuospatial memory but also immediate information about the independently moving object. This will be acquired in short-term memory by appropriately directing the gaze to track the moving object. As we have shown previously, time series of visual observations can be used for stochastic prediction, in this case to estimate future positions of the moving object [55,56]. These predictions can be used to form future visual goals spanning the required movement synergy in both space and time. Indeed in general it can be argued that forming visual goals for movement synergies will depend on a varying mixture of predictions based on immediate visual information and posture-and-place-encoded visual images stored in visuospatial memory.

Many different criteria can be involved in specifying task goals for submovements. Some might minimize demand for metabolic energy, some might achieve goals in minimum time, some might maximize accuracy of the final configuration, some might maximize a performance criterion such as the height of a jump, some might require accuracy in both space and time, some might maximize comfort, some might require the movement to look smooth and elegant, while some might require weighted combinations of all of these, among many other options. It is also possible to introduce a tradeoff between the number of cascaded movement synergies required to achieve a task goal and the number of CDOFs in each synergy.

While a variety of criteria are possible for individual goal-oriented submovements, we propose that underlying these in general the nervous system forms movement synergies to achieve task goals in the most muscle-energy efficient manner [5,57,58]. For example, a person might decide to move from A to B by crawling. This might not be the most energy-efficient way of getting from A to B but nevertheless, once decided, the nervous system will find the best coordinated joint-angle trajectories and the best patterns of muscle activations to achieve those crawling movements with minimum demand for muscular effort. Running as fast as possible to catch a departing bus may not be the most energy-efficient way to reach the bus stop but again, once decided, the nervous system will ensure that the joint-angle trajectories and muscle activation patterns chosen are the most energy-efficient that are compatible with running fast. This is what we mean by minimum-effort movement synergies compatible with task goals.

### 5.2. Visually-Guided Movements Planned in a Local Region of the Configuration Space

For a person moving about in a local environment, say a room, the configuration of the body is different when at different locations in the room even though the posture $\theta_{i}\in$ Θ might be the same (e.g., standing). The configuration is also different when the posture differs (e.g., leaning on the elbows, sitting in a chair, lying on a couch, and so on) and it is different again if the orientation changes (e.g., standing facing the door vs facing the window). In other words, all 116 variables spanning the configuration manifold C=Θ×P×O are required to specify uniquely the configuration of the body in the local environment. In regard to selecting synergies to accomplish visuomotor goals it is necessary to consider all 116 dimensions of the configuration manifold C=Θ×P×O and not just the 110D posture space Θ. As introduced in Section 3.6, specified visual outcomes can only be generated in local regions of the configuration manifold. This is because the relationship between the posture of the body and the place and orientation of the head changes from one region of configuration space to another due to change in the distribution of support forces acting on the body and also because the mass-inertia matrix $J\left(c\right)$ of the body changes with configuration and not just posture. To obtain precise control of the place and orientation of the head in the environment, as well as of other required changes in posture, geodesic trajectories have to be generated in the local submanifold $\Gamma \left(f\right)$ where there is a fixed relation between posture and place. Remember $\Gamma \left(f\right)$ maps diffeomorphically onto a local open subset U in posture space Θ as illustrated in Figure 1.

To generate a minimum-effort one-CDOF movement synergy to achieve a specified visual goal it is necessary to specify an initial configuration $c_{i}\in C$ in the configuration manifold C and an initial direction-of-movement vector $e_{1}$ in the space $T_{c_{i}}\Gamma$ tangent to the submanifold $\Gamma \left(f\right)$ at the initial configuration $c_{i}$. As shown below, this ensures that the geodesic movement trajectory is confined to the required local 110D submanifold $\Gamma \left(f\right)$ in configuration space where there is a fixed map between posture and the place (and orientation) of the head in the environment. If the initial direction-of-movement vector $e_{1}$ is confined to the subspace of $T_{c_{i}}\Gamma$ isomorphic to $T_{c_{i}}\Psi$ then the resulting minimum-effort movement trajectory is still contained in the required submanifold in configuration space because the visible-posture space Ψ is a subspace of Θ. The problem becomes one of selecting the initial configuration $c_{i}$ and the initial direction-of-movement vector $e_{1}$ for the geodesic trajectory to achieve the place-and-posture-encoded visual image that specifies the final goal. As mentioned in Section 2.6 and fully developed in Section 7 our proposal is that people circumvent this complex two-point boundary-value problem by using reinforcement learning to select the appropriate $\left(c_{i},e_{1}\right)$.

### 5.3. A Simplified Description of Riemannian Graph Theory

In the following sections we refer repeatedly to minimum-effort submanifolds in the posture-and-place manifold (Ψ,P). This is a shorthand way of describing the geometric construction based on Riemannian graph theory of the submanifold $\Gamma \left(f\right)$ in configuration space C (Section 3.6 & Figure 1). Such control is important if the minimum-effort geodesic movement trajectory is to achieve a specified visual outcome. For example, suppose a glass is sufficiently far away that one has to lean forward to grasp it. This requires a change in the place and orientation of the head as part of the coordinated reaching movement. Since the encoded visual images of objects in the environment change with a change in the place and orientation of the head it follows that the leaning-forward movement is encoded within the specified collage of visual images spanning the required visual task space. Thus the minimum effort movement synergy (geodesic submanifold) has to be selected so that the place and orientation of the head together with the position of the hand are appropriately coordinated to achieve the specified visual reach and grasp outcome with minimal muscular effort. This defines what is meant by our subsequent reference to a *local minimum-effort trajectory in the posture-and-place manifold* (Ψ,P), or to a local minimum-effort *submanifold* in the case of a movement synergy with more than one CDOF.

Given an initial configuration $c_{i}\in C$ associated with a distribution of support forces acting on the body that constrain movement it is possible to generate, consistent with those constraints, a geodesic trajectory in posture space Ψ that moves the posture outside the open subset U⊆Ψ and consequently outside the submanifold $\Gamma \left(f\right)$ described in Section 3.6. Lifting the head from a pillow or standing up from a chair, for example, changes the distribution of support forces acting on the body and changes the mapping between body posture and the place and orientation of the head in the environment. Thus movements that transition from one local region $\Gamma \left(f\right)$ to another in the configuration manifold C are possible.

### 5.4. Constructing a Local Minimum-Effort Movement Synergy Compatible with a Specified Visual Goal

Given a visual goal specified by a collage of posture-and-place-encoded visual images defining a visual task space with more than one dimension, the most energy-efficient movement synergy compatible with that visual goal corresponds to a unique geodesic submanifold spanned by geodesic trajectories embedded in the posture-and-place manifold (Ψ,P) centred about a specified initial configuration $c_{i}\in C$.

An *N*-dimensional submanifold will be centred about the specified initial configuration $c_{i}\in C$ and spanned by *N* geodesic coordinate axes given by a set of specified orthonormal, initial velocity vectors $\left(e_{1},\cdots ,e_{N}\right)$ in the 73D tangent space $T_{c_{i}}\Psi$. While each unit metric-speed geodesic trajectory has zero metric-acceleration (i.e., zero covariant derivative of velocity) and is a straight line in warped posture space Ψ, it is a curved accelerating trajectory in the outside Euclidean world. Only an initial configuration $c_{i}$ and an initial unit velocity vector e is needed to generate each unit speed geodesic in the manifold (A.17). These *N* geodesic trajectories emanating from $c_{i}\in C$ in the orthonormal directions specified by $\left(e_{1},\cdots ,e_{N}\right)$ correspond to natural free-motion trajectories of the body attributable to its mass-inertia characteristics confined to the 73D submanifold $\Gamma \left(f\right)$ embedded in configuration space passing through the initial configuration $c_{i}\in C$. Every point $\left(\psi_{i},p_{i}\right)$ in this submanifold is a point in the posture-and-place manifold (Ψ,P) and is associated with a posture-and-place 3D visual image of the environment and of the body in that environment as seen from that posture and place. *The submanifold must be such that the posture-and-place-encoded visual images associated with each and every posture and place* $\left(\psi_{i},p_{i}\right)$ within the submanifold include the collage of visual images specifying the visual task space. We now address its construction.

Unfortunately, Riemannian geometry provides no guarantee that there will always exist a *totally geodesic* low-dimensional submanifold centred about a specified initial configuration $c_{i}\in C$ in a 116D configuration manifold C compatible with a specified collage of place-and-posture-encoded visual images. A totally geodesic submanifold requires all the coordinate axes and coordinate grid lines to be geodesics and is a true minimum-effort submanifold [6]. In that previous paper we described a procedure using a combination of parallel translation (see A.17) and Jacobi lifts (see Appendix B) able to test whether or not a totally geodesic submanifold exists. We also described a procedure for constructing a totally geodesic submanifold when one does exist. If a totally geodesic submanifold compatible with the specified visual goal does *not* exist, there nevertheless will be a unique low-dimensional submanifold, centred about the specified initial configuration $c_{i}\in C$ compatible with the specified collage of visual images, that closely approximates a totally geodesic submanifold. *This does always exist and can always be constructed*. As shown below, the cost of this approximation is that some coordinate grid lines (e.g., the vertical coordinate grid lines in Figure 3) deviate slightly from a natural geodesic free-motion of the body. Consequently additional muscle effort is required for movement along these coordinate grid lines. However, if the submanifold is confined to a sufficiently small region about the specified initial configuration $c_{i}\in C$ then the deviation is small and little additional muscle effort is required. Moreover, within the realm of all the physically-possible movement synergies compatible with the specified visual goal, the approximation described gives the synergy amongst them that requires the minimum effort.

The procedure for constructing such a submanifold is based on a procedure in Riemannian geometry known as *variation through geodesics* (see A.18 for definition and Appendix B for detail). This still depends on generating geodesics by means of the GTG ([5] Sections 4 and 5). We now outline the application of the procedure in constructing submanifolds with one-, two-, and *N*-CDOFs, respectively in the 76D posture-and-place manifold (Ψ,P).

#### 5.4.1. One-Dimensional Submanifold

Given initial vectors $\left(c_{i},e_{1}\right)$ specifying a selected one-CDOF movement synergy the corresponding 1D submanifold embedded in the configuration manifold $\left(C,J\right)$ is constructed just as set out in [5] Section 5.2. The one-dimensional submanifold is simply the geodesic trajectory $\alpha_{0}\left(x^{1}\right)$ in the posture-and-place manifold (Ψ,P) in C passing through the point $c_{i}\in C$ generated with initial position and velocity for the array of double integrators in the GTG set to $\left(c_{i},e_{1}\right)$. The initial velocity vector $e_{1}$ is in the tangent space $T_{c_{i}}\Psi$ (remember $T_{c_{i}}\Psi$ is isomorphic to $\;\;T_{c_{i}}\Gamma )$. Submovements confined to such a 1D submanifold (i.e., confined to the geodesic pathway) induce a set of nonlinear dynamical constraining relationships (including any constant relationships) between the 73 visible elemental movements of the body. Thus the geodesic pathway provides a geometric representation of a selected one-CDOF movement synergy. For a full account of similar procedures see ([5] Sections 4.4, 5.1, 5.2 and 8).

#### 5.4.2. Two-Dimensional Submanifold

Given a set of initial vectors $\left(c_{i},e_{1},e_{2}\right)$ specifying a selected two-CDOF movement synergy the corresponding 2D variation through geodesics $\Gamma \left(x^{1},x^{2}\right)$ embedded in C is illustrated in Figure 3. Since the configuration space C is a 116-dimensional space it follows that the 2D variation through geodesics $\Gamma \left(x^{1},x^{2}\right)$ illustrated in Figure 3 is a 2D submanifold embedded in the high dimensional configuration space C. In general, this prevents the coordinate grid lines spanning the 2D submanifold from being *totally geodesic* and leads to a compromise in which only the horizontal coordinate grid lines are geodesics while the vertical coordinate grid lines are not geodesics.

The embedded submanifold $\Gamma \left(x^{1},x^{2}\right)$ is constructed as follows: The initial vectors $\left(c_{i},e_{1}\right)$ and $\left(c_{i},e_{2}\right)$ are used as initial conditions in the GTG to generate unit speed geodesic coordinate axes $\alpha_{0}\left(x^{1}\right)$ and $\beta_{0}\left(x^{2}\right)$ in the posture-and-place manifold (Ψ,P). The initial orthonormal vectors $\left(e_{1},e_{2}\right)$ are confined to the tangent space $T_{c_{i}}\Psi$. Using *parallel translations* (see A.17) of $Pe_{1}$ and $Pe_{2}$ along $\beta_{0}\left(x^{2}\right)$ to obtain the initial positions and velocities for the GTG, all the horizontal geodesic coordinate grid lines $\alpha_{x^{2}}\left(x^{1}\right)$ can also be generated by the GTG. All the horizontal unit-speed geodesics $\alpha_{x^{2}}\left(x^{1}\right)$ are parameterized by metric-distance $x^{1}$ (i.e., arc length) along the horizontal geodesic curves. The vertical unit-speed geodesic corresponding to the vertical geodesic coordinate axis $\beta_{0}\left(x^{2}\right)$ is parameterized by metric-distance $x^{2}$ (i.e., arc length) along the vertical geodesic coordinate axis. Arc lengths $x^{1}$ and $x^{2}$ along the geodesic coordinate axes $\alpha_{0}\left(x^{1}\right)$ and $\beta_{0}\left(x^{2}\right)$, respectively, are measured from the initial configuration $c_{i}\in C$. As described below, temporal planning of submovement trajectories within the submanifold $\Gamma \left(x^{1},x^{2}\right)$ requires only specification of minimum metric-acceleration trajectories $x^{1}\left(t\right)$ and $x^{2}\left(t\right)$ along the geodesic coordinate axes $\alpha_{0}\left(x^{1}\right)$ and $\beta_{0}\left(x^{2}\right)$, so the synergy greatly reduces the demand for central processing resources even though multiple coupled joint-angle changes can be involved. Variations in metric-distances $x^{1}$ and $x^{2}$ along the geodesic coordinate axes $\alpha_{0}\left(x^{1}\right)$ and $\beta_{0}\left(x^{2}\right)$ correspond to the two CDOFs of the movement synergy.

As an approximation to a totally geodesic 2D submanifold embedded in the configuration manifold C we construct vertical coordinate grid lines $\beta_{x^{1}}\left(x^{2}\right)$ not as geodesic trajectories as required for a totally geodesic submanifold but simply by connecting points that are equal metric-distances (arc lengths) $x^{1}$ along the horizontal geodesic coordinate grid lines $\alpha_{x^{2}}\left(x^{1}\right)$, as shown in Figure 3. Remember, every point $\Gamma \left(x^{1},x^{2}\right)$ in the submanifold is a point $\left(\psi_{i},p_{i}\right)$ in the posture-and-place manifold (Ψ,P) and is associated with a posture-and-place 3D visual image of the environment and of the body in that environment as seen from that posture and place. Movement confined to such a 2D submanifold $\Gamma \left(x^{1},x^{2}\right)$ embedded in the high dimensional posture-and-place manifold (Ψ,P) implies a set of nonlinear dynamical constraining relationships with two CDOFs between the elemental movements of the body. Thus in keeping with our definition of movement synergy, this provides a geometric representation of a selected two-CDOF movement synergy. Providing the arc-length $x^{1}$ along the horizontal geodesic coordinate axis $\alpha_{0}\left(x^{1}\right)$ is kept small the deviation from a totally geodesic submanifold will be minimal.

To provide an intuitive illustration of a 2D variation through geodesics, if we were to consider this construction procedure applied to the surface of the planet Earth (idealized as a sphere) we would obtain a set of geodesic longitude lines (analogous to the horizontal geodesic coordinate grid lines) and a set of latitude lines (analogous to the vertical coordinate grid lines). Longitude lines on a spherical Earth are geodesics (great circle pathways) but latitude lines are not geodesics just as the horizontal coordinate grid lines $\alpha_{x^{2}}\left(x^{1}\right)$ in a variation through geodesics are geodesics but the vertical coordinate grid lines $\beta_{x^{1}}\left(x^{2}\right)$ are not geodesics. However, the equator on the spherical Earth is a geodesic just as the vertical coordinate axis $\beta_{0}\left(x^{2}\right)$ in the variation through geodesics is a geodesic. Indeed it is this conceptualization of Earth that originally gave us the name “geodesic”.

#### 5.4.3. *N*-Dimensional Submanifold

Given a set of initial vectors $\left(c_{i},e_{1},\cdots ,e_{N}\right)$ with orthonormal vectors $\left(e_{1},\cdots ,e_{N}\right)$ confined to the tangent space $T_{c_{i}}\Psi$ the same procedure as above can be iterated to construct an *N*-dimensional submanifold in (Ψ,P) using variation through geodesics. Firstly the initial vectors $\left(c_{i},e_{1}\right)$, $\left(c_{i},e_{2}\right)$, ⋯, $\left(c_{i},e_{N}\right)$ are used as initial conditions in the GTG to generate *N* geodesic coordinate axes. Then, using parallel translation and the GTG, multiple copies of the *N*-1 dimensional submanifold are generated inductively and the coordinate grid points are connected together to give non-geodesic coordinate grid lines.

#### 5.4.4. The Two-Point Boundary Value Problem

Importantly, each of the geodesic coordinate axes in the procedures above is uniquely specified by a pair of initial condition vectors $\left(c_{i},e\right)$ (see A.17). But each geodesic trajectory has to connect the specified initial configuration $c_{i}$ with a specified final posture-and-place-encoded visual image for that CDOF. Thus the task is to find the initial condition vectors $\left(c_{i},e\right)$ for each geodesic coordinate axis compatible with the specified posture-and-place-encoded visual image for that CDOF. This is a nonlinear, multi-dimensional, two-point boundary value problem that is difficult to solve. Our proposed solution involves reinforcement learning explained in Section 7.

### 5.5. Temporal Response Planning in a Submanifold

In detailing the construction of a local minimum-effort submanifold the sections above provide an account of the *spatial response planning* of visually-guided movement. We have previously written extensively on the temporal response planning of movement tasks [47,59,60,61] so we provide only a brief description here.

A submovement with a specified duration $\left(t_{f}-t_{i}\right)$ confined to a selected movement synergy (i.e., selected submanifold) can be generated by independently planning a minimum metric-acceleration (i.e., minimum covariant derivative of the velocity vector) trajectory parameterized by time t between specified initial and final positions and velocities at times $t_{i}$ and $t_{f}$, respectively, predicted ahead in time along each of the geodesic coordinate axes spanning the submanifold. We use metric-acceleration rather than metric-jerk trajectories because the latter require position, velocity and acceleration to be predicted ahead, introducing excessive prediction-error variance. Besides, metric-acceleration takes into account the local curvature of the submanifold in configuration space C and a minimum metric-acceleration trajectory along the geodesic pathway corresponds to a minimum muscle-force trajectory. Each trajectory is generated by a parallel-processing neural circuit referred to as an *optimum trajectory generator* (OTG) first described in [51] and further detailed in [59,60]. To take the curvature of Riemannian manifolds into account the original OTG requires only the simple modification of replacing ordinary derivatives with covariant (metric) derivatives (see A.15). Like the original, each modified OTG operates with a fixed time interval to read in high-level sensory information, generate a required minimum metric-acceleration trajectory along each specified geodesic coordinate axis, and write this into working memory ready for execution in real time.

The trajectory along each geodesic coordinate axis corresponds to a single independent performance variable (i.e., CDOF) in a multi-CDOF task and can be specified and generated independently. When these independently planned minimum metric-acceleration trajectories along each of the geodesic-coordinate-axis pathways are executed together the result is a minimum-effort movement trajectory within the selected submanifold (i.e., within the selected movement synergy). As described elsewhere [47,54] the feedforward–feedback motor control system that executes these planned submovements is a multivariable adaptive optimal control system capable of controlling a small number N≤10 CDOFs in parallel. This movement controller has a key role in the implementation of synergies but will not be discussed further in the current context of synergy selection.

### 5.6. Synergy Submanifolds Are Confined to Local Regions in Configuration Space

In a small enough region in the posture manifold Ψ about the specified initial configuration $c_{i}\in C$ a geodesic trajectory in posture space Ψ either leaves the place of the head in the environment unchanged or carries it along in a one-to-one fashion. In other words, as outlined in Section 3.6 and Section 5.3, it generates a geodesic trajectory in a local 73D submanifold $\Gamma \left(f\right)$ of the configuration manifold C diffeomorphic to an open subset U⊆Ψ in the posture space Ψ. As the distance $x^{1}$ of a non-geodesic vertical coordinate grid line (e.g., $\beta_{x^{1}}\left(x^{2}\right)$ in Figure 3) from the vertical geodesic coordinate axis (i.e., $\beta_{0}\left(x^{2}\right)$ in Figure 3) increases, deviation of the non-geodesic vertical coordinate grid line from a geodesic increases. The amount of deviation depends on the sectional curvatures of the submanifold in the vicinity of the initial configuration $c_{i}\in C$ which in turn depends on the double covariant derivatives of the metric $J\left(c\right)$ in that region of C (see equations for $\Gamma_{jk}^{i}$ and $R_{ijkl}$ in Appendix B). Thus for configurations where the mass-inertia matrix $J\left(c\right)$ changes rapidly (accelerates) with configuration, such as foot landing and foot take-off in a walking cycle, the submanifold has to be small (local) to maintain a good approximation to a totally geodesic submanifold.

We have already seen, however, that the submanifold must be confined to a local region about the initial configuration $c_{i}\in C$ to maintain a fixed smooth one-to-one relationship between posture and the place and orientation of the head in the environment. We have also seen that the submanifold must be confined to a small neighbourhood of the initial configuration to ensure that the horizontal geodesic coordinate grid lines do not converge and cross each other, as can happen if C is locally positively curved (see Appendix B). In all cases, the greater the local curvature of C the smaller the submanifold has to be to approximate a totally geodesic submanifold. This can be seen in Figure 3 where greater local deviation of the horizontal geodesic grid lines $\alpha_{x^{2}}\left(x^{1}\right)$ corresponds to greater local curvature of the configuration manifold. Thus in general, because of nonlinearities, geodesic submanifolds corresponding to minimum-effort movement synergies have to be confined to small neighbourhoods U⊆Ψ of the specified initial configuration $c_{i}\in C$ in posture space Ψ. This implies that, because of nonlinearities, frequent switching between movement synergies is required as a person moves about in a local environment. To maintain smooth movement despite frequent switches of synergy the submanifold for each synergy must intersect with the submanifold of the next synergy in the sequence. We do not explore this further here but these intersections of geodesic submanifolds in (Ψ,P) determine the laws of transition between movement synergies and provide a basis for future work.

## 6. Proprioceptive-to-Vision and Vision-to-Proprioceptive Maps

### 6.1. The Synergy Submanifold in Visual Space

As described in Section 5 and illustrated in Figure 2 every point $\left(\psi_{i},p_{i}\right)$ in the subspace (Ψ,P) of the configuration manifold C=Θ×P×O corresponding to a given posture and place is associated with a partition $\left(G_{\left(\psi_{i},p_{i}\right)},g\right)$ of visuospatial memory. Consequently, every point in the selected geodesic submanifold embedded in the posture-and-place manifold (Ψ,P) is associated with a posture-and-place-encoded visual image of the environment and of the body in that environment as seen from that posture and place. In other words, the selected synergy submanifold embedded in (Ψ,P) can be mapped into a submanifold of posture-and-place-encoded visual images of the environment and of the body in that environment confined to points $\left(\psi_{i},p_{i}\right)$ within the selected submanifold (i.e., selected movement synergy).

Using the simplified example of a two-DOF arm we will now illustrate that, despite the nonlinear warping of both posture space and visual space, the position of the hand in the 3D outside world as the arm moves along geodesic pathways in joint-angle space can be mapped in a one-to-one, onto, invertible, smooth fashion into the warped 3D visual space.

### 6.2. Simulation of a Proprioceptive-to-Visual Map for a Two-DOF Arm

**Figure 4:**
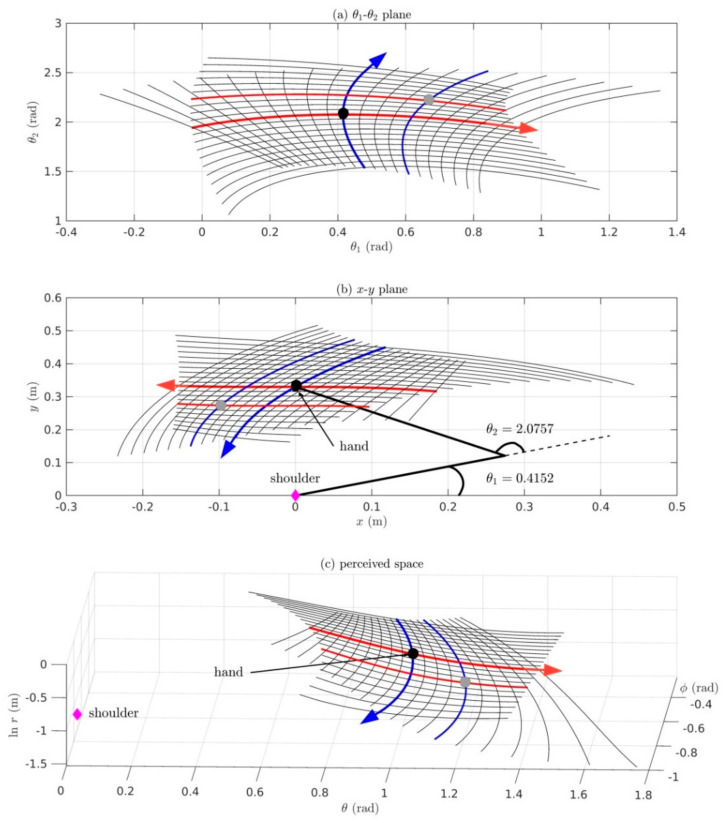
Results of MATLAB/Simulink simulation of a two-DOF arm moving in the horizontal plane through the shoulder depicting the transformation of geodesic trajectories in the 2D curved proprioceptive joint-angle space into the 3D curved visual space $\left(G,g\right)$. (**a**) shows a totally geodesic grid in joint-angle space (*θ*_1_–*θ*_2_) of the two-DOF arm moving along natural free-motion geodesic trajectories in the horizontal plane attributable to its mass-inertia characteristics. (**b**) shows the corresponding (*x-y*)-positions of the hand in the Euclidean (*x-y*) horizontal plane for corresponding points along the geodesic grid lines in (**a**). These were computed trigonometrically using Equation (4). The line drawing in Figure (**b**) illustrates the *θ*_1_ and *θ*_2_ angles of the arm when the hand is located at the centre of the grid. (**c**) shows the corresponding grid of visually-perceived positions of the hand in the 3D warped visual space $\left(G,g\right)$ spanned by the cyclopean coordinates $\left(\ln\;r,\theta ,\varphi \right)$ as described in the text. Equivalent example trajectories in (**a**–**c**) are indicated by lines of similar colour and thickness. Arrows on these lines indicate the directions in which joint angles *θ*_1_ and *θ*_2_ are increasing.

**Figure 5:**
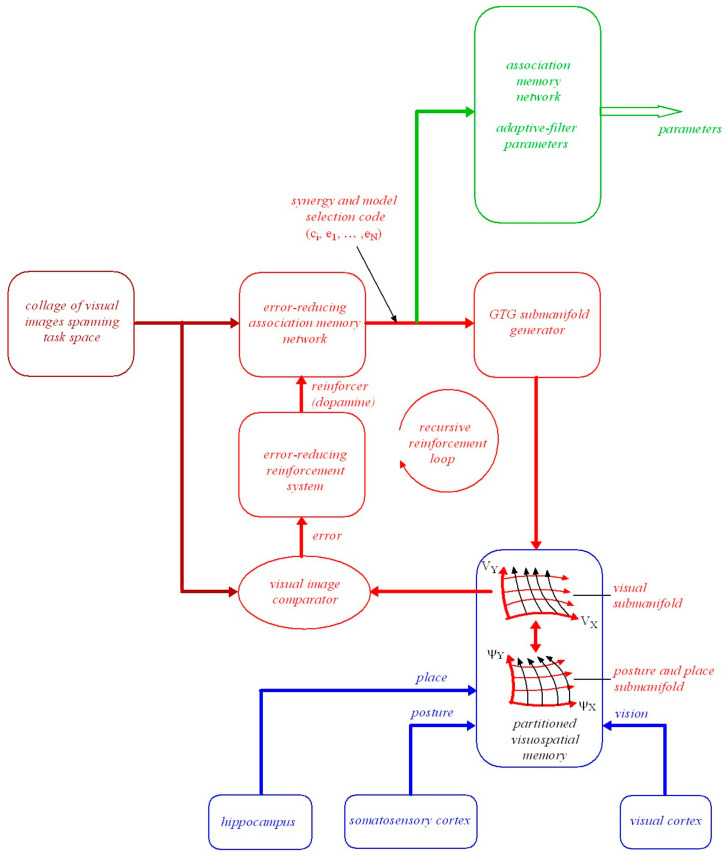
A block diagram illustrating response planning processes involved in selecting a movement synergy compatible with a specified visual goal. The central feature is the recursive reinforcement loop coloured in red. A block-by-block description of the figure follows in the text.

In this section we use a MATLAB/Simulink simulator to compute the nonlinear mapping between the 2D proprioceptive warped geodesic submanifold and the position of the hand in the intrinsically-warped 3D visual space for a two-DOF arm moving in the horizontal plane. It is important to appreciate that a 2D submanifold embedded in a 2D space can be totally geodesic. Thus, unlike the 2D submanifold embedded in a high dimensional space illustrated in Figure 3, for the two-DOF arm both the horizontal and vertical coordinate grid lines as well as the horizontal and vertical coordinate axes in the simulation shown in Figure 4a are geodesics and the submanifold is totally geodesic. The simulation builds on the earlier simulation ([5] Section 8, Figures 4–6) where we computed the two-CDOF totally geodesic proprioceptive submanifold for the two-DOF arm moving in the horizontal plane at shoulder height. The purpose of the new simulation is to demonstrate that, despite the intrinsic warping of 3D visual space, there is a one-to-one, onto invertible map between the position of the hand in the 3D Euclidean outside world and the perceived position of the hand in 3D warped visual space. This remains true regardless of the dimension of the proprioceptive space.

The 2D proprioceptive submanifold for the arm is spanned by the shoulder angle *θ*_1_ and the elbow angle *θ*_2_. The velocity vector at (*θ*_1_, *θ*_2_) is ($\dot{\theta }$_1_, $\dot{\theta }$_2_). The mass-inertia matrix (i.e., kinetic-energy metric) is:

(5) $$J\left(\theta_{2}\right)\;=\left[\begin{array}{cc} J_{11}\left(\theta_{2}\right) & J_{12}\left(\theta_{2}\right)\\ J_{12}\left(\theta_{2}\right) & J_{22}\left(\theta_{2}\right) \end{array}\right]=\left[\begin{array}{cc} I_{1}+I_{3}+2I_{5}\cos\theta_{2} & I_{3}+I_{5}\cos\theta_{2}\\ I_{3}+I_{5}\cos\theta_{2} & I_{3} \end{array}\right],$$

where constants are: $I_{1}=I_{1,x}+m_{1}a_{1}^{2}+m_{2}l_{1}^{2}$; $I_{3}=I_{2,x}+m_{2}a_{2}^{2}$; $I_{5}=m_{2}l_{1}a_{2}$; $I_{1,x}=$ moment of inertia of upper arm; $I_{2,x}=$ moment of inertia of forearm; $l_{1}=$ length of upper arm; $l_{2}=$ length of forearm; $m_{1}=$ mass of upper arm; $m_{2}=$ mass of forearm; $a_{1}=$ distance to centre of mass of upper arm; $a_{2}=$ distance to centre of mass of forearm. In the simulation these parameters are set to $l_{1}=$ 0.30 m, $l_{2}=$ 0.345 m, $m_{1}=$ 2.52 kg, $m_{2}=$ 2.07 kg, $a_{1}=$ 0.142 m, $a_{2}=$ 0.225 m, $I_{1,x}=$ 0.019 kg m^2^, and $I_{2,x}=$ 0.021 kg m^2^.

The inertial constants in Equation (5) are derived assuming the arm to be rigidly supported at the shoulder girdle. The mass-inertia matrix $J\left(\theta_{2}\right)$ changes as a function of elbow angle *θ*_2_ and the proprioceptive submanifold ((*θ*_1_, *θ*_2_), $J\left(\theta_{2}\right)$) is a Riemannian manifold with $J\left(\theta_{2}\right)$ equal to the kinetic-energy metric tensor. Using these data we derived expressions for the acceleration geodesic spray vector $f_{2}$:

(6) $$\left.\begin{array}{cc} f_{2}^{1}\left(\theta_{2},\dot{\theta_{1}},\dot{\theta_{2}}\right) & =\frac{J_{12}}{det\left[J\right]}m_{2}l_{1}a_{2}\sin\left(\theta_{2}\right){\left(\dot{\theta_{1}}\right)}^{2}+\frac{J_{22}}{det\left[J\right]}2m_{2}l_{1}a_{2}\sin\left(\theta_{2}\right)\left(\dot{\theta_{1}}\dot{\theta_{2}}\right)\\ & +\frac{J_{22}}{det\left[J\right]}m_{2}l_{1}a_{2}\sin\left(\theta_{2}\right){\left(\dot{\theta_{2}}\right)}^{2},\\ f_{2}^{2}\left(\theta_{2},\dot{\theta_{1}},\dot{\theta_{2}}\right) & =-\frac{J_{11}}{det\left[J\right]}m_{2}l_{1}a_{2}\sin\left(\theta_{2}\right){\left(\dot{\theta_{1}}\right)}^{2}-\frac{J_{12}}{det\left[J\right]}2m_{2}l_{1}a_{2}\sin\left(\theta_{2}\right)\left(\dot{\theta_{1}}\dot{\theta_{2}}\right)\\ & -\frac{J_{12}}{det\left[J\right]}m_{2}l_{1}a_{2}\sin\left(\theta_{2}\right){\left(\dot{\theta_{2}}\right)}^{2}, \end{array}\right\}$$

where $det\left[J\right]=$ $J_{11}J_{22}-J_{12}J_{12}$.

These expressions for the components of $f_{2}$ were incorporated into a MATLAB/Simulink GTG simulator as in ([5] Figure 5) and used to generate the geodesic coordinate axes and the geodesic coordinate grid lines for the totally geodesic warped joint-angle manifold (remember a totally geodesic submanifold always exists when the dimension of the submanifold equals the dimension of the manifold).

Using equations

(7) $$\begin{array}{c} x=l_{1}\cos\theta_{1}+l_{2}\cos\left(\theta_{1}+\theta_{2}\right)\\ y=l_{1}\sin\theta_{1}+l_{2}\sin\left(\theta_{1}+\theta_{2}\right) \end{array}$$

and with the origin (0,0) located at the shoulder, we computed the positions of the hand in the Euclidean horizontal (*x-y*)-plane corresponding to points along each of the geodesic coordinate axes and geodesic coordinate grid lines in the (*θ*_1_–*θ*_2_)-joint-angle manifold in Figure 4a. These (*x-y*)-positions are shown in Figure 4b. The corresponding visually-perceived positions of the hand are shown in Figure 4c. Remember that the perceived positions of objects in the intrinsically-warped 3D perceived visual manifold $\left(G,g\right)$ are not the same as their positions in the Euclidean outside world. As outlined in Section 3.1 and Section 4.2 and fully demonstrated in ([6] Section 50) depth is foreshortened to ln r in $\left(G,g\right)$ relative to its depth r in the Euclidean outside world and the angles θ and φ giving the direction of cyclopean gaze in the 3D Euclidean outside world are plotted as distances along straight lines in 3D visual space $\left(G,g\right)$. Consequently, any two radial lines with a fixed angle Δθ between them in Euclidean space are plotted as parallel straight lines in visual space ([6] Figure 8).

To produce Figure 4c the MATLAB/Simulink program was extended to compute the transformation of hand position in the Euclidean horizontal (*x-y*)-plane into its position in the intrinsically-warped 3D visual manifold $\left(G,g\right)$ as the two-DOF arm moved along geodesic pathways in joint-angle space. We use the notation $\left(G,g\right)$ to represent the Riemannian geometry common to all the visual spaces $\left(G_{\left(\psi_{i},p_{i}\right)},g\right)$. First we computed the cyclopean gaze coordinates $\left(r,\;\theta ,\;\varphi \right)$ in Euclidean space for the position of the hand at each point along the geodesic coordinate axes and coordinate grid lines in Figure 4b as seen by a cyclopean eye located at a distance of 0.18 m above the horizontal plane and 0.21 m to the left of the shoulder (i.e., to an estimated position of the cyclopean eye relative to the right arm in the horizontal plane). Next we foreshortened the Euclidean depth r by computing the logarithm ln r for each point. We then used the 3D plot command (*plot3*) to plot ln r as a function of the direction of gaze $\left(\theta ,\;\varphi \right)$ at each point with θ and φ plotted not as angles but as equally-spaced distances along orthogonal straight-line axes. This implies that the position of the cyclopean eye is located at the position (0,0,0) in Figure 4c. The *plot3* command allows the 3D plot to be rotated so it can be seen from the most informative perspective. The resulting rotated 3D plot is shown in Figure 4c. The important thing to notice in Figure 4c is that the coordinate axes and coordinate grid lines spanning a 2D space in both Figure 4a,b map in a one-to-one, onto and invertible fashion onto a 2D submanifold in warped 3D visual space.

The simulation results in Figure 4 demonstrate that, despite warping of the proprioception manifold due to nonlinear inertial interactions between joints in the two-DOF arm as well as intrinsic warping of the 3D visual manifold $\left(G,g\right)$ attributable to the size of images on the retina changing in inverse proportion to Euclidean depth, there exists a smooth, one-to-one, onto, invertible mapping between positions of the hand in the Euclidean outside world and a 2D submanifold of hand positions embedded in the intrinsically-warped 3D visual space $\left(G,g\right)$. In other words, for the warped intrinsic geometry of 3D visual space based on stereopsis, retinal-image disparity and focus control mechanisms of depth perception there exists a smooth, one-to-one, onto and invertible map between the position of the hand in the 3D outside world and its position in the intrinsically warped 3D visual space.

This result can be extended to a 7-DOF arm moving in 3D Euclidean space. If the 7×7 mass-inertia matrix of the arm is known for every configuration of the arm then a system of geodesic coordinate axes and geodesic coordinate grid lines can be generated that span the warped 7D joint-angle (proprioceptive) manifold of the arm just as for the two-DOF arm in Figure 4a. For any position of the arm in the 7D joint-angle space the hand is located at some point $\left(x,y,z\right)$ in 3D Euclidean space. There is redundancy in this map. However, using the same procedure as described above for the two-DOF arm, the position $\left(x,y,z\right)$ of the hand in 3D Euclidean space can be mapped in a one-to-one, onto, invertible and smooth fashion into the intrinsically-warped 3D visual space spanned by coordinates $\left(r,\;\theta ,\;\varphi \right)$. While many different postures of the arm in joint-angle space can locate the hand at the same point $\left(x,y,z\right)$ in 3D Euclidean space the map between hand position in 3D Euclidean space and its position in the intrinsically-warped 3D visual space is smooth, one-to-one, onto and invertible. The redundancy is in the relationship between the 7D joint-angle space and the position of the hand in 3D Euclidean space and not in the mapping between 3D Euclidean space and the intrinsically-warped 3D visual space. To create a one-to-one, onto, invertible proprioceptive-to-vision map we need only to revert to using multiple image points on the arm along with the orthogonalizing procedure described in Section 4.5. Using that procedure every point in the 7D joint-angle space can be mapped smoothly, one-to-one, onto and invertibly onto a visual image of the entire arm in the equivalent 7D warped visual space.

## 7. Task-Related Synergy Selection

Having established that geodesic submanifolds embedded in local regions of the posture-and-place manifold (Ψ,P) compatible with a specified collage of posture-and-place-encoded visual images can be constructed we now turn to the selection of the appropriate movement synergy to achieve a specified visuomotor goal.

### 7.1. Transforming Visuomotor Goals into Movement Synergies

It has long been known that animals can, through trial and error, learn to execute behaviours that lead eventually to required outcomes. Investigations of the mechanisms of animal learning, beginning with the classic works of Thorndike, of Pavlov, and later of Skinner, have been set out by Shah [62] and more recently by Sutton and Barto ([63] Chapters 14 and 15) in extensive accounts of the psychological and neuroscientific bases of *reinforcement learning* (RL). These demonstrate that behavioural and theoretical research into animal learning relates directly to the fundamental concepts of RL where typically agents are employed to learn specific tasks based on predefined rewards and/or punishments.

We propose that through imitation, trial and error, and/or coaching, a type of RL mechanism is involved in selecting a minimum-effort movement synergy compatible with a specified collage of posture-and-place-encoded visual images. The mechanism does not use external rewards such as fruit juice or money but depends on intrinsic rewards generated by a reduction in error between specified required visual outcomes and model-based feedback of actual visual outcomes (see [64] for a review of model-based RL in the human brain). While the complex processes of motor control are mostly not available to consciousness it is important that feedback of actual movement outcomes match as closely as possible the intended ones. It is aversive when actual movement outcomes do not match what was intended. Think of the frustration experienced by those suffering a movement disorder that leaves them able to plan a desired action but unable to execute it appropriately.

When visual feedback does not match the intended visual feedback a strong reaction can ensue. This has been demonstrated in our own work with the observation that, in the face of uncertainty about the control-display relationship in a visual pursuit tracking task, rapid switching between different movement synergies occurs accompanied by slowing and stiffening of movement due to increased gains of tonic stretch reflex loops and increased co-contractions of muscles about elemental movements [65]. This reaction resembles the observed behaviour of people severely disabled with cerebral palsy [66,67] attempting to perform a visual pursuit tracking task [68,69], suggesting difficulty in transforming sequential behavioural goals into appropriately coordinated movements to achieve those goals.

Evidence is ample elsewhere for reactions in the nervous system that facilitate alert and readjustment, a simple example being the alarm experienced on putting a foot on the step that isn’t there. Event-related potentials (P3a and P3b) and fMRI findings reveal frontal lobe dopaminergic activity related to the detection of physically alerting stimuli governing neural responsivity to novelty [70]. Responses involve changes in heart rate and breathing [71], increased climbing fibre activity in the cerebellum (see [72] for review) as well as increased activity of dopamine releasing neurons in the substantia nigra pars compacta and the neighbouring tegmental area [64]. For a comprehensive discussion of dopamine in relation to RL see [62] and ([63] Chapter 15).

An increasing accumulation of data indicates a role for the basal ganglia and the release of dopamine in the planning and execution of short-duration coordinated movements to achieve sequential behavioural goals. From a review of literature Jin and Costa [73] point to increasing evidence for the cortico-basal ganglia-cortical circuits, including the mesencephalic dopamine system, playing a crucial role in generating, shaping, and executing action sequences. They underscore the importance of plasticity in these circuits and suggest its importance in the selection of the neuronal activity patterns underlying the shaping of sequential action. This is further supported in work of Markowitz and colleagues [74] who have shown in mice that the striatum organizes 3D behaviour via moment-to-moment action selection. And in a major review of vision and action Hayhoe states that “in the context of normal behavior humans make continuous sequences of sensory-motor decisions to satisfy behavioral goals and the role of vision is to provide relevant information for making good decisions to achieve those goals” [3] p. 390. Included in this making of good decisions is the brain’s internal reward mechanism and of dopaminergic cells signaling the reward expected from an action.

### 7.2. Model-Based Reinforcement Learning Using an Error-Reducing Association Memory Network

Neuroimaging studies have identified a role for a number of prefrontal cortical areas thought to be involved in high-level response planning including the encoding of rewarding and punishing outcomes. These include the orbitofrontal cortex, medial prefrontal cortex, ventral striatum, anterior insular, and anterior cingulate [75]. Neuroimaging studies have also identified correlates of temporal difference prediction error signals in target areas of dopamine neurons, including ventral and dorsal striatum and in midbrain dopaminergic nuclei. In addition RL value signals have been found in the ventromedial prefrontal cortex and in intra-parietal and supplementary motor cortices (for review see [64]).

In keeping with the above we propose that the prefrontal cortex is involved in specifying visual goals for movement synergies required to perform visually-guided actions (Section 5.1). As set out in Section 4, we propose that these visual goals consist of collages of posture-and-place-encoded visual images of key parts of the environment and key parts of the body in that environment sufficient to span the visual task space for the required movement synergy. Given a visuomotor goal specified this way, we illustrate in Figure 5 a reinforcement learning mechanism able to select a movement synergy compatible with that goal.

Each *collage of visual images spanning task space* is transformed by an *error-reducing association memory network* (see Appendix C) into neural activity representing a set of vectors $\left(c_{i},e_{1},\cdots ,e_{N}\right)$. This can be thought of as a temporospatial pattern of neural activity representing the unique minimum-effort *N*-CDOF movement synergy. As described in Section 5.4, the vectors specify the required initial conditions for a family of *N* GTGs, labelled *GTG submanifold generator* in the figure. The GTGs generate *N* unit-metric-speed geodesics in the posture-and-place manifold (Ψ,P) emanating in orthogonal directions from the initial configuration specified by $c_{i}\in C$ with initial directions specified by orthonormal vectors $\left(e_{1},\cdots ,e_{N}\right)$ in the tangent space $T_{c_{i}}\Gamma$ isomorphic to $T_{c_{i}}\Psi$ as in Section 5.4. These geodesic pathways form geodesic coordinate axes spanning an *N*-dimensional geodesic submanifold embedded in (Ψ,P) (Section 5.4). As explained in Section 3 and Section 5 the posture-and-place geodesic submanifold corresponds to a minimum-effort movement synergy compatible with the specified visual goal. The *posture-and-place submanifold* and the *visual submanifold* are illustrated schematically in the block at bottom right in the figure labelled *partitioned visuospatial memory*.

Every posture and place $\left(\psi_{i},p_{i}\right)$ within the posture-and-place submanifold (i.e., every $\left(\psi_{i},p_{i}\right)$ that can be reached by movements confined to the selected movement synergy) is associated with a partition of visuospatial memory in which a posture-and-place-encoded visual image of objects fixed in the environment and of the body in that environment as seen from that posture and place have been accumulated over time through visual scanning (Section 4). Thus the geodesic posture-and-place submanifold embedded in (Ψ,P) together with its associated posture-and-place-encoded visual-images submanifold act as a model of the relationship between the synergistic movement and its visual outcome. Using visuospatial memory in this way enables the visual outcome to be predicted ahead in time. The associated posture-and-place-encoded visual images form a submanifold of visual images of the environment and of the body in that environment corresponding to posture-and-place points $\left(\psi_{i},p_{i}\right)$ in the selected geodesic submanifold in (Ψ,P).

To achieve selection of an appropriate movement synergy the visual images in the collage of images specifying the goal for the required movement synergy must match images in the visual submanifold retrieved from the partitioned visuospatial memory. A quantitative measure of the extent of mismatch between retrieved visual images and images in the collage is obtained within the block labelled *visual image comparator*. Detailed measures of mismatch between two encoded visual images based on differences between positions of image points in $\left(G_{\left(\psi_{i},p_{i}\right)},g\right)$, differences between image-point vectors, and differences between curvature (shape) at every image point can be computed using Riemannian geometry as described in [6]. However a measure of mismatch between visual images that is simple and adequate for the job can be obtained as follows: The outline of a key object (e.g., a glass in a reach-and-grasp task) and a key part of the body (e.g., the hand grasping the glass) in the retrieved posture-and-place-encoded image can be superimposed on the corresponding outline of the same key object and the same key body part in each of the collaged posture-and-place-encoded images specifying the visual goal. Such outlines correspond to curves in the warped 3D visual space $\left(G_{\left(\psi_{i},p_{i}\right)},g\right)$ as described in ([6] Section 6 and Appendix B). The mean of the metric norm of vectors connecting corresponding image points along the outline of the object and the outline of the body part in the two images provides an adequate measure of visual-image mismatch. This error signal can be appreciated intuitively by looking at the outline of a hand and seeing how this changes when the posture of the hand changes and by looking at the outline of a fixed object and seeing how this changes with changes in the place of the head.

The error signal computed by the *visual image comparator* is used in the block labelled *error-reducing reinforcement system*. This system involves cortico-basal ganglia-cortical loops that release a reinforcer transmitter (e.g., dopamine) onto neurons in the error-reducing association memory network whenever the error signal decreases from one learning cycle to the next. In other words a negative temporal difference of error is rewarded by secretion of dopamine. As explained in Appendix C it is hypothesized that modification of synaptic weights in the network only happens when a reinforcer is present. In trial-and-error learning random variations can be added to $\left(c_{i},e_{1},\cdots ,e_{N}\right)$ at the beginning of the learning sequence. Variance of this random noise can be reduced as the network converges (actually this randomness is inherent in the learning algorithm). The vectors $\left(c_{i},e_{1},\cdots ,e_{N}\right)$ at the output of the error-reducing association memory network specify uniquely a geodesic submanifold embedded in (Ψ,P) as explained in Section 5. From one learning cycle to the next the error-reducing association memory network tunes its synaptic weights to minimize the mismatch error between visual images specifying the visual task space and the visual images reachable from within the selected movement synergy. As the visual mismatch error reduces the movement synergy selected by the vectors $\left(c_{i},e_{1},\cdots ,e_{N}\right)$ approaches closer and closer to the movement synergy compatible with the specified visual goal. Such a learning mechanism is consistent with the decrease in motor variability commonly observed during skill learning [73]. In this way, over time and through experience, the individual accumulates in memory a repertoire of associations between visual goals and compatible minimum-effort movement synergies.

As illustrated at top right in Figure 5 the vectors of initial conditions $\left(c_{i},e_{1},\cdots ,e_{N}\right)$ at the output of the error-reducing association memory network have an additional role to play in transforming planned submovements within the selected movement synergy into appropriately coordinated motor commands to the hundreds of functional muscles of the body. Much of our previous work has been concerned with this transformation (see [47] for review). We will not pursue that aspect here other than to say that the vectors $\left(c_{i},e_{1},\cdots ,e_{N}\right)$ act as an accession code to retrieve from another *association memory network* (top right in Figure 5) previously stored adaptive parameters that preset the tuning of synergy-dependent neural adaptive filters in both sensory and motor systems in readiness to execute submovements planned within the selected movement synergy.

The partitioned visuospatial memory of posture-and-place-encoded visual images of the environment and of the body in that environment plays the role of the model in model-based RL [64]. As explained by Hester and Stone [76], there are advantages for using model-based RL. It can be used to explore configurations where there is uncertainty in the model so as to improve the model’s accuracy as quickly as possible. Existence of a model allows speeded up RL convergence without having to wait for feedback from actions in the outside world. Indeed, just as geodesic trajectories in a Riemannian manifold can be easily time-scaled, model-based feedback in RL can be time-scaled to run in fast-time. Moreover, model-based RL convergence can occur during mental rehearsal without actually performing the movement. Of course, this assumes an accurate representation of the environment and of the body in visuospatial memory. However, as set out in Section 4 and Section 5, the posture-and-place-encoded visual images associated with each posture and place $\left(\psi_{i},p_{i}\right)$ in the posture-and-place manifold (Ψ,P) are being continuously updated as the person moves about in the local environment. This updating occurs independently of the partitioned visuospatial memory acting as a model in model-based RL.

## 8. Discussion

### 8.1. Why Pursue a Theory?

The Riemannian geometry theory developed in this paper concerns the computational processes required to select minimum-effort movement synergies compatible with specified visual goals during performance of natural behaviours. Constructing such a theory involves building bridges between well-established elements of visuomotor science and the abstract but deductively logical structure of Riemannian geometry. These bridges can be taken to be definitions of terminology and notation. They are crafted to facilitate a Riemannian geometry explanation of the computational processes needed to link perception with action in visually-guided behavior. Manifolds, embedded submanifolds, vector fields, metrics, curvature tensors, vector bundles, fibre bundles and so forth are constructs from Riemannian geometry that are of value in a geometrical theory of synergy selection able to handle the complex nonlinearities of both visual and motor systems. There is, however, a caveat. Our theory should not be taken as implying that the visual system actually performs geometric computations. The nervous system has evolved its own methods of processing and transforming visual and motor signals (e.g., by means of feedforward and feedback networks with adaptive synaptic connections [47,77]). The value of the Riemannian theory is its ability to reveal the computational issues involved in transforming perception into action and in its ability to demonstrate the logical feasibility that such computational issues, as complicated as they are, can be resolved. As foreseen long ago by Marr [78], how neural circuits actually implement these computational processes requires a second stage of analysis beyond the computational theory.

We believe that incorporation of Riemannian geometry may eventually mark a Kuhnian paradigm shift [79] in analysis of visually-guided movement. The following parable illustrates the point. In the late 1500s when Galileo was studying gravity he showed experimentally that heavy objects rolled down an inclined plane at the same rate as light objects. He is supposed to have suggested that a cannon ball and a feather dropped from the Leaning Tower of Pisa would hit the ground together. But we had to wait another 100 years before the concepts of force, mass, velocity and acceleration used in modern day explanations of falling objects were defined within the mathematical theory of *differential calculus*. In Galileo’s day those formal notions did not exist. Today such abstract ideas have become commonly accepted and their measurement is pursued in experimental data. We believe that concepts and notions defined within differential geometry (Appendix A) can provide explanations for a long list of visual and motor phenomena including, for example, ability to (i) separate sensory and cognitive components of perception, (ii) construct a 3D perception of the world from a sequence of 2D retinal images, (iii) explain and compute optical flow, (iv) explain illusions of size associated with after images, (v) reconcile contradictory minimum-jerk and minimum-torque-change theories of movement, (vi) explain phenomena independently of the arbitrary coordinate systems chosen for experimental data, (vii) link perception and action taking warping of both visual space and proprioceptive space into account, (viii) learn motor skills through imitation and to visualize the world from another person’s perspective, and (ix) explain dissociation between perception and action in illusions.

### 8.2. A Recap of the Major Features of the Theory

Because of limited central processing resources and redundancy in the neuro-musculo-skeletal system we propose that a movement synergy (i.e., a multi-joint coordination) defined by a set of dynamical constraining relationships between the elemental movements of the body has to be selected before actions can be planned and executed within that synergy.

According to the Riemannian theory, at any given moment the person uses visual gaze and/or visuospatial memory to obtain information in order to specify a collage of posture-and-place-encoded visual images of key objects in the environment and of key parts of the body that span the visual task space for the required movement synergy. Given an initial configuration $c_{i}$ and an appropriate initial unit velocity vector e of all the joint-angle velocities of the body at that initial configuration there exists a unique free-motion trajectory attributable to the mass-inertia properties of the body (i.e., a geodesic trajectory) able to reach any specified target visual-image associated with a target posture-and-place. Such a geodesic trajectory is not only the shortest pathway (i.e., shortest arc length) in curved posture-and-place space between any two points along the pathway but it is also the minimum muscular-effort pathway. Moreover, movement along the pathway can be time scaled (i.e., the metric speed along the pathway can be increased or decreased simply by changing the initial velocity vector e).

For *N*-CDOF movement synergies learned associations in an *error-reducing association memory network* retrieve a unique set of initial-condition vectors $\left(c_{i},e_{1},\cdots ,e_{N}\right)$ associated with a specified collage of posture-and-place-encoded visual images that span the visual task space. The initial-condition vectors $\left(c_{i},e_{1},\cdots ,e_{N}\right)$ preset geodesic trajectory generators (GTGs) to generate *N* geodesic coordinate axes emanating from the specified initial configuration $c_{i}$ in the specified orthonormal directions $\left(e_{1},\cdots ,e_{N}\right)$ tangent to the posture-and-place manifold (Ψ,P) at the initial configuration $c_{i}$. Thus the learned associations within the error-reducing association memory network establishes links between vision and proprioception. Each unit speed geodesic trajectory corresponds to a minimum-effort natural free-motion movement of the body attributable to its mass-inertia characteristics taking gravity and mechanical (mass-inertia) interactions within and between the body and the environment into account. In the outside world it appears as an accelerating and/or decelerating curved trajectory. The *N* geodesics form coordinate axes for an *N*-dimensional geodesic submanifold embedded in the 76D posture-and-place manifold (Ψ,P). This geodesic submanifold defines the unique minimum-effort movement synergy with *N*-CDOFs compatible with achieving the specified visual goal. With this process having specified the spatial plan for the movement (i.e., the required movement synergy), temporal response planning processes then specify a sequence of concatenated goal-directed minimum-metric-acceleration (i.e., minimum-effort) submovements that are executed within the selected movement synergy (i.e., with the same coordination or pattern of constraining relationships between the joint-angle trajectories).

### 8.3. Sequences of Movement Synergies in Natural Behaviour

While the so-called “ballistic” tasks often used in experiments are achieved with a single movement within a synergy, natural behaviour typically consists of concatenated submovements. If carried out within the same synergy they will proceed as described above, but in many cases one or more changes of synergy is necessary to complete the action. The chunking of movement into movement synergies marked by changes in coordination has been extensively observed and described by others [1,3,73,74]. An important feature of movement synergies is that a change from one pattern of multi-joint coordination to another is relatively easy to detect, especially with modern motion analysis technology. Such boundaries between movement synergies can be used to parse movements into movement categories for detailed descriptions of movement during dance, sport, work, rehabilitation, etc. We see selection of goal-directed movement synergies that switch quickly and smoothly from one to the next during natural behaviour as relating to the movement repertoire of monkeys studied by Graziano who states:

“*We filmed a range of primates* ⋯ *[and] were able to film complex behavior including climbing, playing, grooming, foraging, fighting and so on. Much of the video footage was analyzed frame by frame in an attempt to construct a general, qualitative description of the normal movement repertoire of monkeys.* ⋯ *Perhaps the most striking feature of the movement repertoire of monkeys, or of any animal that we observed, was its breakdown into action modes and submodes between which the animal frequently switched with minimal overlap.* ⋯ *Typically an animal switched rapidly among these different action modes.* ⋯ *The episodes of each action mode were brief.* ⋯ *The impression was of a constant changing from one mode to the next*”

([80] pp. 2–5)

This description of monkeys switching quickly between action categories within a repertoire of coordinated multi-joint actions is consistent with the proposal that visually-guided actions involve an ongoing sequence of decisions to select, from a repertoire of learned movement synergies, minimum-effort movement synergies compatible with the evolving visual goals of the visuomotor task.

### 8.4. Other Accounts of Movement Synergy

In their recent review of coordination synergies Bruton and O’Dwyer [81] claim that there are so many different operational definitions of the term “synergy” in the literature that it becomes difficult to use as either a descriptive or explanatory concept. We disagree. We see the various definitions of “synergy” in the wide literature covered in that review as representing different aspects of the comprehensive description given in this paper of movement synergy and its role in movement control. For example, the Riemannian geometry theory predicts (i) a coherent activation in space and time of groups of muscles, (ii) a modular theory of movement control that includes both wired-in and task-dependent synergy generators that switch from one subtask to the next, (iii) that positions along a geodesic pathway in the place-and-posture manifold correspond to single neural commands and reflect translation between task-level goals and execution-level motor commands, (iv) the existence of low-dimensional task-dependent submanifolds embedded in the posture-and-place manifold together with associated submanifolds in 3D visual space that can be related to submanifolds in the uncontrolled manifold hypothesis [82] and (v) that mathematical concepts of nonlinear dynamics are needed to describe and interpret coordination. Apart from (vi) the abundance theory of synergy in which synergies do not eliminate redundant DOFs but instead use “abundant” DOFs to minimize errors [83] and (vii) the notions of “direct perception” and “affordances” emerging from a nonlinear dynamical self-organization within the perception-action cycle proposed in the ecological-dynamical perspective [84], all the above points predicted by the Riemannian geometry theory cover the various operational descriptions of “synergy” reviewed by Bruton and O’Dwyer. Moreover, with the exception of the notion of direct perception, the last two points are not inconsistent with and could be incorporated into the Riemannian theory.

### 8.5. Relationship to Robotic Multi-Joint Movement

We have shown previously [85] that a set of dynamically-coupled elemental movements (i.e., a movement synergy) can be equated to a *nonlinear* dynamical version of the linear transposed matrix $A^{T}$ in a right pseudo-inverse $A^{T}{\left[AA^{T}\right]}^{-1}$ of the rectangular matrix A. The right pseudo-inverse is a mathematical tool used frequently in the field of robotics to compute the relationship between the position of the endpoint of a robot arm in 3D space and the several joint angles of the arm. However, the right pseudo-inverse is a linear tool that does not take the nonlinear gravitational and mass-inertial interaction forces between elemental movements into account. In our Riemannian theory of synergy we replace the transposed matrix $A^{T}$ in the right pseudo-inverse with a nonlinear dynamic movement synergy able to take the curvature of posture space into account. The square matrix ${\left[AA^{T}\right]}^{-1}$ is absorbed by synergy-dependent nonlinear dynamical inverse models in the feedforward-feedback motor control system mentioned in Section 5.5. Thus our proposal adds the effects of nonlinear mass-inertial interactions to the positions and joint-angle formulations commonly used in robotics.

### 8.6. Optical Flow Is Determined by the Intrinsic Riemannian Geometry of 3D Visual Space

As described by Glennerster [86], throughout the animal kingdom a similar pattern of eye movement dominates in creatures as they move. Based on the work of Land [87], he observes that animals fixate while moving, then make a saccade and fixate a new target as they continue to move. He proposes that, when navigating through an environment, animals do this because it leads to special optical flow fields on the retina. For example, as the observer approaches a fixated object the retinal flow is approximately radial expansion outward from the fovea. There are many neurons in the dorsal part of the medial superior temporal cortex sensitive to flow of this type [88,89]. Glennerster goes on to explain that when an observer moves laterally, staying the same distance from the fixation point, there is a pattern of retinal flow in which objects that are closer than the fixation point move one way on the retina while more distant objects move in the opposite direction. Again, there are neurons ideally suited to signaling this type of flow [90]. Glennerster proposes that the two flow components can be detected independently and can be used to signal progress toward the goal with neurons sensitive to lateral motion signaling error. The simplicity of this control strategy relies on the observer fixating on a point during movement.

Moving towards a fixated object causes its image on the retina to expand because of the size-distance relationship introduced by the eye. As noted by Glennerster this is often called retinal optical flow. But it is this size-distance relationship introduced by the eye that underlies the intrinsic warping of 3D visual space described in this paper. If binocular stereopsis, retinal-image disparity and focus control are taken into account as a way of sensing absolute depth, then the approaching object not only appears to loom in size but the rate of looming appears to accelerate. This perceived apparent motion in 3D visual space of a fixated object when moving towards it can likewise be referred to as optical flow (see [91]).

Such optical flow is determined by the intrinsically-warped Riemannian geometry of 3D visual space. While 3D optical flow is generated by motion of the egocentre in the 3D Euclidean outside world it can also be thought of as the apparent motion of points fixed in the environment relative to the egocentre. For example, if the egocentre is moved from A to B in the Euclidean outside world with respect to an external reference frame $\left(X,Y,Z\right)$ then, equivalently, every fixed point in the environment can be represented as moving by the same distance but in the opposite direction in the 3D Euclidean outside world relative to the moving egocentre. When mapped into the intrinsically-warped geometry of 3D visual space the changing cyclopean coordinates $\left(r,\theta ,\varphi \right)$ of all the image points on all the objects fixed in the outside world define an optical flow field in the intrinsically-warped 3D visual space.

The length of a path in the intrinsically-warped 3D visual space is computed by integrating the metric-speed along the path (A.11). But the Riemannian metric $g\left(r,\theta ,\varphi \right)$ defining the warping of the 3D visual space varies inversely with the square of the Euclidean distance r from the egocentre [6]. So metric-speed and hence metric-length in visual space vary depending on the Euclidean distance r from the egocentre. Thus while the relative distance moved by each fixed point in the outside world is the same, the distance moved by each point in the intrinsically-warped 3D visual space varies depending on its distance from the egocentre. Because of this warping of distances along curves in 3D visual space relative to distances in the Euclidean outside world a point moving with constant relative velocity in the outside world will appear to accelerate and/or decelerate in the intrinsically-warped 3D visual space. In other words, the intrinsic warping of 3D visual space introduces illusory changes in size and illusory accelerations and decelerations into 3D optical flow fields. We have quantified the intrinsic warping of 3D visual space by computing the illusory acceleration field (known in Riemannian geometry as the geodesic spray field) for every position and velocity in 3D visual space [6].

Given the proprioceptive-to-vision and vision-to-proprioceptive maps between submanifolds described in Section 6 and illustrated by simulation studies in Figure 4, movement trajectories can be planned in proprioceptive space or in visual space despite nonlinear warping of both spaces. Indeed, images can be transformed back and forth between proprioceptive and visual submanifolds. For example, when juggling three balls in the air [92] a juggler might fix his/her gaze on a point near the apex of the flight path of the balls [93] while, at the same time and without looking at the hands, plan and execute a movement in posture space sensed proprioceptively to throw a ball along a path in visual space from one hand to the other, and to plan a movement of the other hand in proprioceptive space to catch the descending ball in visual space.

### 8.7. Dissociation of Perception and Action

These phenomena raise questions related to the notion of *blind sight* or *sight unseen* described by Goodale and Milner [94,95]. They explored the case of a young woman (DF) who was unable to recognize objects or tell one simple geometric shape from another as a result of brain damage. They showed that she could reach out and grasp objects with dexterity despite being unable to perceive their shape, size, or orientation. As described in their paper entitled “one brain–two visual systems”, even though DF was very poor at describing or demonstrating the orientation of a slot she could still reach out and post a card into the same slot without error. Despite being unable to report the width of a rectangular block, she could still adjust her finger-thumb grip size perfectly in advance of picking it up. She could guide her movements using visual cues of which she seemed completely unaware. From this and their related work they claim, “*ours is a distinction between vision for perception and vision for action*” ([96], p. 660, italics added).

We have addressed how the cyclopean Euclidean distance r can be estimated within the nervous system using stereopsis, retinal-image disparity and focus control mechanisms of depth perception [6]. But in addition the nervous system has many other computational modules able to estimate depth [78]. These employ information derived from occlusions, relative size, texture gradients, shading, height in the visual field, aerial perspective and perspective [97]. The ubiquity of cognitive depth perception is demonstrated by perceived depth in pictures (e.g., television images) and in monocular vision where stereopsis and retinal-image disparity mechanisms are not available. Automatic focus control may provide the means for absolute monocular depth perception. Of the various mechanisms of depth perception, only stereopsis, retinal-image disparity and focus control provide an absolute measure of Euclidean depth. These are based directly on sensory information encoded within afferent signals. The others depend on memorized experience [98], hence the term top-down cognitive mechanisms.

Whenever an estimate of depth derived from one or more of the top-down cognitive mechanisms overrules an estimate of depth obtained through stereopsis, retinal image disparity and/or focus control the geometry of the perceived 3D visual space is altered. Consequently the perceived geometry no longer matches the Riemannian metric g derived directly from afferent signals that encode retinal images changing size in inverse proportion to Euclidean depth. Conscious perception almost certainly includes variations in geometry attributable to cognitive estimates of depth based on past experience and expectations overriding stereopsis, retinal-image disparity and focus control estimates of absolute Euclidean depth. This gives rise to a variety of visual illusions such as the Ames room where a trapezoidal-shaped room is perceived to be shaped like a normal room with parallel walls, horizontal floor and rectangular windows [99,100,101,102]. Similarly, in the expanding virtual room experiment [103], estimates of depth derived from stereopsis and parallax are overruled in favour of a cognitive perception based on the experience that rooms do not expand as we walk about within them. The hollow-mask illusion [104,105] where a concave face mask is seen as being convex most likely occurs because experience tells us that faces are convex. Some top-down cognitive perceptions such as seeing the floor and walls of a normal room as being flat and seeing straight lines as being straight may seem surprising. After all they can hardly be called illusions if floors and walls actually are flat and straight lines actually are straight. Nevertheless, the intrinsic warping of 3D visual space encoded within afferent signals indicates that they should appear curved.

Hatfield [40] described a structure of visual space that takes seeing straight lines as straight into account. In this account visual space is compressed in a Euclidean 3D to 3D projection that allows for railway tracks to converge as they recede in depth while still remaining straight. Similarly, Erkelens [106] described a linear perspective theory that allows perception of slanting planar surfaces as flat surfaces. But such cognitively-modified perceptions introduce paradoxes. For example, despite the fact that by definition a straight line is the only path along which a point can move with zero acceleration, equal increments of distances along a wall appear to change length with depth (e.g., bricks appear to change size) and it is not possible for a point to appear to move along such an apparently straight line in warped 3D visual space with zero perceived acceleration. Conscious perception of a 3D world full of illusions created by top-down cognitive mechanisms of depth perception hardly provides a suitable visual space for the planning and execution of visually-guided movement! Nevertheless, accurate visually-guided movement is possible despite the presence of illusions as shown by the demonstration that, despite observers being unable to resist the compelling “hollow face” illusion, the actions that they direct at the face are not corrupted and arrive at the correct point in the concave hollow mask [105].

We suggest that the dissociation observed experimentally between perception and action in the hollow face illusion, the Ponzo illusion, the Wundt-Jastrow illusion and the Sander parallelogram illusion [105,107,108,109] can be accounted for in terms of the Riemannian theory. Consider, for example, the bimanual grasping experiment recently reported by Ozana and Ganel [109]. Participants grasped rectangular plastic rods placed on a flat background depicting the standard or inverted Ponzo illusion. According to the Riemannian theory, depth perception based on stereopsis, retinal-image disparity and focus control provide estimates of actual Euclidean distance between the person’s egocentre and points on the objects and on the illusory background. Top-down cognitive mechanisms of depth perception, on the other hand, use a variety of cues to generate illusory perceptions of depth. This is analogous to seeing depth in pictures while simultaneously seeing the plane of the picture. As described in Section 6 and verified by simulation of a two-DOF arm, there exists a smooth, one-to-one, onto, invertible relation between the position of the body in the actual 3D Euclidean world and its position in the intrinsically warped 3D visual space. Thus if bimanual movements to grasp the plastic rods are planned within the intrinsically warped 3D visual space derived from sensory inputs before it is modified by top-down cognitive mechanisms, as proposed in the Riemannian theory, then those grasping movements would be accurate and uninfluenced by the illusory perceptions induced by the Ponzo background. The theory therefore predicts the dissociation between action and perception observed in that study. For the same reason we predict that the size of an after image projected onto a picture will not be affected by perceived depth in the picture.

We have shown in Section 6 and Figure 4 that the 3D Riemannian geometry of what we could call *pre-conscious visual space* does provide a smooth, one-to-one, onto, invertible mapping between the actual Euclidean 3D outside world and the place-and-posture-encoded visual images of the body in the intrinsically-warped 3D visual space. We strongly suggest that it is the pre-conscious visual space, derived from afferent signals before its intrinsic geometry is modified by top-down cognitive expectations, that is used for the planning and control of visually-guided movement. We believe that experiments concerned with the dissociation between perception and action in illusions have an important role to play not only in terms of testing the two visual systems theory of Goodale and Milner but also as the first experimental tool with the potential to distinguish between sensory and cognitive components of perception.

### 8.8. Future Directions

Thus far our development of the Riemannian theory of synergy selection has necessarily been limited to the case of visually-guided movement. We are nevertheless aware that an individual’s intention to act may often be based on an assemblage of multimodal sensory cues. Clearly the theory can be developed further to include the integration of posture and place not only with 3D visual space but also with the space of other sensory modalities, in particular tactile space and auditory space. Just as Figure 2 illustrates visual space as a vector bundle representation tied to the configuration of the body in the local environment, so too could a similar representation be constructed for tactile space and auditory space.

This would require the determination of Riemannian metrics to account for the known nonlinear warping of both 3D tactile space [23,110] and 3D auditory space [111]. It would also require a representation of tactile and auditory sensory signals within local clusters (hypercolumns) of tactile and auditory cortical neurons, respectively, as projections onto stochastic tactile and auditory temporospatial features encoded by spatial patterns of synaptic weights on tactile and auditory cortical columns within hypercolumns (analogous to the representation of visual signals in [6]. We see no reason why this cannot be achieved. Thus we envisage the vector-bundle structure illustrated in Figure 2 extended to include parallel representations of 3D visual space, 3D tactile space and 3D auditory space over each posture and place ($\psi_{i},p_{i}$) of the body in the base posture-and-place manifold (Ψ,P). Vector-bundle morphisms similar to those of Figure 2 can then be formed adaptively not only between each and every posture-and-place-encoded partition within visual, tactile and auditory 3D spatial memories but also between the visual, tactile and auditory 3D spaces within each posture-and-place memory partition.

Such a network of multiple vector-bundle morphisms within and between posture-and-place-based partitions of visuospatial, tactuospatial and audiospatial memory would have the capability to provide a multisensory internal representation of moving about within a local 3D environment. This capability allows multimodal perceptions and selection of movement synergies to achieve multimodal sensory goals that would otherwise be unachievable. For example, we cannot see the back of our head yet when it is in contact with a pillow we know its exact location. Likewise we can plan very precise movements to accurately place food into the mouth despite the absence of the mouth from our visual space. The existence of sensory-sensory and sensory motor adaptive maps operating within our Riemannian framework provides a neural mechanism able to account for many such everyday phenomena.

Meanwhile the precise neurophysiological underpinning of such associative maps is a work in progress. As set out by Rizzolatti and colleagues [112] the classical idea of a single multimodal association area in the parietal cortex is now being modified by the notion of there being many maps each encoding space in terms of different effector movements. They offer the view that the sense of space arises from our motor interactions with the world and speak of two types of space, “peripersonal space” within arm reach and “extrapersonal space” beyond. In particular, they cite from monkey studies known overlaps of tactile and visual receptive fields within the inferior premotor cortex that in some cases overlap an auditory field as well. Ultimately a theory such as ours, in present form or extended, must accord with established neurobiology. In that sense it too is a work in progress. It does, however, offer a cohesive mechanism that we have taken care to ensure is *neurally feasible,* that is consistent with much evidence on visually-guided movement and that offers a clear basis for further test and development.

## Data Availability

Not applicable.

## Appendix A. Riemannian Geometry: A Tutorial

Like most of modern mathematics the theorems, lemmas and propositions of Riemannian geometry are deduced logically from the nine basic axioms of set theory (viz., the Zermelo-Fraenkel axioms plus the axiom of choice). The axioms are taken to be self-evidently true. For example, ‘sets exist and x∈X’ means the element x is in the set X. Riemannian geometry develops in layers from set theory through the mathematical fields of topology, topological manifolds, smooth manifolds, and finally Riemannian manifolds. Aspects of all these fields are incorporated in *differential geometry*. In essence, this concerns the calculus of processes taking place in curved spaces. The curvature of space can represent nonlinearity in dynamic processes playing out in that space. Detailed descriptions of differential geometry can be found in many texts [49,50,113,114,115,116,117,118,119,120,121,122,123,124,125]. In this tutorial we focus on the notions from Riemannian geometry most pertinent to this paper.

### A.1. Set Theory

The notation U⊆X means U is a subset of the set X, $X=X_{1}\cup X_{2}$ means that the set X is the union of the sets $X_{1}$ and $X_{2}$, and $X=X_{1}\cap X_{2}$ means that the set X is the intersection of the sets $X_{1}$ and $X_{2}$.

### A.2. Topology

Topology is a mathematical field concerned with the shape of space(s). In topology there is no metric (i.e., no measure of size). Size and shape are seen as independent properties. Topology has been called “elastic sheet geometry”. Space can be stretched, compressed or molded by any amount without cutting, tearing or puncturing into a variety of different shapes. It has been proven (i.e., logically deduced from the basic axioms) that 2D space has different topological structures equivalent to (i) an unbounded plane, (ii) a sphere, (iii) a torus (like the surface of a doughnut), (iv) tori with multiple holes and (v) the connected sum of projective planes (the geometry of a projective plane underlies the theory of perspective and projection of 3D space onto 2D space.) A torus, for example, can be molded (without cutting, tearing or puncturing) into many 2D shapes including the 2D surface of a coffee mug with a handle. The hole associated with the handle corresponds to the hole in the torus. It took over 100 years for topologists to determine all the possible topological structures of 3D space. When it comes to classifying topological structures of spaces with dimension four or greater things become more difficult. It has been proven that there is no algorithm able to classify all the possible topological structures of spaces with dimension four or greater. The best we can do for high dimensional spaces is to recognize that a large number of different topological structures are possible.

### A.3. Topological Spaces

A topological space $\left(X,T\right)$ is a set X endowed with a collection T of open subsets that cover X (i.e., the union of all the open subsets equals X). The open subsets in the topology T satisfy three conditions (i) the set X and the empty set (denoted by ∅) belong to T, (ii) the intersection of any finite number of open subsets in T is an open subset in T, (iii) the union of any number of open subsets in T is an open subset in T. There exists a *basis topology* B on X consisting of a collection of basis open subsets $B_{i}\in$ B such that any other open subset U⊆X can be constructed from a union of basis open subsets $B_{i}\in$ B.

#### A.3.1. Useful Definitions

An open subset U⊆X containing a point p in a topological space X is called a *neighbourhood of* p. An open subset U⊆X containing an arbitrary subset S in a topological space X is called a *neighbourhood of* S. The *interior* of a subset S, denoted by Int S, consists of the union of all the open subsets in X contained in S. Int S is therefore an open subset in X. The *exterior* of a subset S, denoted by Ext S, consists of the union of all the open subsets in X with S removed, denoted by X\S. Ext S is an open subset in X\S. The *closure* of S, denoted by $\bar{S}$, is the intersection of all the closed subsets in X that contain S. A subset $\bar{S}$ is *closed* in X if its complement $X\backslash \bar{S}$ is open in X. The *boundary* of a subset $\bar{S}$ in X, denoted by ∂S, is equal to the difference between the closure of $\bar{S}$ and the interior of S. The boundary ∂S of S is a closed subset in X. A *limit point* p of a subset S in X has a neighbourhood U⊆X that contains at least one other point q∈S. An *isolated point* p of a subset S in X has a neighbourhood U⊆X that contains no other point in S. A topological space X is *compact* if every open cover of X has a finite subcover. An open subset U⊆X is *precompact* if its closure $\bar{U}$ is compact.

#### A.3.2. Maps between Topological Spaces

A map F:X→Y between two topological spaces X and Y is a rule for assigning every point x∈X to a point or points in Y. A map F:X→Y is a continuous map if for every open subset V⊆Y its preimage $F^{-1}\left(V\right)$ (i.e., all points in X that map into V) is an open subset U in X. If F:X→Y is one-to-one (i.e., $F\left(x_{1}\right)=F\left(x_{2}\right)$ implies $x_{1}=$ $x_{2}$) it is called *injective*. If F:X→Y is onto (i.e., each y∈Y receives a mapping from at least one point in X) it is called *surjective*. If F:X→Y is both one-to-one and onto it is called *bijective*. A continuous map F:X→Y that is one-to-one and onto (i.e., bijective) and has a continuous inverse map $F^{-1}:Y\to X$ is known as a *homeomorphism*. Homeomorphisms preserve topological properties. By definition topological properties are those properties of topological spaces that are preserved by homeomorphisms.

#### A.3.3. Open and Closed Maps

A map F:X→Y is an *open map* if for every open subset U⊆X the image set $F\left(U\right)$ is open in Y and it is a *closed map* if for every closed subset K⊆X the image set $F\left(K\right)$ is closed in Y. If F:X→Y is a continuous, injective, open or closed map then it is a *topological embedding*. If F:X→Y is a continuous, surjective, open or closed map then it is a *quotient map*. If F:X→Y is a continuous, bijective, open or closed map then it is a *homeomorphism*.

### A.4. Topological Manifolds

A *topological manifold* M is a topological space endowed with the following set of topological properties preserved by homeomorphic maps.

(i) M is *Hausdorff* which means that for any two points q,p∈M there exist disjoint open subsets U and V in M such that U contains q and V contains p.
(ii) M is *second countable* which means that its basis open subsets $B_{i}\in$ B can be mapped bijectively onto the set of positive integers (i.e., the $B_{i}\in$ B can be counted). Being second countable implies being *first countable* which means that for every point p∈M there is a *neighbourhood basis* consisting of a countable collection of nested neighbourhoods of p such that any other arbitrary neighbourhood of p contains at least one of the neighbourhoods in the neighbourhood basis of p.
(iii) M is *locally Euclidean* which means that for every point p∈M there exists a *coordinate chart* $\left(U,\varphi \right)$ where U is an open subset of M containing the point p known as a *coordinate domain* and φ is a homeomorphic map between U⊆M and $\varphi \left(U\right)\subseteq R^{n}$ in an *n*-dimensional Euclidean space $R^{n}$. This defines the manifold M to be *n*-dimensional. The component functions $\varphi_{i}$ of the homeomorphic map $\varphi :U\to R^{n}$ define a set of orthogonal Cartesian coordinates $\left(u^{1},\cdots ,u^{n}\right)$ on $\hat{U}=\varphi \left(U\right)\subseteq R^{n}$ and a set of curvilinear coordinates $\left(x^{1},\cdots ,x^{n}\right)$ on U⊆M such that $u^{i}=\varphi \left(x^{i}\right)$ and $x^{i}=\;\varphi^{-1}\left(u^{i}\right)$. A collection of coordinate charts $\left(U_{i},\varphi_{i}\right)$ i=1,2,⋯, that cover M is called an *atlas*.
(iv) M is *locally path-connected* (i.e., its basis open subsets $B_{i}\in$ B are path-connected).
(v) M is *locally compact* (i.e., its basis open subsets $B_{i}\in$ B are precompact).
(vi) The combination of being second countable, locally compact and Hausdorff means that a topological manifold M is *paracompact* (i.e., every open cover of M has a *locally finite refinement* $B_{i}\in$ B (i.e., every open subset U⊆M can be constructed from a union of a finite number of basis open subsets $B_{i}$)).

### A.5. Smooth Manifolds

*Smooth manifolds* are topological manifolds endowed with a differentiable structure that allows differentiation of continuous real-valued functions f:M→R on M and differentiation of continuous maps $F:M_{1}\to M_{2}$ between smooth manifolds $M_{1}$ and $M_{2}$. A continuous map $F:U\subseteq R^{n}\to R^{k}$ between Euclidean spaces $R^{n}$ and $R^{k}$ is said to be smooth (i.e., class $C^{\infty }$ or infinitely continuously differentiable) if all of its component functions have continuous partial derivatives of all orders. A diffeomorphism is a bijective smooth map whose inverse is also smooth.

Two coordinate charts $\left(U,\varphi \right)$ and $\left(V,\psi \right)$ on a n-manifold M are said to be *smoothly compatible* if (i) either the coordinate domains U and V do not overlap or, (ii) if they do overlap, then the *transition functions* $\left(\psi \circ \varphi^{-1}\right)$ and $\left(\varphi \circ \psi^{-1}\right)$ (notice the order) between $\varphi \left(U\cap V\right)$ and $\psi \left(U\cap V\right)$ in Euclidean spaces $R^{n}$, respectively, are smooth (i.e., diffeomorphisms). The symbol ∘ in these equations denotes “composition” or one map followed by the other in reverse order. These transition functions between coordinate charts correspond to coordinate transformations. Smooth manifold theory is coordinate independent. The maps $\varphi :U\to R^{n}$ and $\psi :V\to R^{n}$ are diffeomorphisms.

An *atlas for* M is said to be *smoothly compatible* if all of its coordinate charts are smoothly compatible. A differentiable structure on M is a *maximal smooth atlas* that contains all possible smoothly compatible coordinate charts on M. We usually just say that a manifold M is a *smooth manifold* with all its topological properties and differentiable structure understood. It is often the case that the geometry of a smooth manifold is described in terms of a single coordinate chart with its smooth differentiable structure understood.

At each point $\varphi \left(p\right)\in R^{n}$ in a coordinate chart $\varphi :U\to R^{n}$ on a smooth manifold M there exists an *n*-dimensional tangent vector space $T_{\varphi \left(p\right)}R^{n}$ spanned by a set of orthonormal coordinate basis vectors $\left(\frac{\partial }{\partial u_{1}},\cdots ,\frac{\partial }{\partial u_{n}}\right)$. At each point p∈M in U⊆M there exists an *n*-dimensional tangent vector space $T_{p}M$ spanned by linearly independent basis coordinate vectors $\left(\frac{\partial }{\partial x_{1}},\cdots ,\frac{\partial }{\partial x_{n}}\right)$. Because the coordinates $\left(x^{1},\cdots ,x^{n}\right)$ on U⊆M are curvilinear the coordinate basis vectors $\left(\frac{\partial }{\partial x_{1}},\cdots ,\frac{\partial }{\partial x_{n}}\right)$ spanning the tangent space $T_{p}M$ are not orthonormal. The angles between the coordinate basis vectors $\left(\frac{\partial }{\partial x_{1}}=\partial_{1},\cdots ,\frac{\partial }{\partial x_{n}}=\partial_{n}\right)$ change from point to point in the coordinate domain U.

### A.6. Smooth Maps between Smooth Manifolds

If M and N are smooth manifolds a continuous map F:M→N is said to be smooth if for every p∈M there exist smooth coordinate charts $\left(U,\varphi \right)$ for M containing p and $\left(V,\psi \right)$ for N containing $F\left(p\right)$ such that $F\left(U\right)\subseteq V$ and the composite map $\hat{F}=\psi \circ F\circ \varphi^{-1}$ (notice the order) is smooth between the Euclidean spaces $\varphi \left(U\right)$ and $\psi \left(V\right)$. The composite map $\hat{F}=\psi \circ F\circ \varphi^{-1}$ is called a *coordinate representation* of F. If $\hat{F}$ is smooth then F:M→N is said to be smooth. The set of all smooth maps between smooth manifolds M and N is denoted by $C^{\infty }\left(M,N\right)$ and the vector space of all smooth real-valued functions f:M→R is denoted by $C^{\infty }\left(M\right)$.

### A.7. Tangent Vectors and Cotangent Vectors

Let M be a smooth manifold. For every point p∈M a tangent vector v at p is a linear map $v:C^{\infty }\left(M\right)\to R$ known as a *derivation* at p, meaning that for $f,g\in C^{\infty }\left(M\right)$ the map v satisfies the product rule $v\left(fg\right)=f\left(p\right)\left(vg\right)+\left(vf\right)g\left(p\right)$. (Notice this is equivalent to the Leibnitz derivation for the partial derivative of a product of continuous functions fg given by $\frac{\partial }{\partial x}fg=f\left(\frac{\partial }{\partial x}g\right)+\left(\frac{\partial }{\partial x}f\right)g$). The set of all tangent vectors at p is denoted by $T_{p}M$ and called the *tangent vector space* at p. The vectors ∂∂xi=∂i for i=1,⋯,n form a *basis of coordinate vectors* spanning the tangent vector space $T_{p}M$. Once a smooth chart $\left(U,\varphi \right)$ has been chosen then any tangent vector $v\in T_{p}M$ at the point p∈M can be written as $v=v^{i}\partial_{i}{}_{p}=v^{1}\partial_{1}{}_{p}+\cdots +v^{n}\partial_{n}{}_{p}$ where the components $v^{i}$ are computed by $v^{i}=v\left(x^{i}\right)=dx^{i}\left(v\right)$. (Notice the equality $v=v^{i}\partial_{i}{}_{p}$ uses the summation convention, when an index i appears as both a superscript and a subscript in the same expression it implies summation over all values of i.) For every p∈M the *dual covector space* $T_{p}^{*}M$ is spanned by coordinate basis covectors $\left(dx^{1},\cdots ,dx^{n}\right)$ where $\langle dx^{i},\partial_{j}\rangle =\delta_{j}^{i}=\left\{\frac{1\;if\;i=j}{0\;if\;i\neq j}\right.$. For every $f\in C^{\infty }\left(M\right)$ and p∈M there is a covector $df_{p}\in T_{p}^{*}M$ called the differential of f at p defined by $df_{p}\left(v\right)=vf$ for all $v\in T_{p}M$

### A.8. Smooth Submanifolds

A smooth map F:M→N between smooth manifolds M and N is said to have *constant rank* if the linear tangent map $dF_{p}$ (i.e., the differential of the map F:M→N at the point p) between the tangent spaces $T_{p}M$ and $T_{F\left(p\right)}N$ has the same rank at every p∈M.

F is called a *submersion* if its differential $dF_{p}$ is surjective at each point p, or equivalently, if $dF_{p}$ has constant rank equal to the dimension of N denoted dim N. F is called an *immersion* if its differential $dF_{p}$ is injective at each point p, or equivalently, if $dF_{p}$ has constant rank equal to dim M.

If F:M→N is an immersion then $F\left(M\right)$ is an immersed submanifold in N. Because of the large number of possible topological structures of M and N an immersed submanifold $F\left(M\right)$ can fold on itself and can intersect with itself. This prevents an open subset $U\subseteq F\left(M\right)$ from intersecting with an open subset V⊆N. In this case we say that an immersed submanifold cannot inherit a *subspace topology* from the ambient manifold although if an open subset in an immersed submanifold is small enough so that it does not fold on itself then it can inherit a subspace topology. An immersed submanifold $F\left(M\right)$ can be endowed with a topology T other than a subspace topology inherited from the ambient manifold in which it is immersed. A smooth map F:M→N is called a *smooth embedding* if it is an injective immersion that is also a diffeomorphism onto its image $F\left(M\right)$ in N and has the subspace topology inherited from the ambient manifold N. In other words, it does not fold on itself.

### A.9. Smoothly Embedded Submanifolds

Suppose M is a smooth n-dimensional manifold. A smoothly immersed m-dimensional submanifold $\tilde{M}$ of M is an m-dimensional topological submanifold endowed with a smooth structure such that the inclusion map $\iota_{\tilde{M}}:\tilde{M}\to M$ is a smooth immersion. $\tilde{M}$ is called a *smoothly embedded submanifold* of M if the inclusion map $\iota_{\tilde{M}}:\tilde{M}\to M$ is a smooth embedding (i.e., the topology on $\tilde{M}$ is the subspace topology inherited from the ambient manifold M). The codimension of a smoothly embedded submanifold $\tilde{M}$ is the difference between dim M and dim $\tilde{M}$. A submanifold of codimension 1 is known as a hypersurface. The word “submanifold” always means an immersed submanifold; an embedded submanifold is a special case.

### A.10. Slice Coordinates

Let M be a smooth n-dimensional manifold and let $\tilde{M}$ be a smoothly embedded m-dimensional submanifold in M. Then for each $p\in \tilde{M}$ there exists a neighbourhood U of p in M with smooth coordinates $\left(x^{1},\cdots ,x^{n}\right)$ for U⊆M such that the first m coordinates $\left(x^{1},\cdots ,x^{m}\right)$ span the subspace $U\cap \tilde{M}$ in the smoothly embedded submanifold $\tilde{M}$.

### A.11. Riemannian Manifolds

A *Riemannian manifold* $\left(M,g\right)$ is a smooth manifold M endowed with a *Riemannian metric* $g\left(p\right)$ at every p∈M. A Riemannian metric $g\left(p\right)$ is a symmetrical, positive definite, nonsingular, *2-tensor field* that varies smoothly on the manifold $\left(M,g\right)$. Such a 2-tensor field is equivalent to defining a metric inner product $g\left(X,Y\right)\;=\langle X,Y\rangle_{g}$ between any two vectors X and Y in the tangent space $T_{p}M$ at every point $p\in \left(M,g\right)$. The metric $g\left(p\right)$ at each point $p\in \left(M,g\right)$ allows the *metric norm* (length) ∥X∥ =〈X,X〉g12 of any vector $X\in T_{p}M$ and the angle $\cos\theta =\frac{\langle X,Y\rangle_{g}}{∥X∥\cdot ∥Y∥}$ between any two vectors X and Y in $T_{p}M$ to be computed. Because $g\left(p\right)$ is nonsingular at every point $p\in \left(M,g\right)$ the inverse metric $g^{-1}\left(p\right)$ also exists at each point $p\in \left(M,g\right)$.

In any coordinate chart $\left(U,\varphi \right)$ on $\left(M,g\right)$ with coordinates $\left(x^{1},\cdots ,x^{n}\right)$ on the coordinate domain U the metric $g=g_{ij}dx^{i}dx^{j}$ where the $g_{ij}$ are the components of the metric. Thus g is an n×n, symmetric, positive definite, nonsingular matrix that varies smoothly with $p\in \left(M,g\right)$. The arc length S between any two points a and b along any unit-speed curve $\gamma \left(s\right)$ in $\left(M,g\right)$ is given by S=∫ab〈γ˙,γ˙〉g12ds. This equation provides a type of “tape measure” that allows distances between points along curves and the sizes of submanifolds in $\left(M,g\right)$ to be measured.

For a mechanical system (like the human body) the kinetic energy of motion is given by 12〈Jv,v〉=12〈gv,v〉 where v is velocity, J is mass-inertia, and g is the *kinetic-energy Riemannian metric*. Thus, for a mechanical system, the mass-inertia J is the kinetic-energy Riemannian metric.

### A.12. Graphs of Submanifolds

Suppose M is a smooth m-dimensional manifold, and N is a smooth n-dimensional manifold. Then the Cartesian product space M×N is a $\left(m+n\right)$-dimensional smooth manifold. Let U⊆M be an open subset of M, and let f:U→N be a smooth map. Let $\Gamma \left(f\right)\subseteq M\times N$ denote the graph of f:U→N (i.e., $\Gamma \left(f\right)=\left\{\left(x,y\right)\in M\times N:x\in U,y\in f\left(x\right)\right\}$). Then $\Gamma \left(f\right)$ is a smoothly embedded submanifold of M×N diffeomorphic to U⊆M.

### A.13. Vector Bundles

The tangent bundle of M, denoted by TM, is the disjoint union ∐ of the all the tangent vector spaces $T_{p}M$ at all points p∈M, (i.e., $TM={∐}_{p\in M}T_{p}M$). The tangent bundle TM is both a union of vector spaces $T_{p}M$ and a smooth manifold TM. This kind of structure, called a *vector bundle*, is extremely common in differential geometry. For any positive integer k, a smooth vector bundle of rank-k is a pair of smooth manifolds E and M together with a smooth surjective map π:E→M. For each p∈M, the set $E_{p}=\pi^{-1}\left(p\right)$ is endowed with the structure of a k-dimensional real vector space $R^{k}$. The manifold M is called the *base of the vector bundle*, E is called the *total space* (it includes both M and the disjoint union of all the k-dimensional vector spaces $E_{p}$), and π:E→M is its *projection* onto M. Each vector space $E_{p}=\pi^{-1}\left(p\right)$ is called the *fibre of* E *over* p. In other words, at each point p in the smooth base manifold M there is attached a k-dimensional vector space $E_{p}$.The base manifold M and the collection of k-dimensional vector spaces $E_{p}$ over M together form a vector bundle E. The map π:E→M takes each element of the vector space $E_{p}$ to the point p∈M. E has a unique smooth structure making it a smooth vector bundle of rank-k over M with π:E→M a smooth surjective projection. A smooth *section* of E (i.e., a smooth vector field V over M) is a smooth map σ:M→E such that $\pi \circ \varphi =Id_{M}$ the identity map on M, or equivalently, $\varphi \left(p\right)\in E_{p}$ for every p∈M.

If π:E→M is a smooth vector bundle then the set of smooth sections of E (i.e., all the possible vector fields V over M), denoted by ΓE, is a vector space under point-wise addition and multiplication by constants. The *zero section* of ΓE defined by $\zeta \left(p\right)=0\in E_{p}$ for all p∈M is diffeomorphic to M.

Suppose M is a smooth manifold and $\tilde{M}\subseteq M$ is a smoothly immersed or a smoothly embedded submanifold of M. If π:E→M is any smooth rank-k vector bundle over M, then we obtain a smooth vector bundle $\pi_{\tilde{M}}:E_{\tilde{M}}\to \tilde{M}$ of rank-k over $\tilde{M}$ whose total space is $E_{\tilde{M}}=\pi^{-1}\left(\tilde{M}\right)$. The fibre of $\pi_{\tilde{M}}:E_{\tilde{M}}\to M$ at each $p\in \tilde{M}$ is exactly the fibre of E. Every smooth section of E*restricts* to a smooth section of $E_{\tilde{M}}$ and, in most cases, smooth sections of $E_{\tilde{M}}$*extend* to smooth sections of E, at least locally near $\tilde{M}$.

### A.14. Vector Bundle Morphisms

Suppose $\pi_{i}:E_{i}\to M_{i}$ and $\pi_{j}:E_{j}\to M_{j}$ are two vector bundles and suppose $H:E_{i}\to E_{j}$ is a *vector bundle morphism* (i.e., a smooth map between the vector bundles). Then (i) H preserves the zero section $H:E_{i0}\to E_{j0}$. (Since the zero section is diffeomorphic to the base manifold it follows that $H:M_{i}\to M_{j}$). (ii) $H:E_{i}\to E_{j}$ induces a unique mapping $H_{1}:M_{i}\to M_{j}$ such that $\pi_{j}\circ H_{2}=H_{1}\circ \pi_{i}$ where $H_{2}:\Gamma E_{i}\to \Gamma E_{j}$.

### A.15. Covariant Derivatives

Consider a curve $\gamma \left(t\right)$ parameterized by time t in a manifold $\left(M,g\right)$. A velocity vector $\dot{\gamma }\left(t\right)$ tangent to the curve $\gamma \left(t\right)$ can be computed at every point along the curve. A problem that does not occur in flat Euclidean space occurs when we attempt to compute the acceleration $\ddot{\gamma }\left(t\right)$ at every point along the curve. Computing the acceleration $\ddot{\gamma }\left(t_{0}\right)$ at each point $\gamma \left(t_{0}\right)$ along the curve $\gamma \left(t\right)$ in Euclidean space involves computing the difference $\dot{\gamma }\left(t_{0}+\Delta t\right)-\dot{\gamma }\left(t_{0}\right)$, dividing by Δt and taking the limit as Δt→0. However in a Riemannian manifold $\left(M,g\right)$ the velocity vectors $\dot{\gamma }\left(t_{0}+\Delta t\right)$ and $\dot{\gamma }\left(t_{0}\right)$ are in completely different disjoint tangent vector spaces $T_{\dot{\gamma }\left(t_{0}+\Delta t\right)}M$ and $T_{\dot{\gamma }\left(t_{0}\right)}M$ with completely different coordinate basis vectors $\left(\partial_{1},\cdots ,\partial_{n}\right)$ and so cannot be subtracted!

A new type of metric-acceleration vector $\nabla_{\dot{\gamma }}\dot{\gamma }$ known as a *covariant derivative* is required that takes the changing coordinate basis vectors $\left(\partial_{1},\cdots ,\partial_{n}\right)$ of the tangent vector spaces along the curve $\gamma \left(t\right)$ in the curved Riemannian manifold $\left(M,g\right)$ into account. This gives rise to the notion of a *connection* ∇ between tangent spaces on the manifold $\left(M,g\right)$.

A connection ∇ is not an inherent property of a manifold. It has to be imposed on the manifold. The covariant derivative $\nabla_{\partial_{k}}\partial_{j}$ giving the change in the basis vector $\partial_{j}$ for movement in the direction $\partial_{k}$ at each point $p\in \left(M,g\right)$ is defined to be $\nabla_{\partial_{k}}\partial_{j}=\Gamma_{jk}^{1}\partial_{1}+\cdots +\Gamma_{jk}^{n}\partial_{n}$. The coefficients of the connection $\Gamma_{jk}^{i}$ for i,j,k=1,⋯,n at each point $p\in \left(M,g\right)$ are known as the Christoffel symbols for the metric $g\left(p\right).$ Together they define the connection ∇ on the manifold $\left(M,g\right)$.

In a Riemannian manifold $\left(M,g\right)$ the Christoffel symbols at each $p\in \left(M,g\right)$ can be computed from the equation $\Gamma_{jk}^{i}=\frac{1}{2}g^{im}\left(\frac{\partial g_{mj}}{\partial x^{k}}+\frac{\partial g_{mk}}{\partial x^{j}}-\frac{\partial g_{jk}}{\partial x^{m}}\right).\;$ The covariant derivative $\nabla_{\dot{\gamma }}\dot{\gamma }$ at each point $\gamma \left(t_{0}\right)$ along the curve $\gamma \left(t\right)$ in the manifold $\left(M,g\right)$ is then given by the equation $\nabla_{\dot{\gamma }}\dot{\gamma }=\ddot{\gamma }-f_{2}\left(\gamma ,\dot{\gamma }\right)$ where $f_{2}\left(\gamma ,\dot{\gamma }\right)=-\frac{dx^{k}}{dt}$ $\frac{dx^{j}}{dt}\Gamma_{jk}^{i}\partial_{i}$ summed over all i,j,k=1,⋯,n.

$f_{2}\left(p,v\right)$ is an acceleration vector known as the acceleration component of the *geodesic spray field* that can be pre-computed for every point $p\in \left(M,g\right)$ and every vector v in the tangent vector space $T_{p}M$. Thus, given the Riemannian metric $g\left(p\right)$ at every point p on the manifold $\left(M,g\right)$, all the Christoffel symbols $\Gamma_{jk}^{i}$ can be computed and knowing the Christoffel symbols at every $p\in \left(M,g\right)$ the geodesic-spray acceleration vector field $f_{2}\left(p,v\right)$ can be computed for every $p\in \left(M,g\right)$ and for every tangent vector $v\in T_{p}M$ in every tangent vector space $T_{p}M$.

### A.16. Curvature

The *curvature* of a Riemannian manifold at each point $p\in \left(M,g\right)$ provides a measure of the failure of second covariant derivatives $\nabla_{X}\nabla_{Y}$ to commute at that point where X and Y are arbitrary tangent vectors. It also provides a measure of the fact that in a curved manifold parallel translation (described below) is path dependent. Even in a flat Euclidean space, given arbitrary tangent vectors X and Y, we obtain $\nabla_{X}\nabla_{Y}-\nabla_{Y}\nabla_{X}\;=\nabla_{\left[X,Y\right]}$ where $\left[X,Y\right]=XY-YX$ is known as the commutator bracket or Lie bracket. If X and Y are tangent vectors then the Lie bracket $\left[X,Y\right]$ is also a tangent vector. Thus, for a flat Euclidean space, we can write $\nabla_{X}\nabla_{Y}-\nabla_{Y}\nabla_{X}-\nabla_{\left[X,Y\right]}\;=0$. This provides a criterion for *flatness*.

We define a curvature operator at each point $p\in \left(M,g\right)$ on a manifold $\left(M,g\right)$ to be $R\left(X,Y\right)=\nabla_{X}\nabla_{Y}-\nabla_{Y}\nabla_{X}-\nabla_{\left[X,Y\right]}$. It operates on any vector Z in the tangent space $T_{p}M$ and transforms it to another vector in $T_{p}M$. The curvature operator $R\left(X,Y\right)$ is therefore an *endomorphism*. If a manifold $\left(M,g\right)$ is isomorphic to a flat Euclidean space then $R\left(X,Y\right)Z=0$ at every point $p\in \left(M,g\right)$ and we can say that the manifold $\left(M,g\right)$ is flat and totally parallel. However, if $R\left(X,Y\right)Z\neq 0$ at the point $p\in \left(M,g\right)$ then we say that the manifold $\left(M,g\right)$ is curved (or warped) at the point $p\in \left(M,g\right)$ and parallel translation is path dependent.

The Riemann curvature tensor $R_{m}\left(X,Y,Z,W\right)$ operates on four vectors $X,Y,Z,W\in \;T_{p}M$ and transforms them into a real number. That number provides a measure of the curvature at $p\in \left(M,g\right)$. It is defined by the metric inner product $R_{m}\left(X,Y,Z,W\right)=R\left(X,Y\right)Z,W_{g}$ and so depends on the Riemannian metric g and the way g changes from point to point in the manifold. $R_{m}\left(X,Y,Z,W\right)$ has important symmetries:

(i) $R_{m}\left(X,Y,Z,W\right)=-R_{m}\left(X,Y,W,Z\right)$,
(ii) $R_{m}\left(X,Y,Z,W\right)=-R_{m}\left(Y,X,Z,W\right)$,
(iii) $R_{m}\left(X,Y,Z,W\right)=R_{m}\left(Z,W,X,Y\right)$, and
(iv) $R_{m}\left(X,Y,Z,W\right)+R_{m}\left(Y,Z,X,W\right)+R_{m}\left(Z,X,Y,W\right)=0$.

### A.17. Geodesics and Parallel Translation

We have seen that the covariant derivative at each point $\gamma \left(t_{0}\right)$ along a curve $\gamma \left(t\right)$ in a curved Riemannian manifold $\left(M,g\right)$ is given by the equation $\nabla_{\dot{\gamma }}\dot{\gamma }=\ddot{\gamma }-f_{2}\left(\gamma ,\dot{\gamma }\right)$. If this covariant derivative (metric-acceleration) equals zero (i.e., $\nabla_{\dot{\gamma }}\dot{\gamma }=\ddot{\gamma }-f_{2}\left(\gamma ,\dot{\gamma }\right)=0$) then the curve $\gamma \left(t\right)$ is called a *geodesic* of the manifold $\left(M,g\right)$ and the tangent velocity vector $\dot{\gamma }\left(t\right)$ is said to be *parallel translated* along the curve $\gamma \left(t\right)$. Since the metric-acceleration is zero at every point along the curve it follows that a geodesic corresponds to a constant speed straight line pathway in the curved manifold $\left(M,g\right)$. If $\nabla_{\dot{\gamma }}X=0$ and $\nabla_{\dot{\gamma }}Y=0$ for any other vector fields X and Y along the geodesic curve $\gamma \left(t\right)$ then the vectors X and Y are also said to be parallel translated along $\gamma \left(t\right)$. The metric inner products $\langle \dot{\gamma },X\rangle_{g},\;\langle \dot{\gamma },Y\rangle_{g},\;\langle X,X\rangle_{g},\;\langle Y,Y\rangle_{g},$ and $\langle X,Y\rangle_{g}$ are preserved by parallel translation along a geodesic $\gamma \left(t\right)$. In other words, the lengths (norms) of the vectors $\dot{\gamma },\;X$ and Y and the angles between them are preserved by parallel translation along a geodesic $\gamma \left(t\right)$. Equivalently, any orthonormal frame of parallel translated vectors $\left(e_{1},e_{2},\cdots ,e_{m}\right)$ along a geodesic $\gamma \left(t\right)$ remains orthonormal at all points along the geodesic.

We have also seen that for a geodesic $\gamma \left(t\right)$ in $\left(M,g\right)$ the covariant derivative $\nabla_{\dot{\gamma }}\dot{\gamma }=\ddot{\gamma }-f_{2}\left(\gamma ,\dot{\gamma }\right)=0$ for every point along the curve. It follows that for a geodesic $\gamma \left(t\right)$, $\ddot{\gamma }=f_{2}\left(\gamma ,\dot{\gamma }\right)$. Thus the acceleration $\ddot{\gamma }$ for a geodesic γ can be generated at the input to an array of double integrators by nonlinear feedback $f_{2}\left(\gamma ,\dot{\gamma }\right)$ of position γ and velocity $\dot{\gamma }$ from the outputs of the integrators. Given an initial position $\gamma \left(t_{0}\right)$ and an initial velocity $\dot{\gamma }\left(t_{0}\right)$ the corresponding geodesic trajectory $\gamma \left(t\right)$ is generated by the array of double integrators. We refer to such an array of double integrators as a *geodesic trajectory generator* (GTG). Notice that only the initial position $\gamma \left(t_{0}\right)$ and an initial velocity $\dot{\gamma }\left(t_{0}\right)$ are required to generate a geodesic trajectory in a Riemannian manifold $\left(M,g\right)$.

### A.18. Variation through Geodesics

A *variation* of an arbitrary curve $\alpha \left(x^{1}\right)$ parameterized by arc length $x^{1}$ in a Riemannian manifold $\left(M,g\right)$ (i.e., variation of a unit speed curve) is a smooth map Γ:$\;R^{2}\to M$ between a 2-dimensional Euclidean space $R^{2}$ and the Riemannian manifold $\left(M,g\right)$ (i.e., Γ:$\;\left[a,b\right]\times J\to M$ where $\left[a,b\right]$ is a closed interval between a and b on a real-number line R and J is some interval on an orthogonal real-number line R containing 0). For all $x^{1}$ in the closed interval $\left[a,b\right]$ the map $\Gamma \left(x^{1},0\right)=\alpha_{0}\left(x^{1}\right)$. Such a variation is well known in differential geometry. It is used, for example, to compute minimum length pathways and minimum energy pathways in curved spaces using the calculus of variations. The map $\Gamma \left(x^{1},x^{2}\right)=\alpha_{x^{2}}\left(x^{1}\right)$ where $x^{2}$ is held constant at different values in the interval J gives a family of curves $\left\{\alpha_{x^{2}}\left(x^{1}\right)\right\}$ defined on the closed interval $\left[a,b\right]$. If all the curves $\alpha_{x^{2}}\left(x^{1}\right)$ are geodesics then $\Gamma \left(x^{1},x^{2}\right)$ is called a variation through geodesics.

#### A.18.1. Variation at the Beginning Point

Let $\alpha_{0}\left(x^{1}\right)$ be a geodesic in $\left(M,g\right)$ with initial values $\alpha_{0}\left(0\right)=p$ and $\dot{\alpha_{0}}\left(0\right)=v\in T_{p}M$. Let $z,w\in T_{p}M$. Let $\beta_{0}\left(x^{2}\right)$ be a curve such that $\beta_{0}\left(0\right)=p$ and $\dot{\beta_{0}}\left(0\right)=z$. This is not necessarily a geodesic but it can be. Let $\zeta \left(x^{2}\right)=P_{0,\beta_{0}}^{x^{2}}\left(v\right)+x^{2}P_{0,\beta_{0}}^{x^{2}}\left(w\right)$ where $P_{0,\beta_{0}}^{x^{2}}$ represents parallel transport along $\beta_{0}$ from 0 to $x^{2}$. Then $\Gamma \left(x^{1},x^{2}\right)$ equals a family of geodesics $\left\{\alpha_{x^{2}}\left(x^{1}\right)\right\}$ with initial conditions $\alpha_{x^{2}}\left(0\right)=\beta_{0}\left(x^{2}\right)$ and $\dot{\alpha_{x^{2}}}\left(0\right)=\zeta \left(x^{2}\right)$. $\Gamma \left(x^{1},x^{2}\right)$ is then called *a variation of the geodesic* $\alpha_{0}\left(x^{1}\right)$ *through the family of geodesics* $\left\{\alpha_{x^{2}}\left(x^{1}\right)\right\}$ and each geodesic $\alpha_{x^{2}}\left(x^{1}\right)$ is the unique geodesic such that $\alpha_{x^{2}}\left(0\right)=\beta_{0}\left(x^{2}\right)$ and $\dot{\alpha_{x^{2}}}\left(0\right)=\zeta \left(x^{2}\right)$. In particular, if w=0, then $\dot{\alpha_{x^{2}}}\left(0\right)=P_{0,\beta_{0}}^{x^{2}}\left(v\right)$.

#### A.18.2. Properties of Variation through Geodesics $\Gamma \left(x^{1},x^{2}\right)$

Let $X=\frac{\partial \Gamma_{x^{2}}\left(x^{1}\right)}{\partial x^{1}}$ and $Y=\frac{\partial \Gamma_{x^{1}}\left(x^{2}\right)}{\partial x^{2}}$ be vector fields tangent to $\Gamma \left(x^{1},x^{2}\right)$ such that X is tangent to the horizontal geodesic coordinate grid lines and Y is tangent to the vertical coordinate grid lines. Then the variation through geodesics $\Gamma \left(x^{1},x^{2}\right)$ has the following properties: (i) $\nabla_{X}Y=\nabla_{Y}X$, (ii) $\nabla_{Y}\langle X,X\rangle_{g}=2\langle \nabla_{Y}X,X\rangle =2\langle \nabla_{X}Y,X\rangle$, (iii) $\nabla_{X}\nabla_{Y}-\nabla_{Y}\nabla_{X}=R\left(X,Y\right)$, (iv) $\frac{\partial \Gamma \left(x^{1},0\right)}{\partial x^{2}}=Y\left(x^{1}\right)=$ *Jacobi lift* at each point $x^{1}$ along the geodesic $\alpha_{0}\left(x^{1}\right)$ (see Appendix B for more detail). The theory of Jacobi lifts is well known in Riemannian geometry. Jacobi lifts quantify the deviation or convergence of local geodesics in a variation through geodesics. They are used amongst other things to compute tidal forces acting on the oceans of the world.

## Appendix B. Mathematical Properties of Variations through Geodesics

Much is known in Riemannian geometry about the geometrical properties of *variations through geodesics*. We set out some of these properties here. For proofs and more detail see Lee [118], Lang [119] and Szekeres [122].

The map $\Gamma \left(x^{1},x^{2}\right)$ shown in Figure 3 is a smooth, one-to-one, onto, invertible map between the 2D Euclidean space with Cartesian coordinates $\left(x^{1},x^{2}\right)$ and the 2D submanifold $\Gamma \left(x^{1},x^{2}\right)$ embedded in the configuration manifold C. It maps coordinate axes $x^{1}$ and $x^{2}$ to curved horizontal and vertical geodesic coordinate axes $\alpha_{0}\left(x^{1}\right)$ and $\beta_{0}\left(x^{2}\right)$, respectively. The metric-distance $x^{2}$ is constant along each of the horizontal geodesic coordinate grid lines $\Gamma_{x^{2}}\left(x^{1}\right)=\alpha_{x^{2}}\left(x^{1}\right)$ while the metric-distance $x^{1}$ is constant along each of the vertical coordinate grid lines $\Gamma_{x^{1}}\left(x^{2}\right)=\beta_{x^{1}}\left(x^{2}\right)$. $X=\frac{\partial \Gamma_{x^{2}}\left(x^{1}\right)}{\partial x^{1}}$ and $Y=\frac{\partial \Gamma_{x^{1}}\left(x^{2}\right)}{\partial x^{2}}$ are vector fields tangent to the horizontal and vertical coordinate grid lines, respectively. Because they are tangent to *coordinate* grid lines on the submanifold their Lie bracket $\left[X,Y\right]=\nabla_{X}Y-\nabla_{Y}X$ equals zero; that is, $\nabla_{X}Y=\nabla_{Y}X$. Taking a second tensorial differential we obtain $\nabla_{X}^{2}Y=\nabla_{X}\nabla_{Y}X$. From the equation for the curvature endomorphism $R\left(X,Y\right)$ we have $R\left(X,Y\right)X=\nabla_{X}\nabla_{Y}X-\nabla_{Y}\nabla_{X}X=\nabla_{X}\nabla_{Y}X$ because $\nabla_{X}X=0$ along all horizontal geodesicss $\alpha_{x^{2}}\left(x^{1}\right)$. Combining these two equations we obtain the Jacobi equation for the Jacobi vector field Y (i.e., Jacobi lift) along the horizontal geodesic coordinate axis $\alpha_{0}\left(x^{1}\right)$

$$\nabla_{X}^{2}Y=R\left(X,Y\right)X$$

The Jacobi equation is a second-order ordinary differential equation so, to obtain a unique solution for the Jacobi vector field $Y\left(x^{1}\right)$, two initial conditions $z=Y\left(0\right)$ and $w=\nabla_{X}Y\left(0\right)$ are required. The initial vectors z and w together with the initial velocity vector $\dot{\alpha_{0}}=e_{1}$ specify the submanifold $\Gamma \left(x^{1},x^{2}\right)$. It follows that $116^{3}$ different submanifolds $\Gamma \left(x^{1},x^{2}\right)$ centred about the specified initial configuration $c_{i}$ are possible depending on the selection of z, w and $e_{1}$. If the mass-inertia matrix $J\left(c\right)$ is known, the Riemannian geometry equations $\Gamma_{jk}^{i}=\frac{1}{2}J^{im}\left(J_{mj,k}+J_{mk,j}-J_{jk,m}\right)$ and $R_{ijkl}=J_{lp}R_{ijk}^{p}=J_{lp}\left(\Gamma_{kj,i}^{p}-\Gamma_{ki,j}^{p}+\Gamma_{kj}^{m}\Gamma_{mi}^{p}-\Gamma_{ki}^{m}\Gamma_{mj}^{p}\right)$ can be used to obtain the curvature endomorphism $R\left(X,Y\right)X$. Hence knowing the mass-inertia matrix $J\left(c\right)$ allows the Jacobi equation to be solved to obtain the Jacobi vector field $Y\left(x^{1}\right)$ along the geodesic $\alpha_{0}\left(x^{1}\right)$. In fact, since the orthonormal vectors $\left(e_{1},e_{2}\right)$ can be parallel translated along the vertical geodesic coordinate axis $\beta_{0}\left(x^{2}\right)$ as illustrated in Figure 3, the vector field $Y\left(x^{1}\right)$ can be computed along each of the horizontal geodesic coordinate grid lines $\alpha_{x^{2}}\left(x^{1}\right)$.

An insight into the properties of $\Gamma \left(x^{1},x^{2}\right)$ comes from the fact that the inner product $\langle X,Y\rangle_{J}$ varies linearly with distance $x^{1}$ along the geodesic $\alpha_{0}\left(x^{1}\right)$. The intercept with the $\langle X,Y\rangle_{J}$ axis at $x^{1}=0$ is given by $\langle z,e_{1}\rangle_{J}$ and the slope of the linear graph is given by $\langle w,e_{1}\rangle_{J}$. This follows from the observation that $\nabla_{X}^{2}\langle Y,X\rangle_{J}=\langle \nabla_{X}^{2}Y,X\rangle_{J}=\langle R\left(X,Y\right)X,X\rangle_{J}=R_{m}\left(X,Y,X,X\right)=0$. The last equality follows from the symmetry properties of $R_{m}$. Since the acceleration $\nabla_{X}^{2}\langle Y,X\rangle_{J}$ of $\langle Y,X\rangle_{J}$ along $\alpha_{0}\left(x^{1}\right)$ is zero it follows that the velocity (slope) of $\langle Y,X\rangle_{J}$ along $\alpha_{0}\left(x^{1}\right)$ is constant and equal everywhere to its value $\langle w,e_{1}\rangle_{J}$ at $x^{1}=0$. Thus if we set z= $e_{2}$ and w=0 (as described in the text and illustrated in Figure 3), the Jacobi vector Y will be J-orthogonal to the vector X at every point $x^{1}$ along $\alpha_{0}\left(x^{1}\right)$. Indeed Y will be J-orthogonal to the vector X everywhere on $\Gamma \left(x^{1},x^{2}\right)$. Setting z= $e_{2}$ and w=0 restricts the number of possible submanifolds $\Gamma \left(x^{1},x^{2}\right)$ that can be generated but there still exist $116^{2}$ submanifolds $\Gamma \left(x^{1},x^{2}\right)$ to choose from, depending on the specified orthonormal $e_{1}$ and $e_{2}$.

Since the horizontal geodesic coordinate grid lines are unit metric-speed geodesics it follows that the vector field X tangent to the horizontal geodesic coordinate grid lines $\alpha_{x^{2}}\left(x^{1}\right)$ is a unit vector at every point $\left(x^{1},x^{2}\right)$ in $\Gamma \left(x^{1},x^{2}\right)$. The vector Y tangent to the vertical coordinate grid lines $\beta_{x^{1}}\left(x^{2}\right)$ in the submanifold is not a unit vector. Its norm ∥Y∥J=〈Y,Y〉J12 is determined by the local deviation of the horizontal geodesic coordinate grid lines from each other.

The norm ∥Y∥J=〈Y,Y〉J12 of the Jacobi vector Y and the way it varies with $x^{1}$ along the geodesic coordinate grid lines $\alpha_{x^{2}}\left(x^{1}\right)$ can be obtained by setting $f\left(x^{1}\right)=$ 〈Yx1,Yx1〉J12 and computing the first and second derivatives $f^{\prime }\left(x^{1}\right)$ and $f^{\prime \prime }\left(x^{1}\right)$ as a function of $x^{1}$. We obtain $f^{\prime }\left(x^{1}\right)=\frac{1}{∥Y{∥}_{J}}\langle \nabla_{X}Y,Y\rangle_{J}\;\mathrm{and}\;f^{\prime \prime }\left(x^{1}\right)=\frac{1}{∥Y{∥}_{J}{}^{3}}\left[{\left(\nabla_{X}Y\right)}^{2}{\left(Y\right)}^{2}-\langle \nabla_{X}Y,Y\rangle_{J}{}^{2}\right]+\frac{1}{∥Y{∥}_{J}}\left[R_{m}\left(X,Y,X,Y\right)\right]$. From these expressions it can be seen that if $R_{m}\left(X,Y,Y,X\right)$ is negative (i.e., curvature is negative) the norm ∥Y∥J=〈Y,Y〉J12 of the Jacobi vector Y increases with $x^{1}$ and the local horizontal geodesics diverge. On the other hand, if $R_{m}\left(X,Y,Y,X\right)$ is sufficiently positive (i.e., curvature is sufficiently positive) for $f^{\prime \prime }\left(x^{1}\right)$ to be negative, the norm ∥Y∥J=〈Y,Y〉J12 of the Jacobi vector Y along $x^{1}$ can initially increase, reach a maximum, and then decrease causing the horizontal geodesics to converge and eventually to cross. Thus by setting the initial conditions to z= $e_{2}$ and w=0 and limiting the arc length of the geodesics $\alpha_{x^{2}}\left(x^{1}\right)$ so they do not cross, we obtain a submanifold $\Gamma \left(x^{1},x^{2}\right)$ that approaches as closely as possible to a Cartesian submanifold in Euclidean space given the curvature of the configuration manifold $\left(C,J\right)$. As can be seen from the equations for $\Gamma_{jk}^{i}$ and $R_{ijkl}$ above, the curvature $R_{m}\left(X,Y,Y,X\right)$ of the submanifold $\Gamma \left(x^{1},x^{2}\right)$ is determined by covariant derivatives of the mass-inertia matrix $J\left(c\right)$.

## Appendix C. Error-Reducing Association Memory Network

A remarkable property of the brain is its ability to form associations between all types of mental images. While a single synapse can act as a memory storage element, its response is too ambiguous for it to serve as an association memory on its own. A storage concept in which associations are stored in a way that is distributed over many synapses is more reliable ([126], p. 32).

Various proposals for *association memory networks* capable of storing associations between multiple temporospatial patterns of neural activity have been explored. Basically these employ correlation-based learning algorithms similar to the stabilized Hebbian synaptic learning rule at each learning step, although recently this has been augmented using Bayesian optimization [127,128,129,130,131,132]. Feedback is almost always present in real nervous systems. As foreseen in the pioneering work of Marr [133] and Hopfield [134] an important property of networks with feedback is that if an incomplete input pattern is applied to an association memory network in which a set of input-output associations has been stored, the intact pattern may be sufficient for reconstruction of the missing data. Such a recurrently interconnected association memory network can recall the complete input pattern on the basis of an incomplete fragment [126] (p. 45). This capability is a prominent feature of biological nervous systems.

We propose the following hypothetical association memory network in the human brain. A large number of cortical neurons of different types and sizes are interconnected both vertically and horizontally in both forward and backward directions by adaptive excitatory and inhibitory interconnections. Vertical connections dominate, forming mini cortical columns across the six layers of the cortex. The cortical columns are reciprocally interconnected both locally and across different regions of the cortex through association fibre tracts. There also exist cortical-subcortical-cortical connections through parts of the basal ganglia and cerebellum. Multiple recurrent pathways have different loop transmission time delays caused by varying numbers of synapses in the loops. Such loops produce a filtering action and can introduce lightly-damped oscillations.

Within this proposed association memory network the individual neurons have correlation-based stabilized Hebbian-like molecular mechanisms of synaptic plasticity. The key property that enables this arrangement to function as an error-reducing network in reinforcement learning is that modification of the component synaptic weights happens only when a modulating transmitter such as dopamine is present. As set out in Section 7 and illustrated in Figure 5 an error signal is obtained elsewhere in the nervous system and a reduction in the error from one learning cycle to the next (i.e., a negative temporal difference in error) is rewarded by release of a burst of dopamine into the association memory network. Thus synaptic modification within the network occurs only when the error reduces.

To elaborate, input and output working memory buffers (formed by ensembles of neurons that can hold spatial patterns of activity on-line) are reciprocally interconnected in the network. These provide the main source of network activity. Sustained activity held within these buffers gives rise to propagating waves that induce interference patterns on synapses distributed throughout the network. If dopamine is present this interference changes the synaptic weights, thereby changing the pattern of activity in the network. This in turn changes the synaptic weights in an iterative fashion and the process converges to a stable limit cycle pattern of activity in the network [135,136,137]. Convergence to a stable limit cycle has been demonstrated in a Ph.D. project in our lab using simulated networks of randomly interconnected model neurons [138,139]. Similar networks that evolve towards stable stored states known as attractors have been proposed for the hippocampal formation (for review see [44]).

The converged synaptic weights distributed throughout the network effectively associate the sustained patterns of neural activity held on-line in the input and output working memory buffers. If an incomplete pattern of neural activity is held on-line in the working memory buffers the induced limit cycle pattern of activity in the network completes the pattern of activity. Unsupervised learning within the network modulated by dopamine thus effectively associates a pattern of activity held on-line in an input working memory buffer with a pattern of activity held on-line in an output working memory buffer.
